# Supplementary material for: Membrane Tension Orchestrates Rear Retraction in Matrix-Directed Cell Migration
Source: Dev Cell. 2019 Nov 18;51(4):460–475.e10. doi: 10.1016/j.devcel.2019.09.006 (PMC6863396; doi:10.1016/j.devcel.2019.09.006)
Supplement: Document S1. Figures S1–S7 [file mmc1.pdf]

Developmental Cell, Volume *51*

Supplemental Information

Membrane Tension Orchestrates Rear Retraction in Matrix-Directed Cell Migration

Joseph H.R. Hetmanski, Henry de Belly, Ignacio Busnelli, Thomas Waring, Roshna V. Nair, Vanesa Sokleva, Oana Dobre, Angus Cameron, Nils Gauthier, Christophe Lamaze, Joe Swift, Aránzazu del Campo, Tobias Starborg, Tobias Zech, Jacky G. Goetz, Ewa K. Paluch, Jean-Marc Schwartz, and Patrick T. Caswell

Supplementary information

Figure S1-S7

Video S1-7

Supplementary Figure Legends

Figure S1: 3D migration in fibrillar matrix; related to Figure 1

(A) A2780 cells seeded within 3D CDM. Representative single plane images above, through and below the cell (top) and an xz projection s (bottom), where each dashed line denotes the relative positions of the single plane images. Cells/matrix were fixed and stained for fibronectin and F-actin (SiR-actin) prior to imaging with a Z step size of 0.27 µm. (B) A2780 cells moving in an unperturbed CDM (left) or a locally softened CDM (right), representative maximum intensity projections (MIPs) shown. Cells were fixed and imaged as in A. (C) H1299 cells were seeded onto FN-coated 2D durotactic gradients for 16 hours and imaged, orientation of migration with respect to the gradient is shown by rose plot. (D) Left: A2780 cells expressing farnesyl-EGFP (GFPmembrane) were seeded onto 3D CDM and imaged as in Fig. 1A. Right: Pairwise quantification of normalised GFP-membrane peak intensity at the front and rear of A2780 cells (N=33 cells across 3 repeats). (E) A2780 expressing Lifeact-GFP stained with lipophilic dye (DiI) moving in 3D CDM. Timelapse of the cell rear captured using Zeiss Airyscan. (F) Trap force at the front and rear of unpaired cells on durotactic gradients, untreated glass and uniform stiff gels and single membrane measurements of unpolarised cells on uniform soft gels; N>31 cells per condition analysed across 3 repeats for gradient; N>18 per condition across 3 repeats for glass, N>12 cells per condition across 3 repeats for uniform stiff, N=6 cells across 2 repeats for uniform soft. (G) A2780 cells expressing EGFP-paxillin and mCherry-caveolin-1 were seeded in CDM for 4 hours then imaged for 5 minutes, 9 Z-planes imaged every 1µm by spinning-disc confocal microscopy, representative MIPs of merged (left) and paxillin (right) channels shown; Right: Pairwise quantification of number of adhesions in front/nucleus region compared to behind nucleus region, N=31 cells across 3 repeats. ****, p<0.0001.

Figure S2: Caveolae localization in cells in 3D matrix and durotactic gradients; related to Figure 2

(A) H1299 cells expressing Lifeact-mEmerald and mCherry-caveolin-1 were seeded in 3D CDM and imaged as in Fig. 2(B). (B) H1299 non-small cell lung cancer cells were seeded in 3D CDM and fixed and stained for F-actin and cavin-1. MIPs of confocal stacks are shown. (C) A2780 cells seeded on a CDM locally softened via trypsin beads, fixed as in B and stained for F-actin (SiR-actin) and cavin-1. Representative MIP shown. (D) A2780 cells in 3D CDM were fixed and stained for endogenous caveolin-1. Representative MIP of confocal stacks are shown. (E) H1299 cells expressing Lifeact-mEmerald and mCherry-caveolin-1 were seeded on 2D durotactic gradients and imaged as in Fig. 2(C). (F) A2780 cells on 2D soft, stiff, or durotactic gradients of stiffness (all coated with fibronectin) stained for F-actin (phalloidin) and cavin-1. Representative MIP shown. (G) A2780 cells on 2D soft, stiff, or durotactic gradients of stiffness (all coated with fibronectin) stained for F-actin (SiR-actin) and caveolin-1. Representative MIP shown. (H) Orientation of caveolin-1 in cells as in (F) with respect to the stiffness gradient (N=30 cells across 3 repeats). (I) Average speed of A2780, H1299, Telomerase-immortalized fibroblasts (TIFs) or mouse embryonic fibroblasts (MEFs) cells in 3D CDM during 16 hours timelapse. (J) Average intensity of mCherry-caveolin-1 at the rear of A2780, H1299, MEF and TIF cells moving in 3D CDM. (K) TIFs or MEFs were seeded in 3D CDM and fixed stained and imaged as in (B), representative images of poorly polarized highly spread morphology of TIFs and MEFs in 3D CDM. (L) Trap force measurements at the front and rear of elongated TIF cells, N=10 cells across 3 repeats. (M) TIFs and MEFs as in (K), displaying the polarised, shorter phenotype. (N) Morphology of TIFs and MEFs when undergoing rapid migration in 3D CDM. (O) Proportion of TIFs and MEFs that show elongated (typical) morphology versus shortened (rapid) morphology, and proportion of cells that exhibit high versus low/unpolarized cavin-1 at the cell rear. (P) A2780 cells expressing mEmerald-Lifeact and mCherry-caveolin-1 were imaged migrating in 3D CDM, and fixed and processed for Serial Block Face SEM (SBFSEM). The relative ease of data collection using SBFSEM meant that it was possible to generate correlative EM data from 9 different motile cells with sufficient resolution to allow identification of caveolae. A volume render of the EM data is compared with the light microscope image to show that the cell morphology has not been affected by fixation. The enlargements show evidence of

caveolae/multilobed caveolae (yellow arrowheads) at the rear of the cell, but no evidence of caveolae was seen in the peri-nuclear region. ****, p<0.0001.

Figure S3: Increasing membrane tension supresses rear caveolae assembly in durotactic gradients and CDM; related to Figure 3

(A) A2780 cells expressing Lifeact-EGFP and mCherry-caveolin-1 were seeded onto 2D durotactic gradients and imaged before and during osmotic shock treatment (0.5Xmedia). (B) A2780 cells were seeded onto CDM and treated with osmotic shock (0.5Xmedia) or isotonic media, before fixation, staining and imaging of endogenous caveolin-1. MIPs are shown. Yellow line indicates outline of the cell. Insets MIPs of the cell rear captured using high resolution imaging. (C) Distance of rear retraction of A2780 cells in isotonic versus hypo-osmotic shock in a 5 minute timelapse. N>61 cells per condition across 3 repeats. ****, p<0.0001.

Figure S4 Caveolae control rear retraction through RhoA-ROCK1/PKN2 actin organisation; related to Figure 4

(A) Caveolin-1 knockdown A2780 cells were plated in 3D CDM and speed of migration measured by timelapse microscopy over 16 hours. N=90 cells per condition across 3 repeats. (B) Representative images of control and caveolin-1 knockdown A2780 cells moving through 3D CDM. (C) Quantification of control and caveolin-1 knockdown cell length whilst migrating in 3D CDM. N=90 cells analysed per condition across 3 repeats. (D) Efficiency of knockdown of caveolin-1 in A2780 cells. (E) Efficiency of knockdown of caveolin-1 in H1299 cells. (F) Left: Lifeact-mRFP expressing, caveolin-1 knockdown H1299 cells were plated in 3D CDM, imaged by spinning disk confocal microscopy and retraction of the cell rear over 5 minutes measured. Right: Forward movement of H1299s cells over a 5 minute timelapse. N>74 cells per condition across 3 repeats. (G) Left: Control and EHD2 knockdown A2780 cells expressing Lifeact-EGFP and mCherry-caveolin-1 migrating in 3D CDM imaged by spinning disk confocal microscopy. Centre: Average migration speed of control and EHD2 knockdown cells migrating in 3D CDM for 16 hours. N=90 cells analysed per condition across 3 repeats. Top right: Distance of rear retraction of control and EHD2 knockdown A2780 cells in 3D CDM during a 5 minute timelapse N>80 cells per condition across 3 repeats. Bottom right: EHD2 knockdown efficiency in A2780 cells. (H) Left: Representative images of control and EHD2 knockdown A2780 cells with coloured bars indicating cell length. Right: Quantification of control and EHD2 knockdown cell length whilst migrating in 3D CDM. N=90 cells analysed per condition across 3 repeats. (I) Active RhoA location biosensor GFP-AHPH expressing A2780 cells were seeded in 3D CDM and fixed after 6 hours. F-actin was visualized with SiR-actin, and cells imaged at high resolution. MIPs are shown. (J) Active RhoA location biosensor GFP-AHPH expressing A2780 cells were seeded on 2D durotactic gradients for 16 hours and imaged by spinning disk microscopy using a long working distance 63x objective. (K) Active RhoA location biosensor GFP-AHPH and mCherry-caveolin-1 expressing A2780 cells were seeded in 3D CDM and imaged by spinning disk confocal microscopy after 6 hours. (L) Raichu-RhoA in osmotic shock. (M) Left: Control or RhoA knockdown cells were seeded into 3D CDM and imaged by spinning disk confocal microscopy after 4 hours. Single z-slice stills from movies are shown 300s apart. Centre: Forward movement of control and RhoA knockdown cells over a 5 minute timelapse. N>54 cells per condition across 3 repeats. Right: RhoA knockdown efficiency in A2780 cells. (N) Control, ROCK1 or ROCK2 knockdown cells were seeded into 3D CDM and fixed after 4 hours. F-actin was visualized using SiR-actin and confocal imaging. MIPs are shown. (O) A2780 cells were seeded into 3D CDM and treated with Y27632 (10 µM) for 2 hours before fixation. F-actin (phalloidin) was visualized by confocal microscopy, MIPs shown. (P) Left: Speed of migration of A2780 cells in 3D CDM over 16 hour timelapse was analysed in control, ROCK1, ROCK2 and ROCK1+2 knockdown cells. N=90 cells analysed per condition across 3 repeats. Right: Efficiency of ROCK1 and ROCK2 knockdown in A2780 cells. (Q) Top left: Speed of migration of A2780 cells in 3D CDM over 16 hour timelapse was analysed in control, PKN1, PKN2 and PKN3 knockdown cells N=90 cells analysed per condition across 3 repeats. Top right: Effect of PKN2 knockdown on architecture of F-actin at the rear of A2780 cells migrating in 3D CDM (representative high resolution MIP shown). Bottom left: Efficiency of PKN1, PKN2 and PKN3 knockdown in A2780 cells, assessed using a pan-pPKN antibody. Quantification of blots from 3 independent experiments shown in graph. Bottom right: Efficiency of PKN2 knockdown in A2780 cells, assessed using a PKN2 specific antibody. ****, p<0.0001; *, p<0.05.

Figure S5: Systematic identification of GEFs activating RhoA at the cell rear, related to Figure 5

(A) Quantification of average speed of control, Ect2, Vav2, ARHGEF2, DOCK1, ELMO2 and PLEKHG5 A2780 cells in long-term (16h) migration in 3D CDM, N>60 cells per condition across 3 repeats, dashed line indicates control mean. (B) Quantification of cell length ~20h after spreading for cells is in (A), N>60 cells per condition across 3 repeats. (C) Quantification of average speed of control and 2 individual Ect2 oligo knockdown A2780 cells, N=30 cells analysed per condition across 2 repeats. (D) Representative single control and Ect2 knockdown cells migrating in 3D CDM over 150 minutes. (E) Quantification of rear / front peak F-actin staining intensity of control, and Ect2 knockdown cells (individual oligos). (F) Efficiency of knockdown of Ect2, Vav2, ARHGEF2, DOCK1, ELMO2 and PLEKHG5 in A2780 cells. (G) Knockdown of Ect2 with individual siRNA oligos; #5 and #7 were used in subsequent experiments. ****, $p<0.0001$; *, $p<0.05$.

Figure S6: Model outputs are robust to wide parameter value ranges; related to Figure 6

(A) Summary parameter sensitivity analysis showing normalised effect of all rates in the model on the rear retraction output. Parameters which have > 0.02 positive scaled effect on rear retraction are shown in green, parameters which have < 0.02 negative scaled effect shown in red, while all other parameters with less drastic effects are represented by the blue bars. (B) Range of rear retraction temporal dynamics given halving and doubling of the 9 most influential rates in the model (6 positive regulators in green, 3 negative regulators in red) showing general model outputs are robust to even large parameter alterations.

Figure S7: Effect of RhoA knockdown and CytoD on caveolae localization; related to Figure 7

(A) Control and RhoA knockdown A2780 cells expressing mCherry-caveolin-1 were seeded into 3D CDM and imaged by spinning disk confocal microscopy after 4 hours. Single z-slice stills from movies are shown 300s apart. (B) Intensity of mCherry-caveolin-1 at the rear of cells as in (A) N=76 cells per condition across 3 repeats. (C) Control and RhoA knockdown cells were seeded into 3D CDM and fixed after 4 hours. Endogenous F-actin, caveolin-1 and cavin-1 were visualized, representative MIPs shown. (D) High resolution images of endogenous cavin-1 at the rear of control and RhoA knockdown A2780 cells migrating in 3D CDM as in (C). (E) Quantification of the average rear/front cavin-1 intensity ratio of control and individual oligo Ect2 knockdown A2780 cells. (F) A2780 cells expressing Lifeact-EGFP and mCherry-caveolin-1 were seeded in 3D CDM for 4 hours before and imaging in the presence of 2μM CytoD. (G) Caged CytoD is a photoactivatable derivative of the potent actin disruptor Cytochalasin D (CytoD), which possesses nitroveratryloxycarbonyl (Nvoc) photoremovable group located at the bioactive hydroxyl group of CytoD. The Nvoc group inhibits the binding activity of CytoD, which can be restored upon photolysis reaction with light exposure under microscopy setups. Upon light exposure, caged CytoD enables dosed delivery of CytoD in living cells at time scale of seconds and with subcellular resolution. (H) A2780 cells expressing Lifeact-GFP and mCherry-caveolin-1 were seeded onto 3D CDM and imaged after 4 hours in the absence of caged-CytoD before and after photomanipulation of a specific region behind the cell rear (indicated by yellow box, as in Figure 6G). ****, $p<0.0001$.

Supplementary Video Legends

Video S1: (Part I) A2780 cell expressing mEmerald-Lifeact migrating in 3D CDM over 10 minutes, with a Red-hot LUT applied. (Part II) A2780 cell expressing mEmerald-Lifeact migrating for 5 minutes in a non-uniform CDM with a (trypsinised) softened region to the left and the unperturbed stiffer region to the right. (Part III) A2780 cell expressing mEmerald-Lifeact migrating from soft (left) to stiff (right) on a gradient poly-acrylamide gel for 10 minutes. Related to Figure 1A, B and C respectively

Video S2: A2780 cell expressing farnesyl-EGFP (GFP membrane) migrating in CDM over 5 minutes. Related to Figure S1D.

Video S3: Single representative A2780, H1299, typical MEF, rapid MEF, typical TIF and rapid TIF migrating over 2 hours in CDM. Related to Figure S2I

Video S4: A2780 cell expressing mCherry-caveolin-1 (magenta) and mEmerald-Lifeact (green) imaged prior to fixation and processing for CLEM. Related to Figure S2P.

Video S5: A2780 cell expressing Cherry-Cav-1 migrating in CDM in isotonic medium for 10 minutes, then subjected to osmotic shock for 10 minutes before medium reversion to isotonic conditions for a further 10 minutes. Related to Figure 3C.

Video S6: A2780 cell expressing Cherry-Cav-1, migrating in CDM untreated for 5 minutes, then treated with Y-27632 and imaged for a further 60 minutes. Related to Figure 7A.

Video S7: A2780 cell expressing Cherry-Cav-1 (magenta) and eGFP-Lifeact (green) migrating in CDM treated with 'caged' Cytochalasin-D which is photo-activated at t = 30s within the yellow box region. Related to Figure 7H.

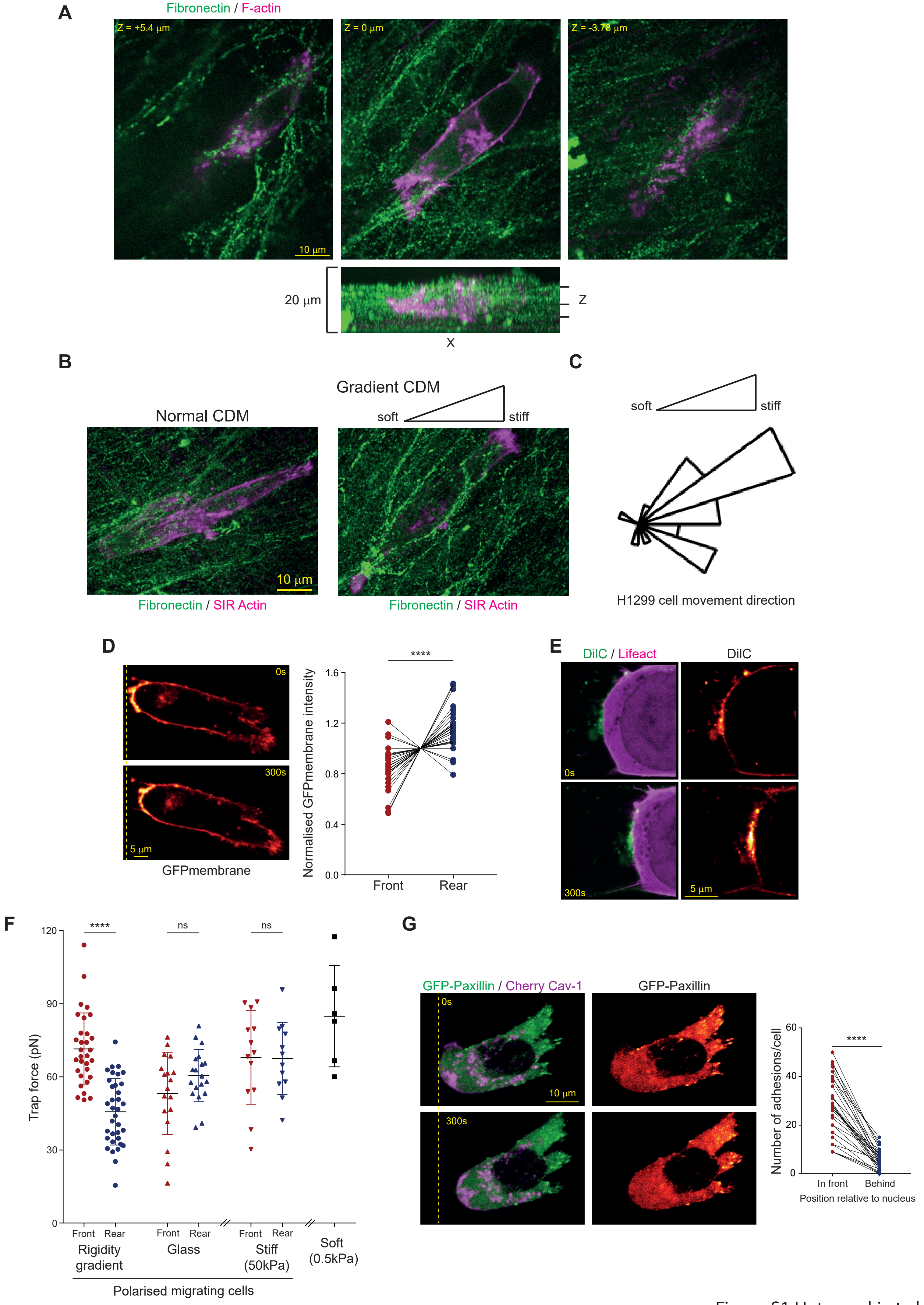

Figure S1 Hetmanski et al

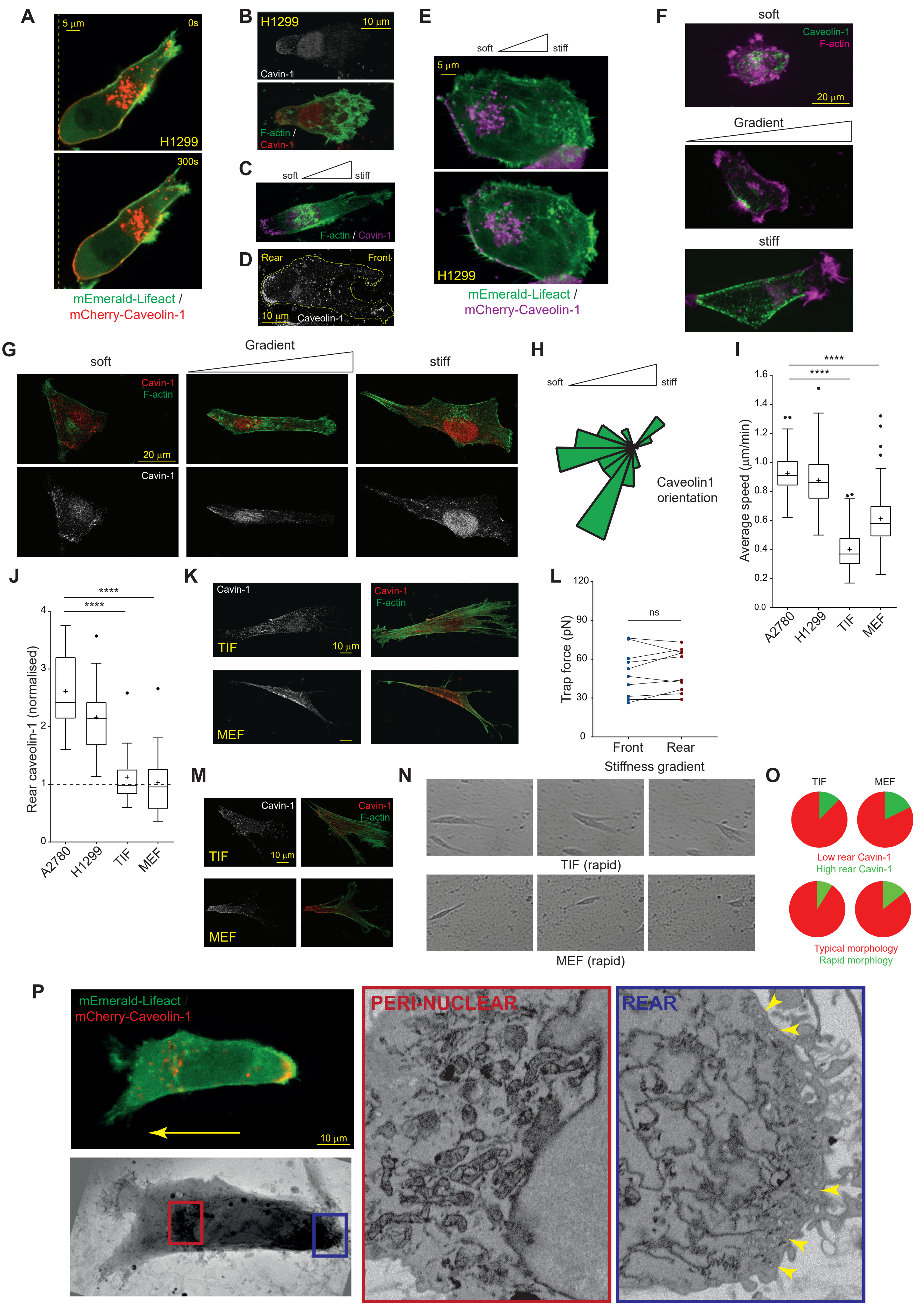

Figure S2 Hetmanski et al

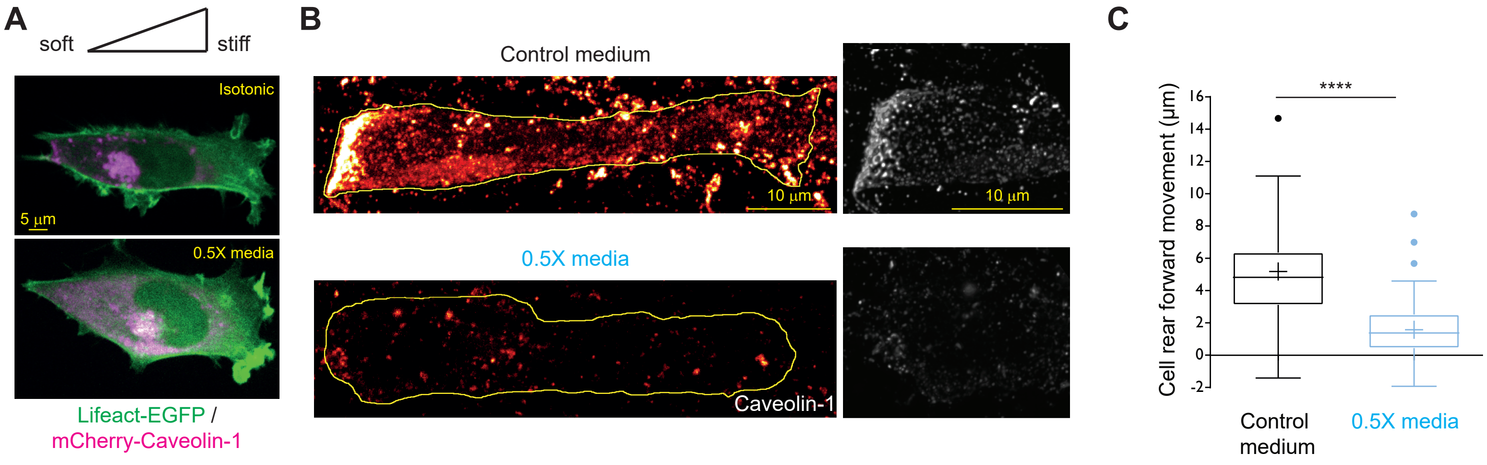

Figure S3 Hetmanski et al

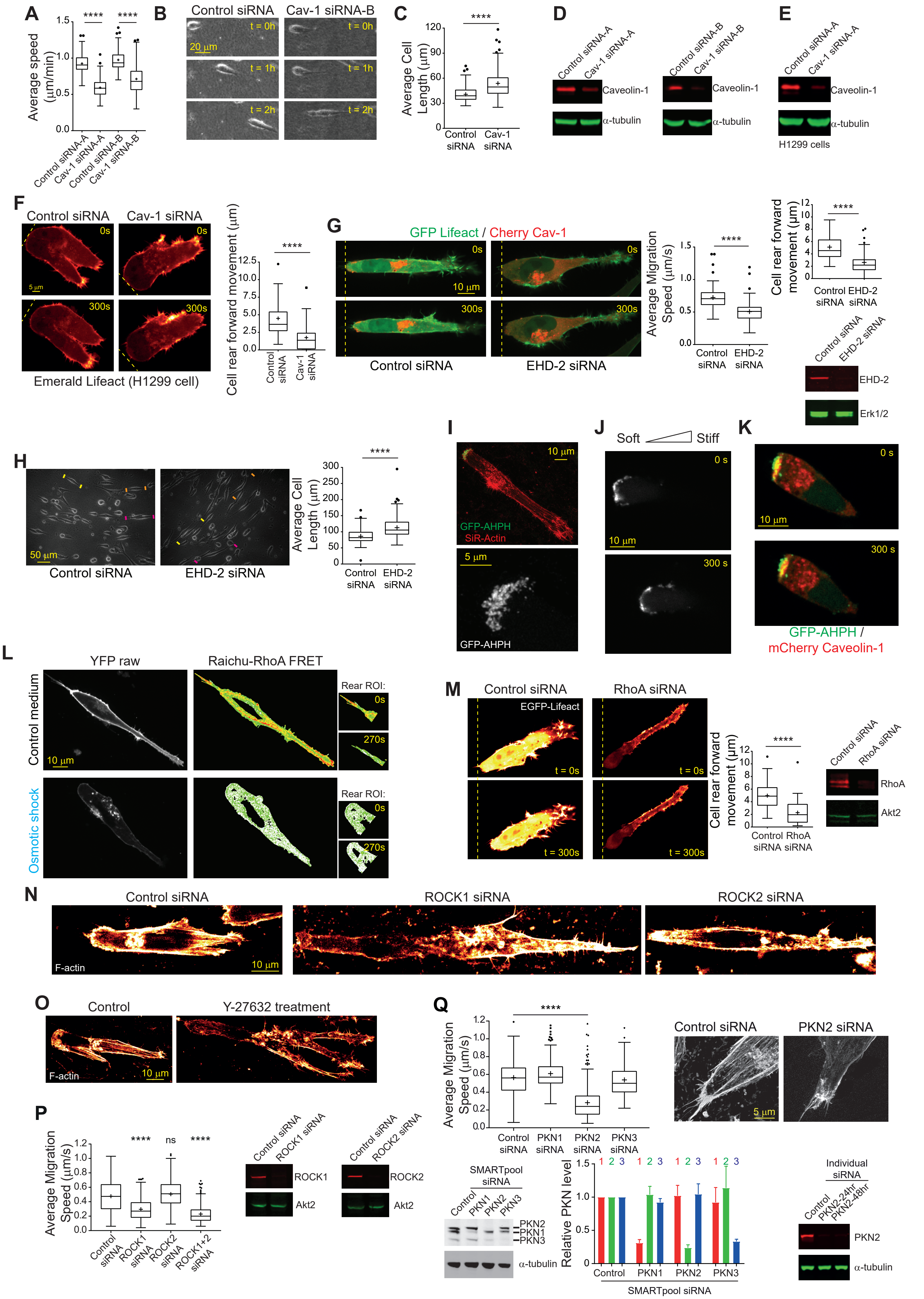

Figure S4 Hetmanski et al

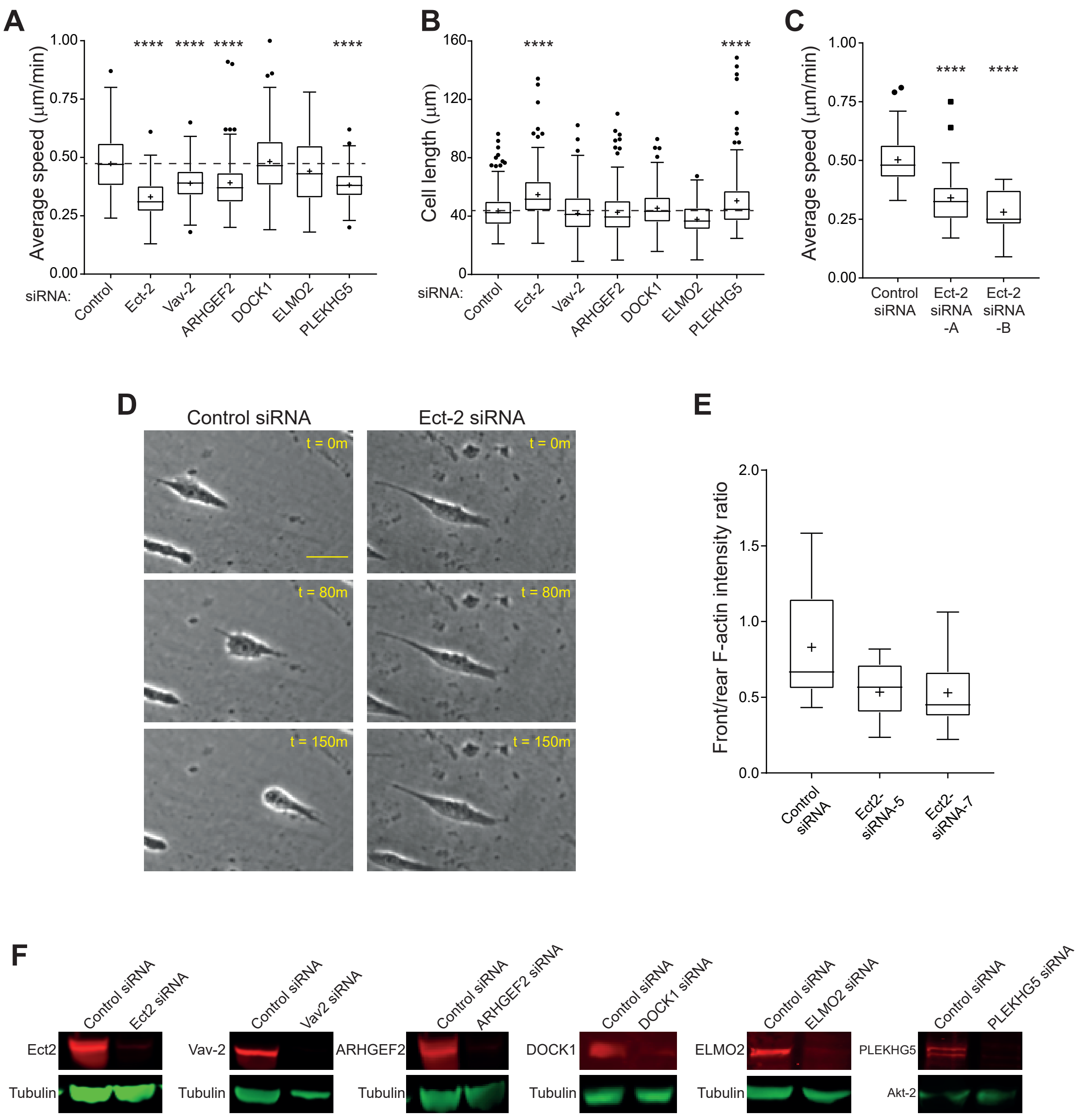

Figure S5 Hetmanski et al

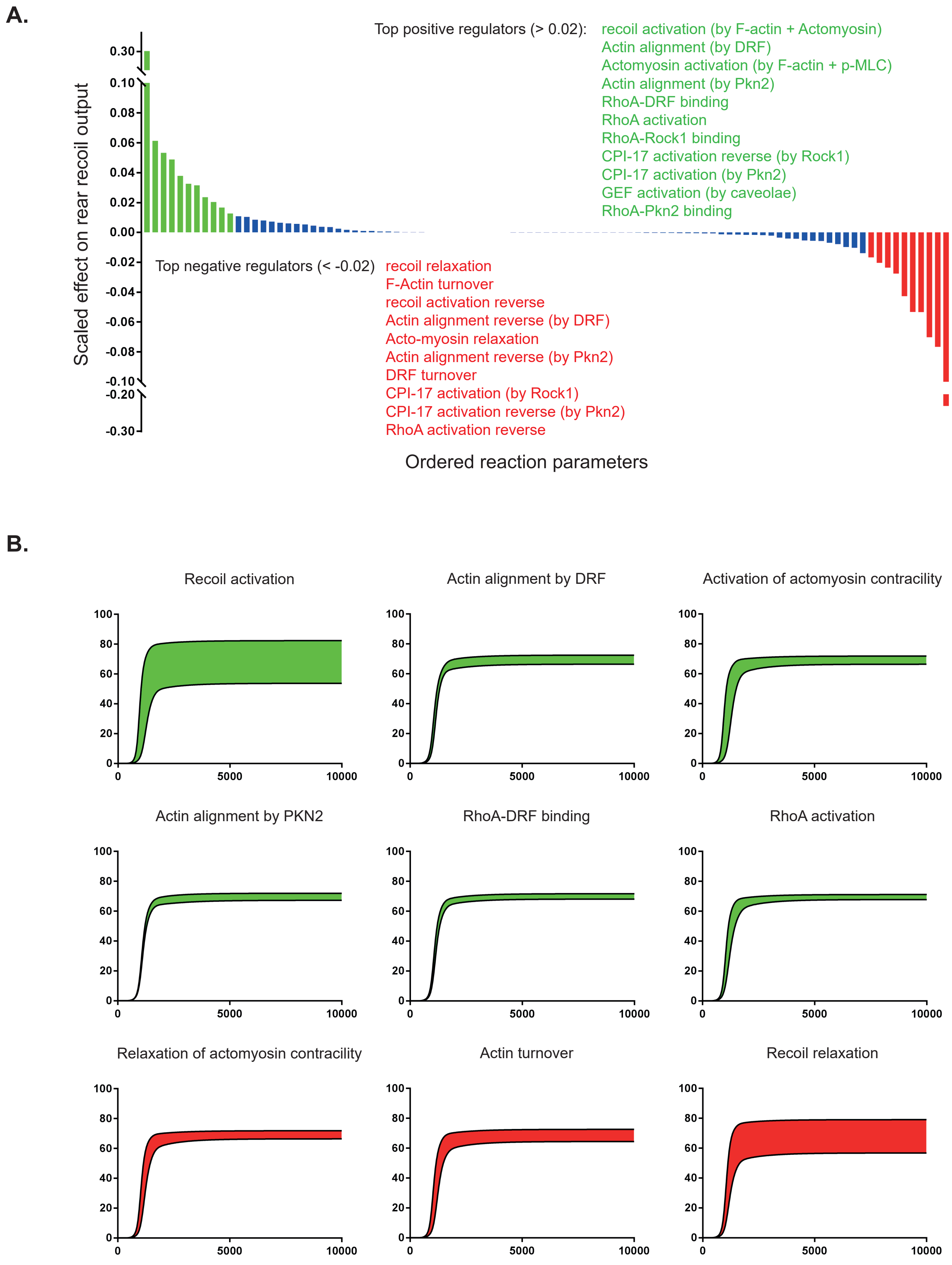

Figure S6 Hetmanski et al

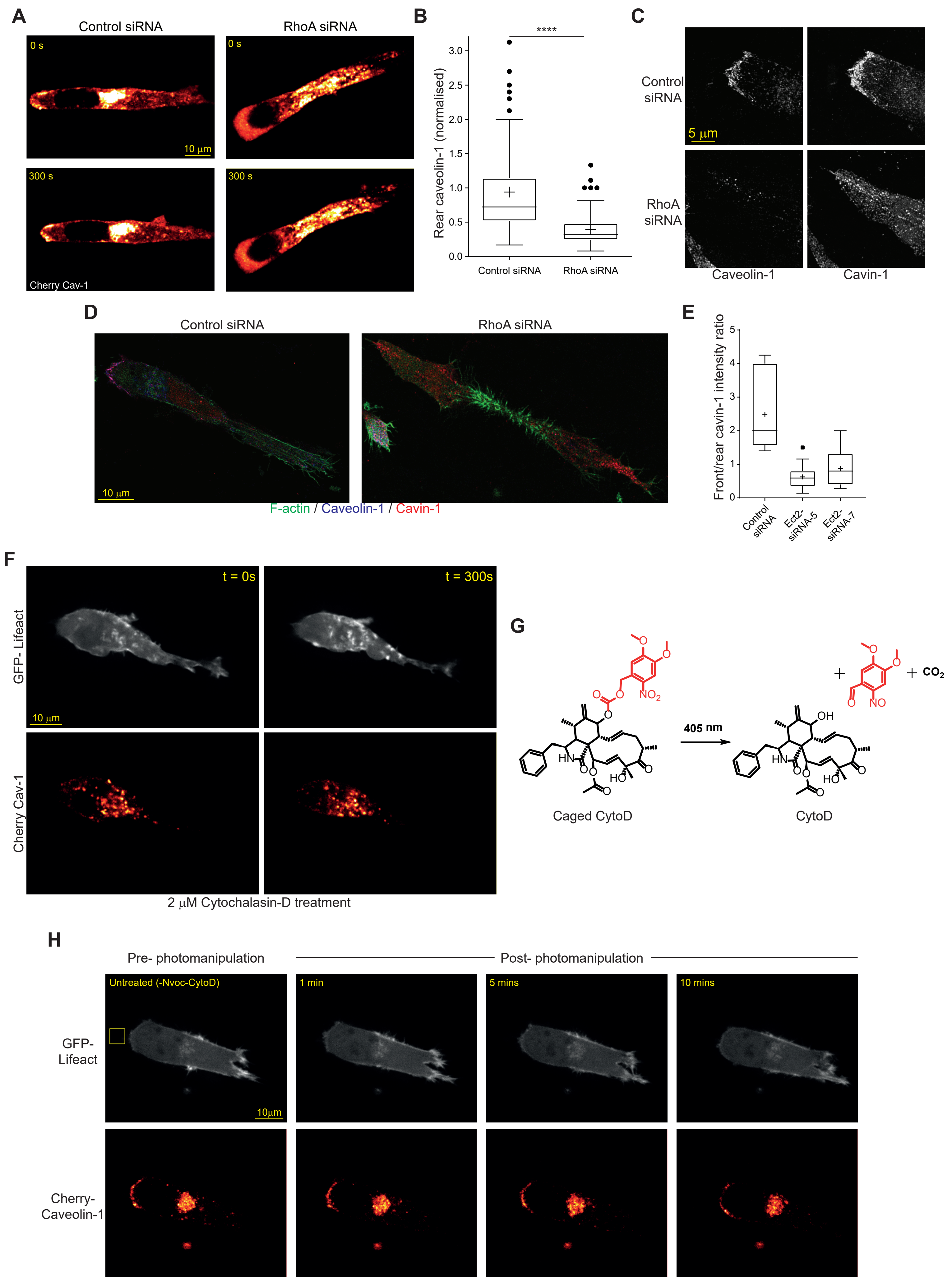

Figure S7 Hetmanski et al
