# Supplementary material for: Membrane Tension Orchestrates Rear Retraction in Matrix-Directed Cell Migration
Source: Dev Cell. 2019 Nov 18;51(4):460–475.e10. doi: 10.1016/j.devcel.2019.09.006 (PMC6863396; doi:10.1016/j.devcel.2019.09.006)
Supplement: Document S2. Article plus Supplemental Information [file mmc9.pdf]

Article

Developmental Cell

Membrane Tension Orchestrates Rear Retraction in Matrix-Directed Cell Migration

Highlights

- Fast-moving cells in 3D matrix establish low membrane tension at the rear
- Caveolae form in response to low membrane tension and recruit the GEF Ect2
- Ect2 activates RhoA to promote F-actin organization and rear retraction
- Positive feedback between membrane tension and contractility reinforces retraction

Authors

Joseph H.R. Hetmanski, Henry de Belly, Ignacio Busnelli, ..., Ewa K. Paluch, Jean-Marc Schwartz, Patrick T. Caswell

Correspondence

patrick.caswell@manchester.ac.uk

In Brief

Cell migration through 3D matrix is critical to developmental and disease processes, but the mechanisms that control rear retraction are poorly understood. Hetmanski et al. show that differential membrane tension allows caveolae to form at the rear of migrating cells and activate the contractile actin cytoskeleton to promote rapid retraction.

Hetmanski et al., 2019, Developmental Cell *51*, 460–475
November 18, 2019 © 2019 The Authors. Published by Elsevier Inc.
https://doi.org/10.1016/j.devcel.2019.09.006

CellPress

Developmental Cell
Article

Membrane Tension Orchestrates Rear Retraction in Matrix-Directed Cell Migration

Joseph H.R. Hetmanski,[1] Henry de Belly,[2,3] Ignacio Busnelli,[4,5,6] Thomas Waring,[7] Roshna V. Nair,[8] Vanesa Sokleva,[1] Oana Dobre,[1] Angus Cameron,[9] Nils Gauthier,[10] Christophe Lamaze,[11] Joe Swift,[1] Aránzazu del Campo,[5] Tobias Starborg,[1] Tobias Zech,[7] Jacky G. Goetz,[4,5,6] Ewa K. Paluch,[2,3,12] Jean-Marc Schwartz,[1] and Patrick T. Caswell[1,13,*]
[1]Wellcome Trust Centre for Cell-Matrix Research, School of Biological Sciences, Faculty of Biology, Medicine and Health, University of Manchester, Manchester Academic Health Science Centre, Manchester M13 9PT, UK
[2]MRC Laboratory for Molecular Cell Biology, University College London, London WC1E 6BT, UK
[3]Institute for the Physics of Living Systems, University College London, London WC1E 6BT, UK
[4]INSERM UMR_S1109, Tumor Biomechanics, Strasbourg 67200, France
[5]Université de Strasbourg, Strasbourg 67000, France
[6]Fédération de Médecine Translationnelle de Strasbourg (FMTS), Strasbourg 67000, France
[7]Institute of Translational Medicine, Cellular and Molecular Physiology, University of Liverpool, Liverpool L69 3BX, UK
[8]INM, Leibniz Institute for New Materials, Campus D226, 66123 Saarbrücken, Germany
[9]Barts Cancer Institute, Queen Mary University of London, London EC1M 6BQ, UK
[10]IFOM, the FIRC Institute for Molecular Oncology, Milan 20139, Italy
[11]Institut Curie - Centre de Recherche, PSL Research University, CNRS UMR 3666, INSERM U1143, Membrane Dynamics and Mechanics of Intracellular Signaling Laboratory, 75248 Paris Cedex 05, France
[12]Department of Physiology, Development and Neuroscience, University of Cambridge, Cambridge CB2 3DY, UK
[13]Lead Contact
*Correspondence: patrick.caswell@manchester.ac.uk
https://doi.org/10.1016/j.devcel.2019.09.006

SUMMARY

In development, wound healing, and cancer metastasis, vertebrate cells move through 3D interstitial matrix, responding to chemical and physical guidance cues. Protrusion at the cell front has been extensively studied, but the retraction phase of the migration cycle is not well understood. Here, we show that fast-moving cells guided by matrix cues establish positive feedback control of rear retraction by sensing membrane tension. We reveal a mechanism of rear retraction in 3D matrix and durotaxis controlled by caveolae, which form in response to low membrane tension at the cell rear. Caveolae activate RhoA-ROCK1/PKN2 signaling via the RhoA guanidine nucleotide exchange factor (GEF) Ect2 to control local F-actin organization and contractility in this subcellular region and promote translocation of the cell rear. A positive feedback loop between cytoskeletal signaling and membrane tension leads to rapid retraction to complete the migration cycle in fast-moving cells, providing directional memory to drive persistent cell migration in complex matrices.

INTRODUCTION

Cell migration is a fundamental process that underpins development, health, and disease and is characterized by cycles of protrusion of the front and retraction of the rear (Ridley et al., 2003). In a 3D matrix, cells move both individually and collectively (Friedl and Alexander, 2011), and in cancer epithelial-mesenchymal transition (EMT) drives the dissemination of elongated single cells to form distant metastases even at very early stages in tumor development (Harper et al., 2016). While the mechanisms that govern protrusion are well characterized (Caswell and Zech, 2018), how retraction of the cell rear is controlled to complete the migration cycle is still under debate (Cramer, 2013). Contractility driven by the action of non-muscle myosin II (NMII) on actin filaments is key for the generation of force required to actively retract the rear, and RhoGTPase signaling (in particular RhoA) is critical in determining NMII activity (Ridley, 2011). However, the mechanisms that control rear retraction in a restrictive 3D matrix such as the interstitial fibrillar matrix environment encountered by metastatic cancer cells in vertebrates is not well understood.

The plasma membrane is the physical barrier between intracellular and extracellular environments and plays an important role in cell migration by organizing signaling and exerting force (tension) on the underlying cytoskeleton (Diz-Muñoz et al., 2013; Gauthier et al., 2012; Keren, 2011). Membrane tension resists membrane deformation and is established in cells by the in-plane tension in the lipid bilayer and membrane to cortex attachment (Diz-Muñoz et al., 2013). Membrane tension was thought to propagate rapidly across cells, but recent evidence indicates that the cytoskeleton plays a role in resisting membrane flow (Shi et al., 2018), allowing cells to establish differential membrane tension across their surface. Membrane tension has recently been shown to be a mechanical signal that regulates formation of focal adhesion complexes (Pontes et al., 2017) and the organization of actin networks (Mueller et al., 2017) at the leading edge of migrating cells on 2D substrates. Biophysical models have suggested that membrane tension at the cell rear might

This is an open access article under the CC BY license (http://creativecommons.org/licenses/by/4.0/).

promote retraction by exerting force (Keren et al., 2008); however, experimental evidence suggests that membrane tension is lower at the rear of migrating fish keratocytes in 2D (Lieber et al., 2015). In addition, the movement of membrane through endocytic traffic has been postulated to play an important role in gathering membrane from the cell rear for re-utilization at the front (Bretscher, 1984). We set out to determine how the cell rear is actively retracted in mammalian cells moving within the physiological 3D matrix, focusing on the dynamics of the plasma membrane in this subcellular region.

RESULTS

Rear Retraction in 3D Matrix and Durotaxis Is a Fast Dynamic Process

Invasive cancer cells moving within fibrillar collagen and fibronectin 3D cell-derived matrix (CDM; Cukierman et al., 2001) followed the topology of fibrils with defined lamellipodial and filopodial protrusions at the cell front (Caswell and Zech, 2018; Paul et al., 2015) (Figures 1A and S1A), similar to that on 2D substrates. The retracting rear region of the cell was rounded and rapidly translocated with few small retraction fibers, and adhesion complexes or long retraction cables were rarely observed here (Figures 1A and S1G; Video S1). In the absence of a chemotactic gradient, we hypothesized that the direction of migration along matrix fibrils could be determined by the physical properties of the matrix. Indeed, atomic force microscopy (AFM) revealed an increase in matrix rigidity directly in front of moving cells (Figure 1A), suggesting that strain stiffening of matrix by moving cells (Van Helvert and Friedl, 2016) could maintain or generate polarity to guide the direction of cell migration. Local softening of the matrix with magnetic trypsin-coated beads (which did not destroy matrix architecture; Figure S1B; Petrie et al., 2012) established a gradient of rigidity up to 2 mm from the targeted area (Figure 1B), and within this gradient region, cells moved toward the stiffer matrix at a similar rate to cells in untreated matrix (Figure 1B; Video S1). On 2D substrates, cells responded to a steep gradient of rigidity (>30 kPa/mm; Figure 1C) by moving toward the stiffer substrate with a similar rounded and rapidly moving rear that oriented toward the soft substrate (Figures 1C and S1C; Video S1). These data indicate that cells in 3D matrix follow mechanical cues and share morphology with cells migrating in 2D durotactic gradients.

A Front-Rear Membrane Tension Differential in Fast-Moving Cells

The analysis of membrane dynamics at the rear of rapidly moving cells in 3D matrix suggested that over the timescale of rapid retraction the plasma membrane accumulated, but significant internalization of labeled membrane was not observed (Figures S1D and S1E; Video S2). Membrane accumulation could result in lower plasma membrane tension (Diz-Muñoz et al., 2013), and we therefore assessed membrane tension by pulling membrane tethers at the cell front and rear (Lieber et al., 2015; Pontes et al., 2017). Tethers were extracted using an optical trap, and the restoring force on the trap was used as a readout of effective membrane tension, which depends on the in-plane tension and the level of attachment between the membrane and the underlying cytoskeleton (Diz-Muñoz et al., 2013). We found that there was no significant difference in membrane tension at the cell front versus the cell rear in cells migrating on uniform stiff substrates or on glass (Figures 1D, 1E, and S1F). However, in durotactic cells migrating toward stiffer substrate in a 2D rigidity gradient, membrane tension was significantly lower at the cell rear than the front (Figures 1D, 1E, and S1F), suggesting that durotactic cells show front-rear polarity through the establishment of a membrane tension differential.

In order to analyze membrane tension in cells within a 3D matrix (and therefore inaccessible via optical trapping), we used Flipper-TR, a small molecule that intercalates into membranes and indicates in-plane membrane tension through changes in fluorescence lifetime of the probe (Colom et al., 2018). Live cells showed a striking decrease in fluorescence lifetime specifically at the cell rear (Figure 1F), suggesting that in-plane membrane tension is significantly lower at the rear of cells moving in a 3D matrix.

Caveolae Accumulate at the Rear of Fast-Moving Cells

Caveolae are plasma membrane invaginations that can act as a route for endocytosis, but they also play a mechanoprotective role by flattening in response to increased membrane tension (Cheng et al., 2015; Garcia et al., 2017; Lim et al., 2017; Parton and del Pozo, 2013; Sinha et al., 2011). Caveolin-1 (a major component of caveolae) and caveolae have previously been shown to polarize at the rear of migrating endothelial cells, fibroblasts, and neurons (Beardsley et al., 2005; Lentini et al., 2008; Parat et al., 2003), although in metastatic cancer cells, this is not always observed (Urra et al., 2012). We hypothesized that caveolae may form as a consequence of the decrease in membrane tension at the cell rear. We observed caveolin-1 localized to the rear of fast-moving cancer cells in 3D matrix (Figures S1G and S2A). Cavin-1, a critical structural component of caveolae (Parton and del Pozo, 2013), was also found to accumulate around the rear of fast-moving cancer cells in 3D matrix (Figures 2A, S2B, and S2C) and co-localized with caveolin-1 (Figure 2A). Caveolin-1- and cavin-1-containing structures appeared close to the plasma membrane around the rear of cells moving in 3D and on 2D rigidity gradients (Figures 2A–2C and S2D–S2H) and in cancer cells escaping spheroids in 3D matrix (which share the elongated morphology of cells moving in 3D CDM; Figures 2D–2F). Fibroblasts (mouse embryonic fibroblasts [MEFs] and telomerase immortalized fibroblasts [TIFs]) were, on average, slow-moving, elongated, and poorly polarized in 3D matrix (Figures S2I–S2K) and were not persistently motile and did not show a membrane tension differential on 2D rigidity gradients (Figure S2L). However, a proportion of fibroblasts migrated rapidly in 3D matrix with similar morphology to cancer cells and showed cavin-1 accumulation at the cell rear (Figures S2M–S2O; Video S3).

Cavin-1 also co-localized closely with EHD2 (Figure 2G), which stabilizes caveolae at the plasma membrane (Hoernke et al., 2017), suggesting that relatively stable caveolae form at the rear of fast-moving cells. By correlating light microscopy with serial block-face scanning electron microscopy (EM), we observed structures that resembled caveolae where caveolin-1 accumulated at the rear of moving cells but not in other regions of the cells (e.g., perinuclear mCherry-caveolin-1; Figure S2P; Video S4). We correlated light microscopy with higher-resolution

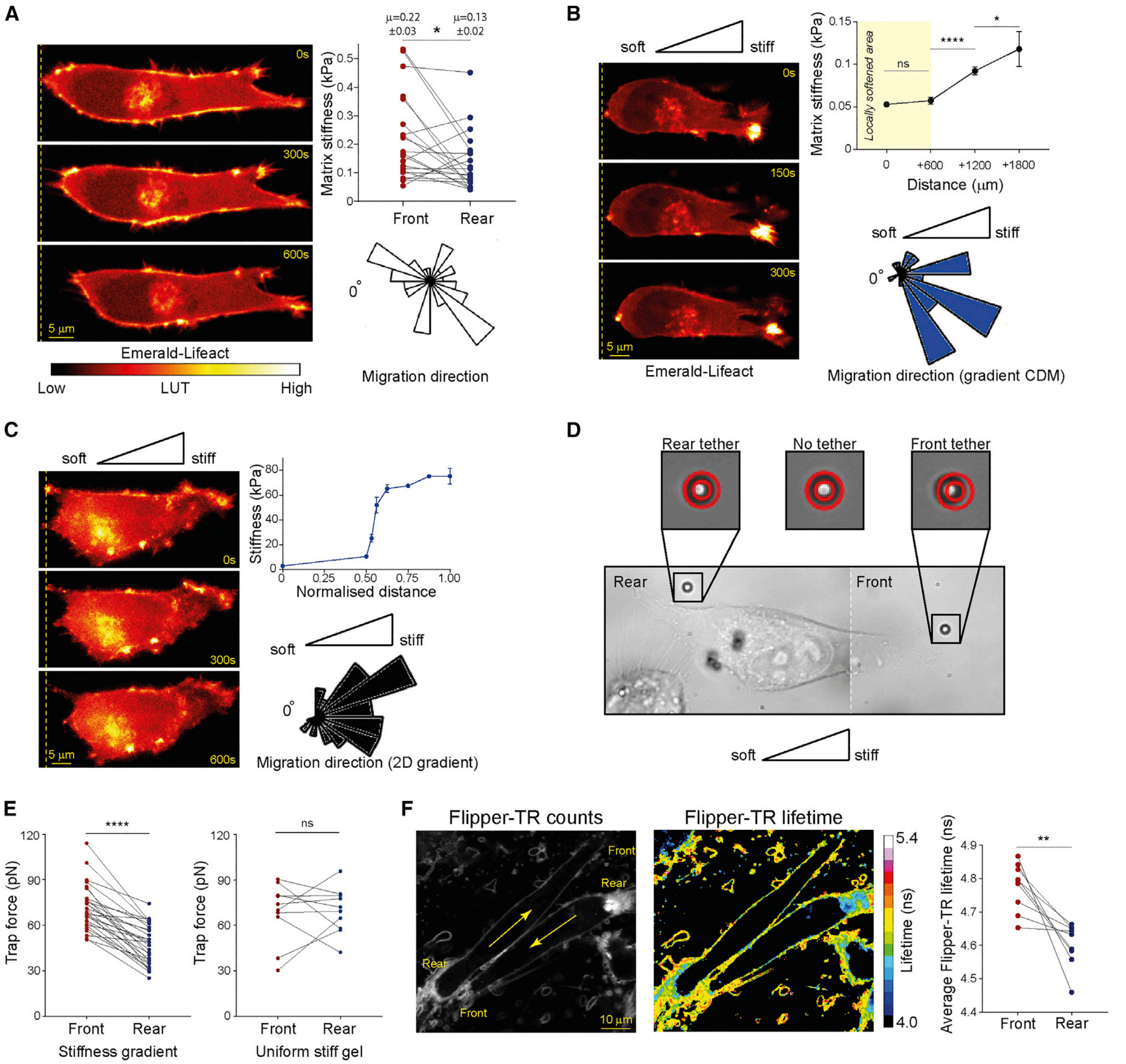

Figure 1. Cell Rear Dynamics in Matrix-Directed Cells

(A) A2780 cells in 3D CDM, confocal sections are shown 300 s apart. Dashed yellow line indicates the rearmost part of the cell in frame 1; Top right: Matrix rigidity immediately in front of and behind cells assessed by AFM, N = 20 cells (3 repeats); bottom right: orientation of migration shown by rose plot (N>35 cells, 3 repeats).

(B) Cells imaged as in (A) in gradient softened CDM; top right: rigidity across gradient softened CDM assessed by AFM before seeding cells (N=3; bars = SEM); bottom right: orientation of migration (locally softened matrix at 0°) shown by rose plot (N>35 cells, 3 repeats).

(C) A2780 cells on 2D FN-coated durotactic gradients imaged as in (A); top right: rigidity of 2D durotactic gradients assessed by AFM (N=3; bars = SEM); bottom right: orientation of migration with respect to the gradient shown by rose plot (N=58 cells, 3 repeats).

(D) Representative images from optical trap membrane tether experiments; white dotted line indicates separation of images.

(E) Paired trap force measurements at the front and rear of cells on durotactic gradients (left; N=29 cells, 3 repeats); or on uniform stiff gels (right; N=10, 3 repeats).

(F) Left, center: Cells migrating in CDM stained with Flipper-TR membrane tension probe; photon counts per pixel (left) and fluorescent lifetime imaging (FLIM) lifetime per pixel with a 16-color LUT (center); right: pairwise average Flipper-TR lifetime in manually identified membrane regions at the front and rear of cells (N=9 cells, 3 repeats; lower lifetime corresponds to lower membrane tension). Yellow arrows indicate direction of movement. $^{*}p < 0.05$; $^{**}p < 0.01$; $^{****}p < 0.0001$; ns, not significant. See also Figure S1 and Videos S1 and S2.

serial transmission EM (sTEM) and acquired stitched high-resolution transmission EM (TEM) images of serial sections (every 100 nm, 65 sections, 6.5 µm) to fully reconstruct migrating cells at the nanoscale. TEM demonstrated that caveolae were enriched at the membrane at the rear of the cells in 3D matrix (Figure 2H). Segmentation of caveolae and caveolae-associated

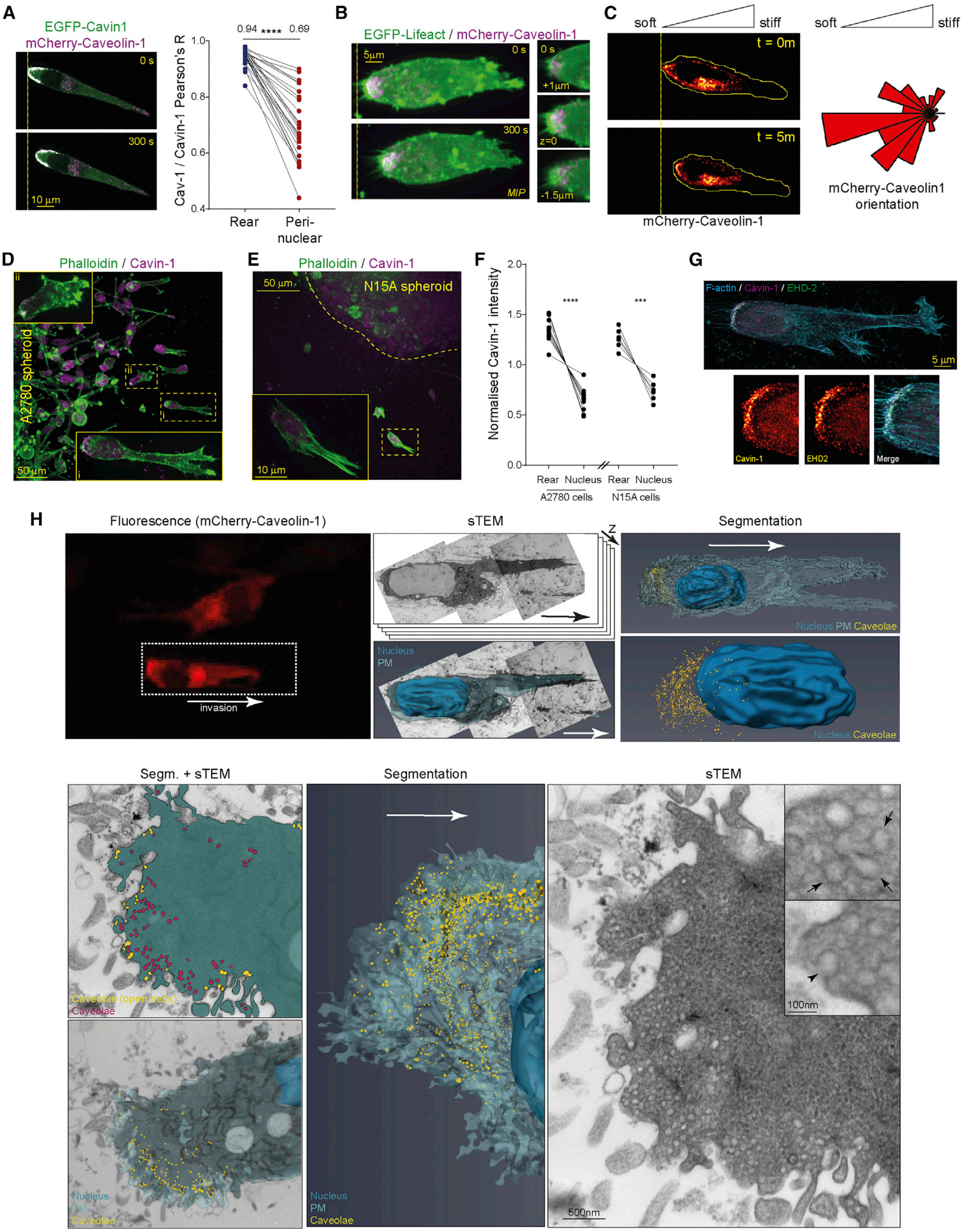

Figure 2. Low Membrane Tension Promotes Caveolae Formation at the Rear of Fast-Moving Cells in 3D and Durotactic Gradients

(A) A2780 cells expressing EGFP-cavin-1 and mCherry-caveolin-1 were imaged as in Figure 1A; pairwise Pearson's correlation coefficient for co-localization of EGFP-cavin-1 and mCherry-caveolin-1 in rear compared to peri-nuclear regions (N=28 cells, mean value above).

(legend continued on next page)

vesicles at the rear of the cell revealed numerous individual and multilobed structures, and interestingly, many of these possessed a clear "neck" connected to the plasma membrane (Ludwig et al., 2013). These data indicate that fast-moving cells in 3D matrix accumulate caveolae at the rear, which could act as a membrane reservoir to collect accumulating membrane.

Caveolae Respond to Low Membrane Tension to Form at the Cell Rear

Because caveolae formation is sensitive to membrane tension (Sinha et al., 2011), we used osmotic shock to acutely manipulate membrane tension (Pontes et al., 2017; Sinha et al., 2011). Introduction of 50% hypo-osmotic medium into the imaging chamber opposed caveolae formation at the rear of cells in 3D matrix and in durotactic gradients, while internal mCherry-caveolin-1 in the perinuclear region was relatively unchanged, and forward movement of the rear was suppressed (Figures 3A, 3B, and S3A–S3C; Video S5). Returning cells to isotonic medium was sufficient to allow re-establishment of caveolae and rear retraction (Figures 3A and 3B; Video S5). Live imaging of mCherry-caveolin-1 in cells transferred from hypo-osmotic to isotonic medium demonstrated a rapid increase in the average fluorescence intensity of caveolin-1 structures around the cell rear concomitant with re-establishment of rear retraction, while the average intensity of caveolin-1 structures in the rest of the cell remained relatively constant (Figure 3C). This indicates that caveolin-1 accumulation at the cell rear does not coincide with removal of caveolin-1 structures from other regions of the cell; rather, caveolin-1 accumulation is perhaps consistent with diffusion in the plasma membrane giving rise to higher average intensity structures. These data suggest caveolae are dynamic structures that can form spontaneously in response to low membrane tension at the rear rather than being trafficked from other regions of the cell.

In order to locally influence membrane tension in live cells, we expressed constitutively active (CA) ezrin mutant (ezrin T567D-GFP; relieves autoinhibition [Gautreau et al., 2000]) to promote membrane-cortex attachment and increase membrane tension (Ben-Aissa et al., 2011; Rouven Brückner et al., 2015) and took advantage of the finding that this protein accumulates specifically around the rear of cells in 3D matrix (Figure 3D). CA-ezrin significantly abrogated the rate of rear retraction, to a greater extent than wild-type ezrin (Figure 3D) and prevented cavin-1 accumulation at the cell rear (Figure 3E). Furthermore, this mutant opposed the membrane tension differential observed in cells moving on 2D durotactic gradients (Figure 3F). Taken together, these data show that caveolae respond to a local decrease in membrane tension to form and become stabilized at the cell rear and promote retraction of the migrating cell rear.

Caveolae Activate RhoA at the Cell Rear to Promote Retraction

Caveolin-1 has been implicated in the regulation of RhoGTPases, cell polarity, cell migration, and wound healing in the mouse dermis (Grande-García et al., 2007; Parton and del Pozo, 2013; del Pozo et al., 2005), but the function of caveolae at the rear of migrating cells or role in rear retraction is not known. In 3D matrix, knockdown of caveolin-1 with either of two independent siRNAs significantly reduced the speed of migration over long timelapses and increased cell length (Figures S4A–S4D). Caveolin-1 knockdown had little impact on the ability of cells to generate and extend protrusions but reduced the distance that the cell rear translocated (Figures 4A, S4E, and S4F), demonstrating a clear retraction defect in 3D migration. EHD2 knockdown resulted in loss of caveolin-1 localization at the rear of cells moving in 3D matrix (Figure S4G) and decreased speed of migration, and cells were elongated and showed defective cell rear translocation (Figures S4G–S4H), supporting a role for caveolae in rear retraction.

Because caveolae have previously been implicated in the regulation of RhoGTPases (Parton and del Pozo, 2013), we hypothesized that caveolae could play a role in the activation of RhoA to organize the actin cytoskeleton and mediate rear retraction. Using a location biosensor to report on active RhoA localization (GFP-AHPH [Piekny and Glotzer, 2008; Priya et al., 2015]), we observed a punctate distribution of active RhoA concentrated around the retracting rear of cells moving in 3D matrix and in durotaxis (Figures S4I and S4J). Active RhoA and caveolin-1 co-distributed at the rear of cells moving in 3D matrix and durotactic gradients (Figures 4B and S4K), and high-resolution imaging revealed partial co-localization (Figure 4C), suggesting that caveolae and active RhoA localize in close proximity at the rear of migrating cells. Next, we used a RhoA fluorescence resonance energy transfer (FRET) biosensor to show that RhoA activity, as expected, was elevated at the rear of cells migrating in 3D matrix, but knockdown of caveolin-1 significantly abrogated RhoA activation at the cell rear (Figure 4D). Increasing membrane tension with osmotic shock (which impairs caveolae formation; Figures 3A–3C) similarly impaired the activation of RhoA (Figures 4E and S4L), suggesting that caveolae sense and respond to low

(B) A2780 cells expressing Lifeact-EGFP and mCherry-caveolin-1 imaged as (A), maximum intensity projection (MIP) shown for whole cell (left), individual Z-slices for rear regions (right).

(C) Left: A2780 cells expressing mCherry-caveolin-1 seeded onto 2D durotactic gradients and imaged as in (A), yellow line indicates outline of the cell; right: orientation of caveolae with respect to the gradient shown by rose plot (N=58 cells, 3 repeats).

(D and E) A2780 (D) or N15A (E) cell spheroids within collagen and fibronectin hydrogels stained for F-actin (phalloidin) and Cavin-1. Spheroid edge denoted by yellow dotted line; inset: higher-resolution image of the highlighted individual invading cells. MIPs of Z-stacks shown.

(F) Pairwise normalized rear and nuclear Cavin-1 intensity in individual invading A2780 and N15 cells (N>8 cells analyzed, 3 repeats).

(G) A2780 cell seeded in 3D CDM and fixed and stained for F-actin, cavin-1, and EHD2; whole cell and separately captured zoomed rear MIPs of confocal stacks shown.

(H) Serial transmitted electron microscopy (sTEM, 100-nm sections) of A2780 cell expressing mCherry-Caveolin-1 in CDM. Manual segmentation of the nucleus, the plasma membrane and caveolae are displayed as a 3D model over the entire cell. Both caveolae connected to the plasma membrane (yellow) and caveolae-associated vesicles (pink) were segmented (segm. + sTEM image). Both structures were merged in yellow in the overall model. An individual sTEM image is presented. Insets show higher-resolution images of multilobed (arrows) or membrane-attached (arrowhead) caveolae (different z-section of the same cells). Scale bar, 500 nm (inset: 100 nm). ***p < 0.001; ****p < 0.0001. See also Figure S2 and Videos S3 and S4.

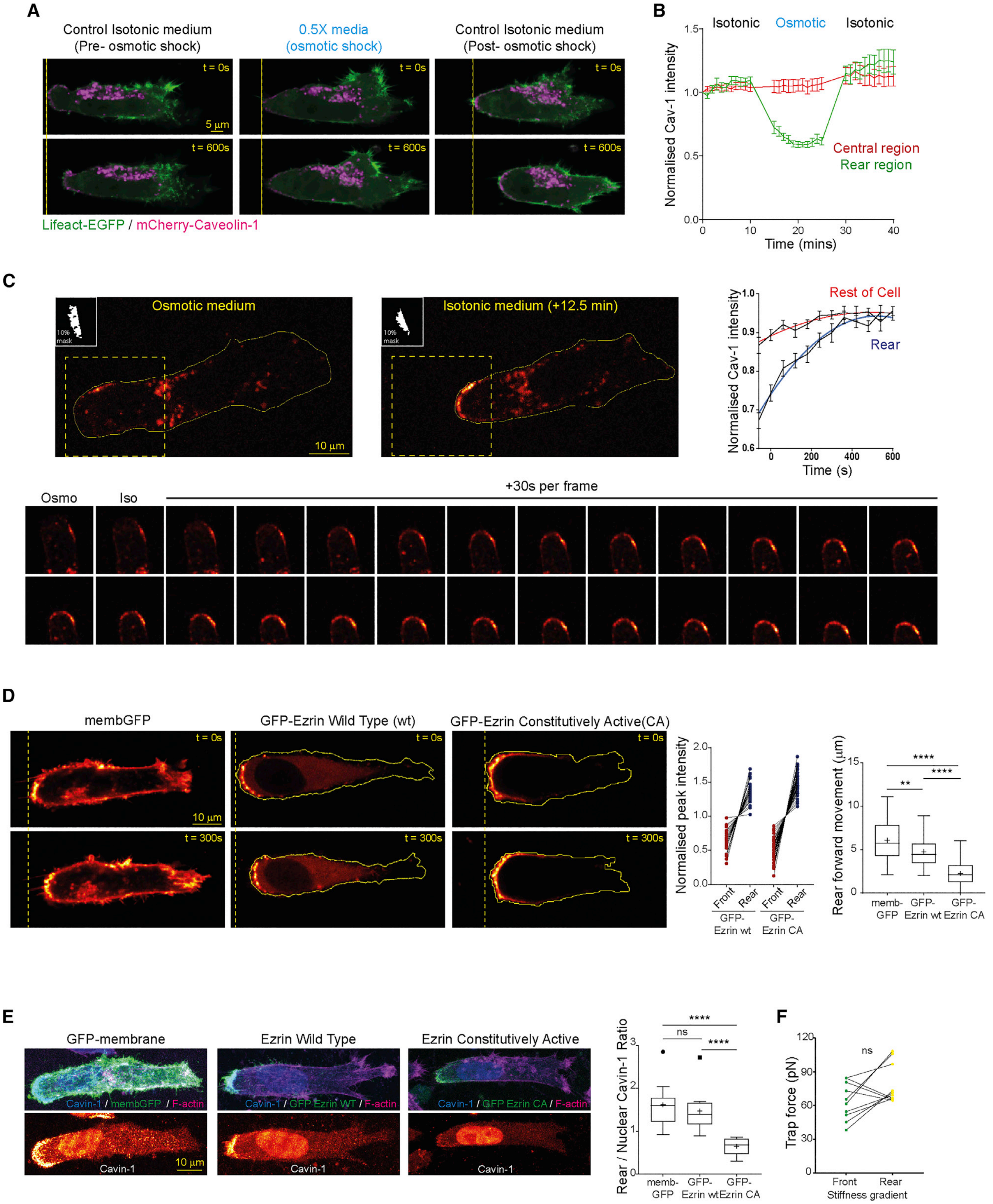

Figure 3. Increasing Membrane Tension Abrogates Rear Caveolae Formation and Forward Translocation

(A) A2780 cell in 3D CDM imaged before and during hypo-osmotic shock and after recovery in isotonic media.

(B) Rear mCherry-caveolin-1 intensity before and during osmotic shock, and after recovery in isotonic medium. N=12 cells, 3 repeats, bars = SEM.

(legend continued on next page)

membrane tension at the cell rear and activate RhoA in this subcellular region.

RhoA Coordinates Rear Retraction by Organizing F-Actin at the Cell Rear

RhoA plays a key role in regulating actomyosin contractility and retraction of the rear in migrating cells in 2D (Ridley et al., 2003), and we found that RhoA knockdown cells showed a clear defect in rear retraction in 3D migration (Jacquemet et al., 2013; Figure S4M). Having identified a rear retraction defect associated with a caveolae-RhoA pathway, we analyzed how this pathway influenced the organization of actin in cells migrating in 3D. Knockdown of caveolin-1 had very little effect on the intensity of F-actin toward the front of cells moving in 3D matrix, but strikingly decreased F-actin intensity at the rear (Figure 4F). Similarly, Y27632, an inhibitor of ROCK and PKN RhoA effector kinases (Bain et al., 2007), decreased F-actin intensity in the rear portion of the cell (Figure S4O). Knockdown of RhoA and ROCK1 and PKN2 (PRK2 but not ROCK2, PKN1, or PKN3) selectively abrogated migration speed, F-actin intensity, and organization at the cell rear (Figures 4G–4J and S4N–S4Q). Strikingly, high-resolution imaging revealed that F-actin was organized into cables oriented along the front-rear axis at the cell rear, which were lost upon ROCK1 and PKN2 depletion (Figures 4H and S4Q). Inhibition of Rho-effector kinases or knockdown of caveolin-1 significantly abrogated rear retraction independently but had no additive effect (Figure 4K), suggesting that caveolin-1 and RhoA effector kinases form part of the same regulatory pathway. These data indicate that caveolae activate RhoA-ROCK1 and RhoA-PKN2 signaling modules which organize F-actin specifically at the cell rear and promote actin organization and contractility.

Ect2 Is Recruited by Caveolae to Activate RhoA and Promote Rear Retraction

Caveolae have been linked to RhoA activity, at least in part by preventing RhoA inactivation by p190RhoGAP (Grande-García et al., 2007). However, guanidine nucleotide exchange factors (GEFs) that specifically activate RhoA in this context are not known. By using a GFP-trap and mass spectrometry approach to identify GFP-RhoA binding proteins, we uncovered six candidates potentially involved in RhoGTPase activation, the GEFs Ect2, Vav2, ARHGEF2 (GEF-H1), PLEKHG5, DOCK1, and its co-factor ELMO2 (Figure S5A; data not shown). Knockdown of these candidates revealed that Ect2, Vav2, ARHGEF2, and PLEKHG5 played a role in motility in 3D matrix (Figures S5A–S5C). While knockdown of Ect2 and PLEKHG5 resulted in an elongated morphology consistent with a defect in retraction (Figures S5B–S5D), knockdown of Ect2 alone caused a clear defect in rear retraction in cells moving on 3D matrix (Figure 5A). Moreover, knockdown of Ect2, but not Vav2 or PLEKHG5, significantly reduced the activation of RhoA at the rear of cells moving in 3D matrix (Figure 5B), suggesting that Ect2 acts as the main GEF that activates RhoA to mediate rear retraction.

Ect2 is targeted to the membrane after nuclear envelope breakdown to activate RhoA and execute cytokinesis (Kotýnková et al., 2016; Matthews et al., 2012; Su et al., 2011), and although it is localized predominantly to the nucleus in interphase cells, Ect2 activates RhoA at cell-cell junctions to promote their stability (Priya et al., 2015; Ratheesh et al., 2012). In fast-moving cells in 3D matrix, Ect2 was nucleocytoplasmic and accumulated at the cell rear where it co-localized with cavin-1 (Figure 5C). Knockdown of caveolin-1, however, suppressed the accumulation of Ect2 at the cell rear without markedly influencing the nucleocytoplasmic balance (Figure 5D). This suggests that Ect2 is specifically recruited to the cell rear by caveolae to activate RhoA and promote rear retraction.

Computational Models Describe Persistent Movement in Mechanically Guided Cell Migration

In order to gain further insight into the dynamic signaling and mechanical events leading to rear retraction of cells within durotactic gradients and upon strain stiffening of 3D-ECM, we built a mathematical model based on ordinary differential equations to describe the mechanochemical signaling that controls rear retraction (reactions/nodes and parameters described in STAR Methods). Because we observe caveolae formation in durotactic migration, polarized substrate stiffness is the only external input, transduced from cell-matrix adhesion complexes at the cell front to initiate forward translocation of the cell and establish a front-rear membrane tension differential (Figures 6A and 6B). Our model recapitulates signaling occurring at the cell rear in response to mechanical inputs, leading to F-actin organization and phosphorylation of MLC via ROCK1/PKN2 (Figures 6A and 6B). We explicitly modeled initial polarized substrate stiffness (set to 100%) to mimic the strain-stiffened state of matrix in front of cells moving in 3D matrix and the substrate encountered by cells on 2D rigidity gradients, while all other initial (non-inhibitory) variables were set as zero. Simulations of this non-homogenous modeled domain showed the output "rear retraction" (conceptualized as directional rear movement, such that a high value corresponds to persistent, fast movement, and a low value corresponds to slow migration and/or random, non-persistent directionality) to rapidly increase to a high steady state, while without the external polarized substrate input (uniform throughout), rear retraction levels were negligible (Figure 6C). Sensitivity analysis (Figure S6A) revealed critical model parameters, and halving or doubling critical parameters had little effect on modeled outputs (Figure S6B), suggesting that the model

(C) Top left: cells as in (A) imaged every 30 s immediately after media change; top right: average normalized mCherry-caveolin-1 intensity in 10% rear mask (white inset in images) and rest of cell regions over time following osmotic to isotonic switch (N=17 cells, 3 repeats, bars = SEM, fitted quadratic curves showed in red and blue); bottom: montage of zoomed, 90° rotated rear region (highlighted by yellow box) imaged every 30 s post- osmotic-isotonic switch showing mCherry-caveolin-1 accumulation.

(D) Left: A2780 cell in CDM expressing GFP-membrane (left), Ezrin wild type (WT, center) or Ezrin constitutively active (CA, right); center: pairwise normalized peak Ezrin intensity within front and rear regions (N>32 cells/condition, 3 repeats); right: average rear forward movement over 5 min (N > 39, 3 repeats).

(E) Left: cells in CDM stained for F-actin (phalloidin) and caveolin-1, MIPs shown; center: rear/nuclear peak caveolin-1 intensity ratio (N>11 cells/condition, 3 repeats); right: paired trap force measurements at the front and rear of cells expressing CFP Ezrin-CA on durotactic gradients (N=10 cells, 3 repeats).

**p < 0.01; ****p < 0.0001; ns, not significant. See also Figure S3 and Video S5.

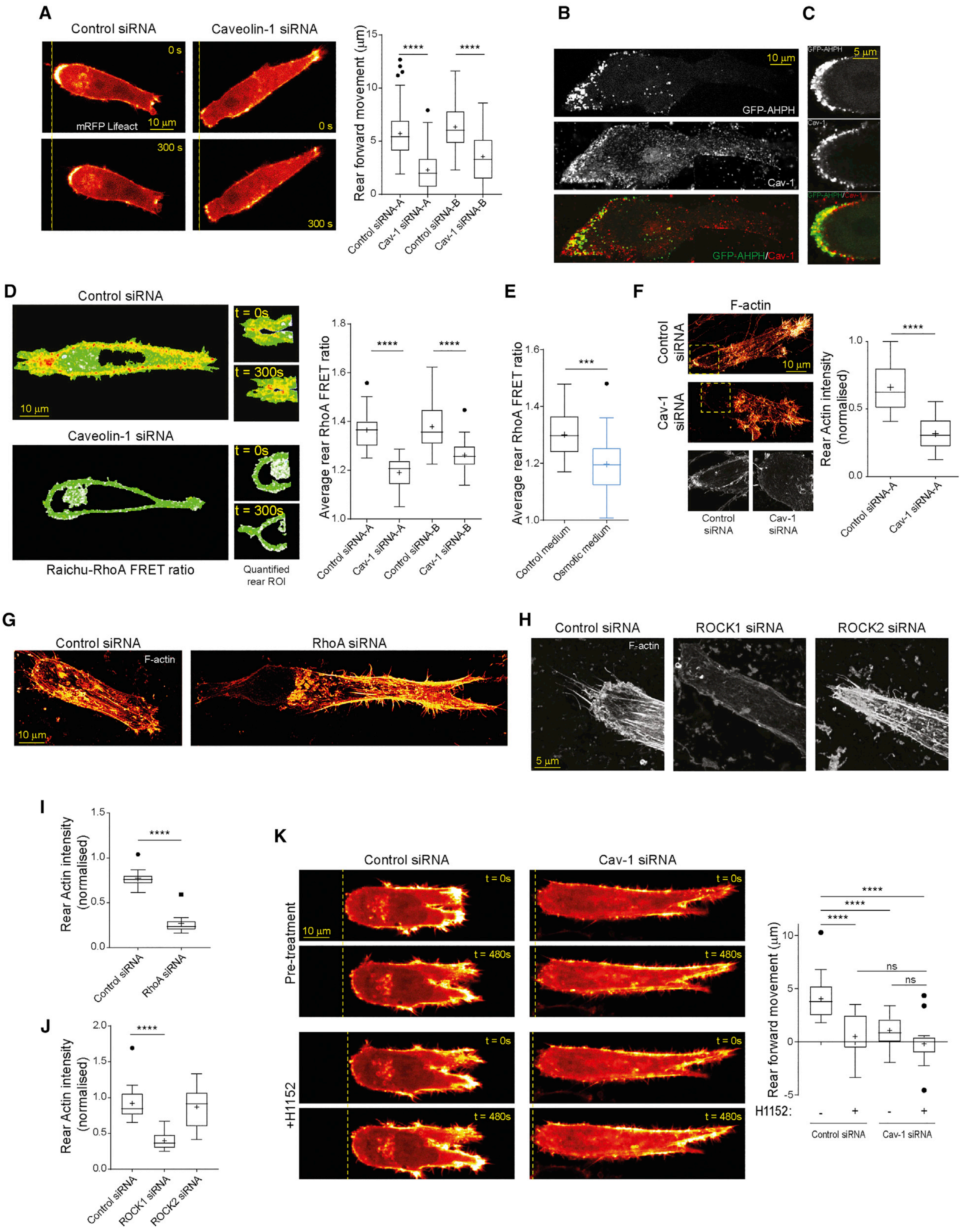

Figure 4. Caveolae Activate RhoA at the Cell Rear to Control Contractility and F-actin Architecture

(A) Lifeact-mRFP expressing, control or caveolin-1 knockdown A2780 cells in 3D CDM and forward movement of cells over 5 min (N>74 cells/condition, 3 repeats).

(B) GFP-AHPH expressing A2780 cells seeded in CDM, stained for endogenous caveolin-1, MIP shown.

(legend continued on next page)

and its overall network topology was robust. Interestingly, simulation of a transition from polarized to uniform substrate indicated that the rear retraction output was near identical to within a gradient, while transition from uniform to polarized substrate simulations indicate that the rear would begin retracting accordingly after the transition (Figure 6C). This suggests that once a rear begins to rapidly retract, it can continue to do so independently of the external polarization conditions. This may reflect the ability of cells to move persistently in the disordered 3D matrix and suggests that rear retraction could act as a directional memory to conserve persistent migration in complex matrix.

Simulations of this model were in close agreement with our experimental findings: independent depletion of caveolae (knockdown of caveolin-1 and EHD2) or RhoA severely reduced F-actin alignment and rear retraction "speed" (Figure 6D) *in silico*. We could also mimic the reversible osmotic shock protocol, whereby increasing the transition from low to high membrane tension reduced caveolae formation and halted rear retraction, while returning the membrane tension to a decreased state rapidly restored rear caveolae accumulation and efficient retraction (Figure 6E). This gave confidence in the topology and structure of our model and suggested that it could serve as a predictive tool.

Positive Feedback Reinforcement of RhoA-Mediated Contractility

We theorized that a gradient of stiffness, transduced by actomyosin contractility from integrin adhesion complexes, would decrease rear membrane tension and promote formation of caveolae. Because caveolae formation at the cell rear promotes RhoA activation, we predicted a positive feedback loop between RhoA-mediated contractility within the rear domain and caveolae formation via a further decrease in membrane tension (Figures 6A and 6B). Simulations showed that this feedback loop was a plausible feature of the model and predicted that it could be broken (and caveolae formation arrested) by inhibition of Rho-effector kinases (simulated as an increase in both ROCK1 and PKN2 inactivation rates, *in silico* Y27632 treatment; Figure 6F). Similarly, increasing F-actin turnover, either globally or locally, was predicted to prevent further formation of caveolae and to halt forward movement of the rear (Figures 6G and 6H).

In order to experimentally verify positive feedback between RhoA signaling and caveolae formation, we first inhibited Rho-effector kinases. mCherry-caveolin-1 was rapidly redistributed from the rear of cells migrating in 3D matrix within 10 min (Figures 7A and 7B; Video S6), suggesting that signaling downstream of RhoA through ROCK1/PKN2 is required to maintain positive feedback. Similarly, RhoA knockdown cells failed to recruit mCherry-caveolin-1 to the cell rear in 3D matrix (Figure S7A–S7B). Knockdown of Ect2, RhoA, or ROCK1 suppressed the recruitment of endogenous caveolin-1/cavin-1 to the cell rear in 3D matrix (Figures 7C–7E, S7C, and S7D), indicating that RhoA signaling is required to form caveolae at the retracting rear. Furthermore, membrane tension at the rear of cells moving in 3D matrix was increased by inhibition of Rho-effector kinases (Figure 7G), suggesting that maintenance of low membrane tension requires the RhoA signaling cascade.

Cytochalasin D (CytoD), an agent that can both inhibit actin polymerization and promote depolymerization (Casella et al., 1981), was found to disrupt F-actin and rear caveolae in cells in 3D matrix (Figure S7E). In order to more locally influence F-actin turnover, we used a caged form of CytoD (Nvoc-CytoD [Latorre et al., 2018]; Figure S7F) and analyzed the effect of acute intervention on the formation of caveolae at the rear of cells actively migrating in 3D matrix. Initiating uncaging to release the active compound led to a reduction in F-actin intensity at the rear and the concomitant redistribution of mCherry-caveolin-1 (Figures 7H and S7G; Video S7). These data confirm that rear retraction is a dynamic process underpinned by a positive feedback loop centered on the formation of caveolae to amplify RhoA activity at the rear of migrating cells and effect rear retraction.

DISCUSSION

Rear retraction in migrating cells requires RhoA-mediated signaling to the contractile actin cytoskeleton (Ridley et al., 2003). Our study reveals how membrane tension acts as a key mechanical feature of cells moving in gradients of rigidity, to allow formation of caveolae, which in turn recruit Ect2 to coordinate activation of RhoA and the contractile cytoskeleton.

In 2D migration, some mechanistic detail has emerged revealing how RhoA is regulated and how NMII isoforms organize front-rear polarity (Raab et al., 2012; Sastry et al., 2006; Vicente-Manzanares et al., 2009). RhoA activity has also been implicated in driving rearward cortical actin and membrane flow to control the migration of amoeboid cells that lack strong cell-matrix interactions (Neill et al., 2018; Paluch et al., 2016). Our data demonstrate that in 3D matrix, rapid retraction is mediated by caveolae, which promote contractility signaling by

(C) Cell as in (B) imaged at a high resolution, Pearson correlation 0.52; Spearman's rank 0.65.
(D) Ratiometric FRET imaging of control or caveolin-1 knockdown A2780 cells expressing Raichu-RhoA in CDM. Average rear RhoA FRET ratio in control/caveolin-1 knockdown cells (N>36 cells/condition, 3 repeats).
(E) Average rear RhoA FRET ratio of in isotonic versus osmotic shock (N>27 cells/condition, 3 repeats).
(F) Control or caveolin-1 knockdown A2780 cells in CDM, fixed and stained with SiR-actin (MIPs shown); ratio of F-actin intensity of the rear to front of cell (N>17 cells/condition, 3 repeats).
(G) Control or RhoA knockdown A2780 cells in CDM as in (F).
(H) Control, ROCK1, or ROCK2 knockdown cells in CDM as in (F).
(I) Rear actin intensity of control and RhoA knockdown cells (N=10 cells/condition, 3 repeats).
(J) Rear actin intensity of control, ROCK1, and ROCK2 knockdown cells (N>17 cells/condition, 3 repeats).
(K) Left: Control (left) and caveolin-1 knockdown (right) Emerald-Lifeact expressing A2780 cells seeded in CDM imaged prior to (top) and post- (bottom) treatment with H1152; right: Rear movement of control and caveolin-1 knockdown cells over 5 min pre- and post-H1152 treatment (N=21 cells/condition, 3 repeats).
***$p < 0.001$; ****$p < 0.0001$. See also Figure S4.

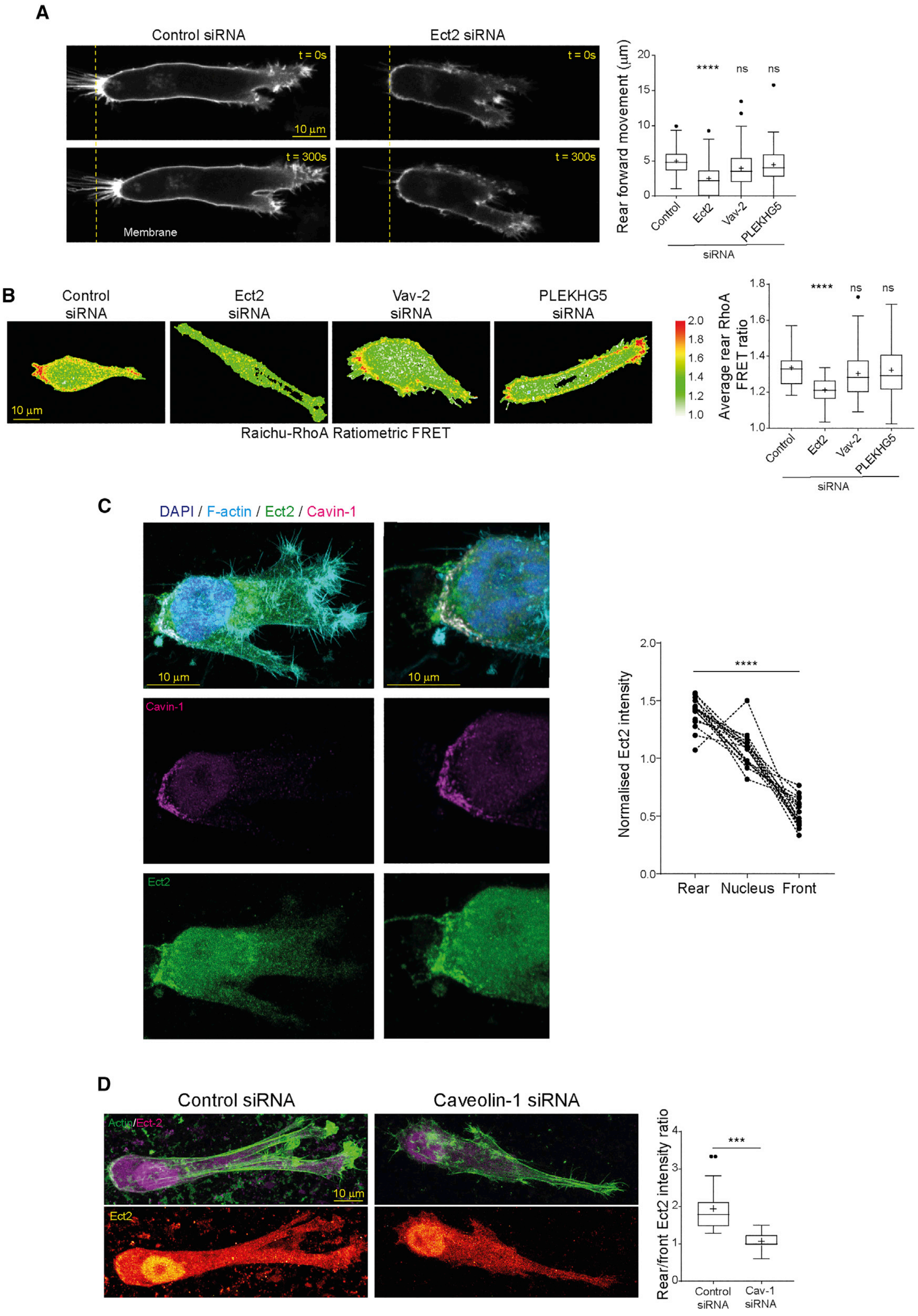

Figure 5. Ect2 Is Recruited by Caveolae to Activate RhoA

(A) Control (left) and Ect2 knockdown (center) A2780 cells expressing membrane-targeted Raichu-RhoA in 3D CDM; right: average forward rear movement over 5 min of control, Ect2, Vav2, and PLEKHG5 knockdown cells (N>46 cells/condition, 3 repeats).

(legend continued on next page)

recruiting the RhoAGEF Ect2 to activate RhoA and its effectors ROCK1 and PKN2. Positive feedback can then be established by actomyosin contractility to promote further reduction in membrane tension, caveolae formation, and hyperactivation of RhoA. This mechanism explains rapid retraction of the migrating cell rear, allowing single cells to move fast and persistently in 3D matrix and on gradients of rigidity.

Cells in 3D matrix can strain stiffen their local environment (Van Helvert and Friedl, 2016) to create or amplify any differences in rigidity that might exist. A gradient of rigidity could generate a membrane tension differential through adhesion complexes at the front of polarized cells, which would encounter higher resistive force resulting in net forward movement of the contractile actin cytoskeleton and lower membrane tension where membrane-cortex interactions are weakened. Using Flipper-TR, we show that the in-plane membrane tension is reduced at the rear of cells moving in 3D (Figure 1F). Optical-trap-based tether pulling shows a very similar trend in cells on 2D rigidity gradients, where this approach measures the product of both in-plane membrane tension and membrane-cytoskeleton connections. Interestingly, increasing membrane-cytoskeleton attachments using CA-ezrin has a significant effect on caveolae recruitment and rear retraction (Figure 2D and 2E). This suggests that in the context of caveolae formation, the in-plane membrane tension and membrane-cytoskeletal linkage could be linked.

While caveolae have been shown to localize to the cell rear in 2D (Beardsley et al., 2005; Lentini et al., 2008; Parat et al., 2003; Parton and del Pozo, 2013; Urra et al., 2012), their function there has been poorly understood. Our data demonstrate that caveolae act to recruit a RhoA GEF, Ect2, which activates RhoA to coordinate the retraction phase of migration. Polarization of membrane tension (and caveolae) is less apparent on uniformly stiff substrates, but it is possible that caveolae play a similar regulatory role in some cell types during the retraction phase of the migration cycle on these surfaces.

Caveolae are known to function as endocytic carriers (Parton and del Pozo, 2013), but in our study, the role of caveolae appears to be related to their described capacity (Parton and del Pozo, 2013) to form where the plasma membrane is under low tension. Indeed, acute temporal or spatial manipulation of membrane tension (using osmotic shock or rear-localized CA-ezrin) abrogates caveolae formation and rear retraction (Figure 3). sTEM demonstrated the accumulation of caveolae around the retracting rear membrane, including multilobed structures and caveolae with clear membrane-associated neck structures (Figure 2H). In support of this EHD2, which stabilizes the neck of caveolae, is required for rear retraction and localizes to caveolae at the cell rear (Figures 2G and S4G). However, in our sTEM images, many caveolae are present close to the retracting rear plasma membrane with no apparent neck (Figure 2H), and it is not clear whether this is a limitation of the z-sectioning approach, or if these represent internal caveolae structures. Given that some caveolin-1 puncta appear to internalize after breaking the positive feedback loop that establishes rapid retraction (Figure 7), it remains possible that caveolae internalization can occur from the cell rear and could act as an important step particularly when migrating cells change direction or arrest.

Caveolin-1 has been shown to promote RhoA activity by suppressing Src and the RhoA inactivator p190RhoGAP (Grande-García et al., 2007) and is implicated in the generation of actomyosin contractility in cancer-associated fibroblasts, activating RhoA to promote reorganization of stromal matrix (Goetz et al., 2011). In addition, it is now clear that caveolae play a role in protecting against mechanical strain in numerous cell systems by buffering against increasing membrane tension (Cheng et al., 2015; Lim et al., 2017; Parton and del Pozo, 2013; Sinha et al., 2011). Our study extends these findings, suggesting that when cells generate directional traction force in a durotactic gradient, contractility within the actin cytoskeleton along the front-rear axis transmits a signal through decreased rear membrane tension that is sensed by caveolae to promote RhoA activation via Ect2, which could itself be directed to the membrane by interaction with lipids, as is the case prior to cytokinesis (Kotýnková et al., 2016; Su et al., 2011).

Downstream of RhoA, the effector kinases ROCK1 and PKN2 each play key roles in actin organization and rear retraction (Figures 4 and S4). A specific requirement for PKN2 in cell migration has previously been shown in cancer cells and *in vivo* (Lachmann et al., 2011; Quétier et al., 2016), suggesting that RhoA effector kinases synergize to control F-actin organization and contractility in rear retraction. Surprisingly, the effects of caveolae disruption and RhoA signaling intervention are confined to a large region at the back of migrating cells, suggesting that this signal-regulating F-actin is compartmentalized to the cell rear. The nucleus is limiting in migration through complex environments and can even rupture under force exerted on it (Denais et al., 2016; Irianto et al., 2017; Raab et al., 2016; Wolf et al., 2013), and it is therefore possible that this large organelle acts to partition cytoskeletal regulation.

Cell migration is a widely studied phenomenon in 2D yet still is relatively poorly characterized in more physiological 3D environments such as the interstitial matrix encountered by metastatic cancer cells and fibroblasts in wounding (Caswell and Zech, 2018). Here, we have identified a mechanism that controls the least well-understood phase of the migration cycle, rear retraction. By establishing a positive feedback loop resulting in hyperactivation of RhoA, membrane tension could act as a mechanical signal that allows cells to move rapidly with high directional persistence into fibrotic areas (e.g., wounds) or promote cancer invasion.

(B) Ratiometric FRET imaging of control, Ect2, Vav2, and PLEKHG5 knockdown cells; average rear RhoA FRET ratio in control, Ect2, Vav2, and PLEKHG5 knockdown cells (N>32 cells/condition, 3 repeats).

(C) Endogenous Ect2 and cavin-1 in A2780 cells in 3D-CDM; MIPs of whole cell and separately captured zoomed rear region. Right: peak Ect2 staining intensity in rear, nuclear, and front regions (N=17 cells, 3 repeats).

(D) Control (left) and caveolin-1 knockdown cells (right) in CDM stained for F-actin (phalloidin) and Ect2, MIPs shown; Rear peak/front peak Ect2 intensity levels (N>20 cells/condition, 3 repeats).

***$p < 0.001$; ****$p < 0.0001$; ns, not significant. See also Figure S5.

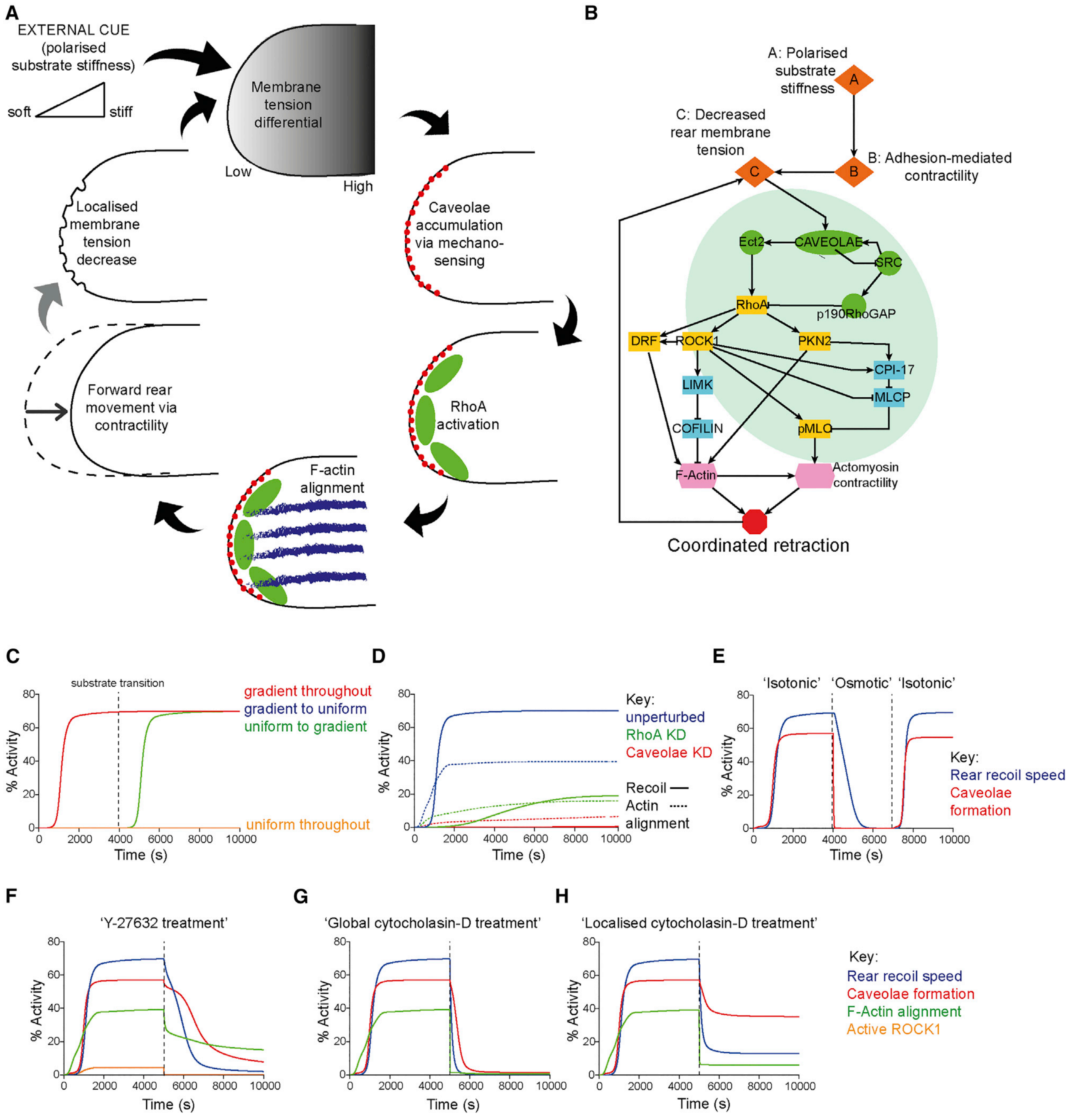

Figure 6. Mathematical Modeling Implicates a Positive-Feedback-Based Mechanism of Rear Retraction

(A) Schematic summary of key rear retraction events for migrating cells in 3D or durotaxis.

(B) Wiring diagram of mechanochemical signaling interactions that lead to coordinated retraction, upon which the ordinary differential equations (ODE) model and simulations are based.

(C) Simulations output of rear recoil speed (% activity) against time with polarized substrate stiffness remaining at 100% (red) or at 0% throughout the time course (yellow), or transitioning from 100% to 0% (gradient to uniform, blue, hidden by red line) or 0% to 100% (uniform to gradient, green) at time t = 4,000 s.

(D) Simulation outputs for rear recoil speed and F-actin alignment for unperturbed, RhoA, and caveolae knockdown initial conditions.

(E) Simulation of reversible osmotic shock, where membrane tension is increased at t = 4,000 s and returned to basal level at t = 7,000 s; rear recoil speed and caveolae formation shown.

(F) Predictive simulation of Y-27632 treatment effect on caveolae dynamics and rear recoil speed, where at t = 5,000 s ROCK1/PKN-2 inactivation rates are greatly increased.

(G) Predictive simulation of "global" cytochalasin-D treatment on caveolae dynamics/rear recoil speed, where at t = 5,000 s, F-actin turnover rate is greatly increased.

(H) As in G but for "local" cytochalasin-D, where at t = 5,000 s, F-actin turnover rate is moderately increased.

See also Figure S6.

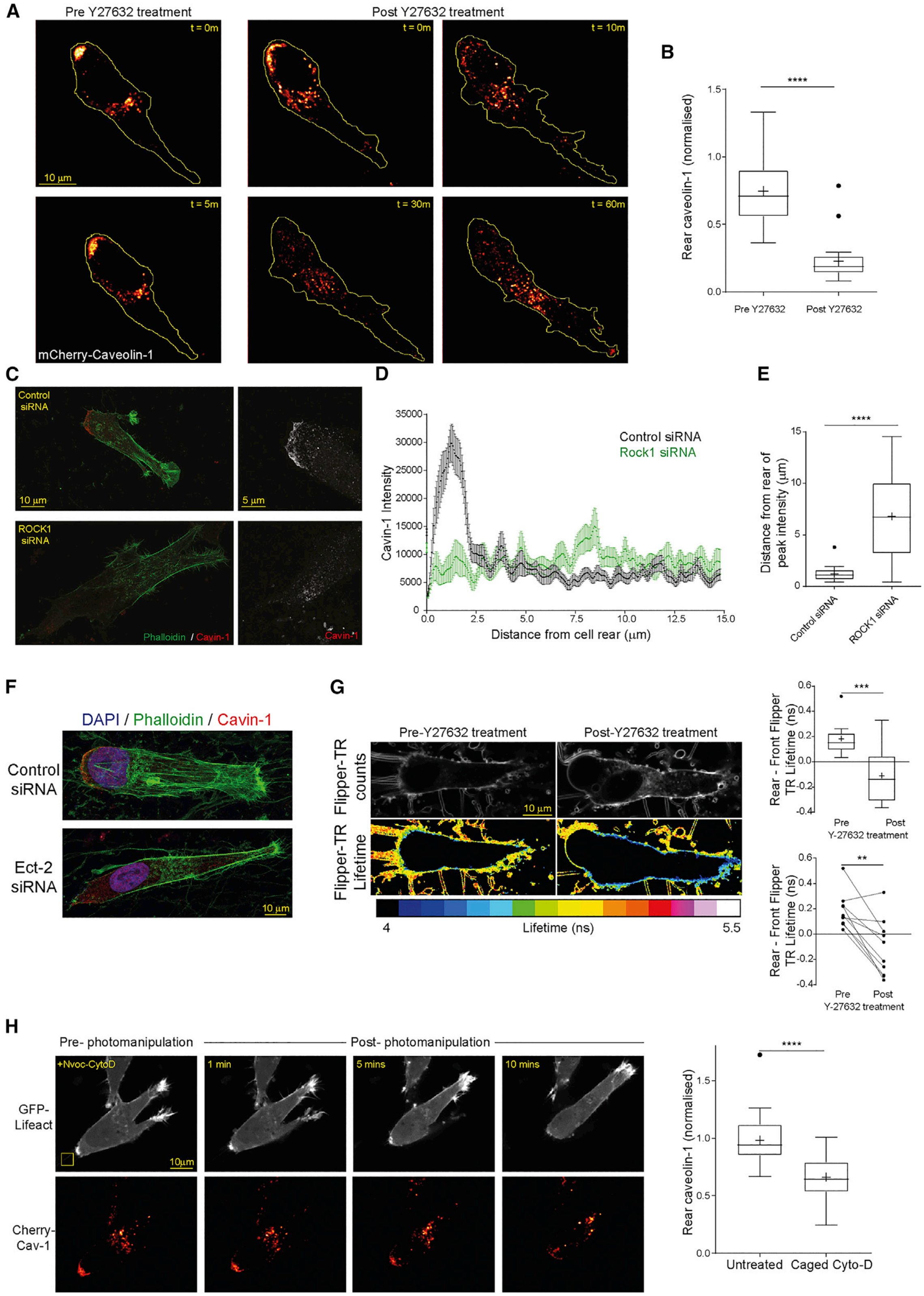

Figure 7. F-Actin Stability and Contractility Maintain Caveolar Rear Localization in Migrating cells

(A) mCherry-caveolin-1 expressing A2780 cells in 3D CDM imaged before (left panels) and after treatment with Y27632.

(B) Rear caveolin-1 intensity of cells as in (A) (N=23 cells, 3 repeats).

(legend continued on next page)

STAR★METHODS

Detailed methods are provided in the online version of this paper and include the following:

- KEY RESOURCES TABLE
- LEAD CONTACT AND MATERIALS AVAILABILITY
- EXPERIMENTAL MODEL AND SUBJECT DETAILS
  - Cell Culture and Transient Transfection
- METHODS DETAILS
  - Reagents Usage Conditions
  - SDS-PAGE and Quantitative Western Blotting
  - CDM Production
  - Generation of Polyacrylamide Stiffness Gradients
  - Atomic Force Microscopy of 3D-CDM
  - Atomic Force Microscopy of 2D Gradients
  - Membrane Tension Measurements by Optical Trap
  - Live Fluorescence Imaging
  - Membrane Tension Measurements by FLIM
  - Long Term Cell Migration Assay
  - Fixed Cell High Resolution Imaging
  - 3D Spheroid Invasion Assay
  - Correlative Light and Electron Microscopy
  - Correlative Light and SBFSEM
  - Generation of Rose Plots for Directional Analysis
  - Cell Region Based Quantification
  - Quantification of Localized Adhesion Numbers
  - Reversible Live Hypo-osmotic Shock
  - Hypo-osmotic Shock of Fixed Cells and FRET
  - Live Y27632 and H1152 Treatment
  - Global and Caged Cytochalasin-D Treatment
  - Model Construction
  - ODEs Formulation
  - Initial Conditions
  - Parameter Values
  - Model Simulation and Perturbation
  - Parameter Sensitivity Analysis
- QUANTIFICATION AND STATISTICAL ANALYSIS
- DATA AND CODE AVAILABILITY

SUPPLEMENTAL INFORMATION

Supplemental Information can be found online at https://doi.org/10.1016/j.devcel.2019.09.006.

ACKNOWLEDGMENTS

We are grateful to D. Bradley, E. Hinde, P. March, N. Hodson, E. Zindy, S. Brett, F. Chunara, and Y. Zhang for technical support. The Bioimaging Facility microscopes were purchased with grants from BBSRC, Wellcome, and the University of Manchester Strategic Fund (UK). The Liverpool Centre for Cell Imaging (CCI) provided access to equipment (BBSRC grant BB/M012441/1). P.T.C. is supported by the MRC (MR/R009376/1), J.S. is supported by the BBSRC (BB/L024551/1), and the Wellcome Trust Centre for Cell-Matrix Research is funded by grant 203128/Z/16/Z. CLEM experiments were funded by ARC (Programme labellisé to J.G.G.) and by institutional funds from INSERM and University of Strasbourg (France). I.B. is supported by ARC (Programme labellisé to J.G.G.), and we are grateful to Luc Mercier for help with the CLEM approach and Cathy Royer (INCI, Strasbourg) for access to TEM.

AUTHOR CONTRIBUTIONS

Conceptualization, J.H.R.H., H.d.B., A.C., N.G., C.L., J.G.G., E.K.P., J.-M.S., and P.T.C.; Methodology, J.H.R.H., H.d.B., I.B., T.W., R.V.N., O.D., J.S., A.d.C., T.S., T.Z., J.G.G., E.K.P., J.-M.S., and P.T.C.; Validation, J.H.R.H., H.d.B., I.B., T.W., V.S., T.S., J.G.G., and P.T.C.; Formal Analysis, J.H.R.H., H.d.B., I.B., T.W., V.S., T.S., and P.T.C.; Investigation, J.H.R.H., H.d.B., I.B., T.W., V.S., T.S., and P.T.C.; Resources, J.H.R.H., H.d.B., I.B., T.W., A.C., R.V.N., C.L., A.d.C., T.S., T.Z., J.G.G., E.W.K., and P.T.C.; Data Curation, J.H.R.H., H.d.B., T.S., J.G.G., E.K.P., and P.T.C.; Writing, all authors; Visualization, J.H.R.H., H.d.B., I.B., V.S., T.S., J.G.G., and P.T.C.; Supervision: J.H.R.H., J.G.G., E.K.P., J.-M.S., and P.T.C.

DECLARATION OF INTERESTS

The authors declare no competing interests.

Received: September 5, 2018
Revised: July 2, 2019
Accepted: September 10, 2019
Published: October 10, 2019

REFERENCES

Bain, J., Plater, L., Elliott, M., Shpiro, N., Hastie, C.J., Mclauchlan, H., Klevernic, I., Arthur, J.S.C., Alessi, D.R., and Cohen, P. (2007). The selectivity of protein kinase inhibitors: a further update. Biochem. J. *408*, 297–315.

Beardsley, A., Fang, K., Mertz, H., Castranova, V., Friend, S., and Liu, J. (2005). Loss of caveolin-1 polarity impedes endothelial cell polarization and directional movement. J. Biol. Chem. *280*, 3541–3547.

Ben-Aissa, K., Liu, Y., Park, C., Patino-Lopez, G., Shaw, S., Hao, J.-J., von Andrian, U.H., Nambiar, R., Belkina, N.V., Qi, H., et al. (2011). Constitutively active ezrin increases membrane tension, slows migration, and impedes endothelial transmigration of lymphocytes in vivo in mice. Blood *119*, 445–453.

Breitsprecher, D., and Goode, B.L. (2013). Formins at a glance. J. Cell Sci. *126*, 1–7.

Bretscher, M.S. (1984). Endocytosis: relation to capping and cell locomotion. Science *224*, 681–686.

Cardona, A., Saalfeld, S., Schindelin, J., Arganda-Carreras, I., Preibisch, S., Longair, M., Tomancak, P., Hartenstein, V., and Douglas, R.J. (2012). TrakEM2 software for neural circuit reconstruction. PLoS One *7*, e38011.

Carlier, M.F., Laurent, V., Santolini, J., Melki, R., Didry, D., Xia, G.X., Hong, Y., Chua, N.H., and Pantaloni, D. (1997). Actin depolymerizing factor (ADF/cofilin) enhances the rate of filament turnover: implication in actin-based motility. J. Cell Biol. *136*, 1307–1322.

(C) Endogenous cavin-1 and F-actin in control or ROCK1 knockdown cells in CDM, MIPs shown.
(D) Line profiles of cavin-1 intensity across the rear portion of cells as in (C) (N>20 cells/condition, 3 repeats, bars = SEM).
(E) Distance of peak cavin-1 intensity from the rear of cells as in (C) (N>20 cells/condition, 3 repeats).
(F) Endogenous cavin-1 and F-actin in control or Ect2 knockdown cells in CDM, MIPs shown.
(G) A2780 cell in CDM stained with Flipper-TR as in Figure 1F, pre- and 30 min post-Y27632 treatment. Top shows photon counts per pixel, bottom shows lifetime per pixel; right: unpaired (top) and pairwise (bottom) average rear—front Flipper-TR lifetime difference pre- and post-Y27632 treatment (N>9 cells/condition, 3 repeats).
(H) A2780 cells in CDM imaged in the presence of caged-CytoD before and after uncaging of a specific region behind the cell rear (indicated by yellow box). Rear caveolin-1 intensity in cells before and 10 min after uncaging (N>30 cells/condition, 3 repeats).
$^{**}p < 0.01$; $^{***}p < 0.001$; $^{****}p < 0.0001$. See also Figure S7 and Videos S6 and S7.

Casella, J.F., Flanagan, M.D., and Lin, S. (1981). Cytochalasin D inhibits actin polymerization and induces depolymerization of actin filaments formed during platelet shape change. Nature *293*, 302–305.

Caswell, P.T., Spence, H.J., Parsons, M., White, D.P., Clark, K., Cheng, K.W., Mills, G.B., Humphries, M.J., Messent, A.J., Anderson, K.I., et al. (2007). Rab25 associates with alpha5beta1 integrin to promote invasive migration in 3D microenvironments. Dev. Cell *13*, 496–510.

Caswell, P.T., and Zech, T. (2018). Actin-based cell protrusion in a 3D matrix. Trends Cell Biol. *28*, 823–834.

Cheng, J.P.X., Mendoza-Topaz, C., Howard, G., Chadwick, J., Shvets, E., Cowburn, A.S., Dunmore, B.J., Crosby, A., Morrell, N.W., and Nichols, B.J. (2015). Caveolae protect endothelial cells from membrane rupture during increased cardiac output. J. Cell Biol. *211*, 53–61.

Colom, A., Derivery, E., Soleimanpour, S., Tomba, C., Molin, M.D., Sakai, N., González-Gaitán, M., Matile, S., and Roux, A. (2018). A fluorescent membrane tension probe. Nat. Chem. *10*, 1118–1125.

Cramer, L.P. (2013). Mechanism of cell rear retraction in migrating cells. Curr. Opin. Cell Biol. *25*, 591–599.

Cukierman, E., Pankov, R., Stevens, D.R., and Yamada, K.M. (2001). Taking cell-matrix adhesions to the third dimension. Science *294*, 1708–1712.

del Pozo, M.A., Balasubramanian, N., Alderson, N.B., Kiosses, W.B., Grande-García, A., Anderson, R.G.W., and Schwartz, M.A. (2005). Phospho-caveolin-1 mediates integrin-regulated membrane domain internalization. Nat. Cell Biol. *7*, 901–908.

Denais, C.M., Gilbert, R.M., Isermann, P., Mcgregor, A.L., Lindert, M., Weigelin, B., Davidson, P.M., Friedl, P., and Lammerding, J. (2016). Nuclear envelope rupture and repair during cancer cell migration. Science *352*, 1–41.

Diz-Muñoz, A., Fletcher, D.A., and Weiner, O.D. (2013). Use the force: membrane tension as an organizer of cell shape and motility. Trends Cell Biol. *23*, 47–53.

Echarri, A., and Del Pozo, M.A. (2015). Caveolae - mechanosensitive membrane invaginations linked to actin filaments. J. Cell Sci. *128*, 2747–2758.

Friedl, P., and Alexander, S. (2011). Cancer invasion and the microenvironment: plasticity and reciprocity. Cell *147*, 992–1009.

Garcia, J., Bagwell, J., Njaine, B., Norman, J., Levic, D.S., Wopat, S., Miller, S.E., Liu, X., Locasale, J.W., Stainier, D.Y.R., et al. (2017). Sheath cell invasion and trans-differentiation repair mechanical damage caused by loss of caveolae in the zebrafish notochord. Curr. Biol. *27*, 1982–1989.e3.

Gauthier, N.C., Masters, T.A., and Sheetz, M.P. (2012). Mechanical feedback between membrane tension and dynamics. Trends Cell Biol. *22*, 527–535.

Gautreau, A., Louvard, D., and Arpin, M. (2000). Morphogenic effects of ezrin require a phosphorylation-induced transition from oligomers to monomers at the plasma membrane. J. Cell Biol. *150*, 193–203.

Gingras, D., Gauthier, F., Lamy, S., Desrosiers, R.R., and Béliveau, R. (1998). Localization of RhoA GTPase to endothelial caveolae-enriched membrane domains. Biochem. Biophys. Res. Commun *247*, 888–893.

Goetz, J.G., Minguet, S., Navarro-Lérida, I., Lazcano, J.J., Samaniego, R., Calvo, E., Tello, M., Osteso-Ibáñez, T., Pellinen, T., Echarri, A., et al. (2011). Biomechanical remodeling of the microenvironment by stromal caveolin-1 favors tumor invasion and metastasis. Cell *146*, 148–163.

Grande-García, A., and del Pozo, M.A. (2008). Caveolin-1 in cell polarization and directional migration. Eur. J. Cell Biol. *87*, 641–647.

Grande-García, A., Echarri, A., de Rooij, J., Alderson, N.B., Waterman-Storer, C.M., Valdivielso, J.M., and del Pozo, M.A. (2007). Caveolin-1 regulates cell polarization and directional migration through Src kinase and Rho GTPases. J. Cell Biol. *177*, 683–694.

Hamaguchi, T., Ito, M., Feng, J., Seko, T., Koyama, M., Machida, H., Takase, K., Amano, M., Kaibuchi, K., Hartshorne, D.J., et al. (2000). Phosphorylation of CPI-17, an inhibitor of myosin phosphatase, by protein kinase N. Biochem. Biophys. Res. Commun. *274*, 825–830.

Harper, K.L., Sosa, M.S., Entenberg, D., Hosseini, H., Cheung, J.F., Nobre, R., Avivar-Valderas, A., Nagi, C., Girnius, N., Davis, R.J., et al. (2016). Mechanism of early dissemination and metastasis in Her2+mammary cancer. Nature *540*, 588–592.

Hayer, A., Stoeber, M., Bissig, C., and Helenius, A. (2010). Biogenesis of caveolae: stepwise assembly of large caveolin and cavin complexes. Traffic *11*, 361–382.

Hermanowicz, P., Sarna, M., Burda, K., and Gabryś, H. (2014). AtomicJ: an open source software for analysis of force curves. Rev. Sci. Instrum *85*, 063703.

Hetmanski, J.H.R., Zindy, E., Schwartz, J.M., and Caswell, P.T. (2016). A MAPK-driven feedback loop suppresses Rac activity to promote RhoA-driven cancer cell invasion. PLoS Comput. Biol. *12*, e1004909.

Hoernke, M., Mohan, J., Larsson, E., Blomberg, J., Kahra, D., Westenhoff, S., Schwieger, C., and Lundmark, R. (2017). EHD2 restrains dynamics of caveolae by an ATP-dependent, membrane-bound, open conformation. Proc. Natl. Acad. Sci. USA *114*, E4360–E4369.

Hoops, S., Sahle, S., Gauges, R., Lee, C., Pahle, J., Simus, N., Singhal, M., Xu, L., Mendes, P., and Kummer, U. (2006). COPASI - a COmplex PAthway SImulator. Bioinformatics *22*, 3067–3074.

Irianto, J., Xia, Y., Pfeifer, C.R., Athirasala, A., Ji, J., Alvey, C., Tewari, M., Bennett, R.R., Harding, S.M., Liu, A.J., et al. (2017). DNA damage follows repair factor depletion and portends genome variation in cancer cells after pore migration. Curr. Biol. *27*, 210–223.

Ishizaki, T., Uehata, M., Tamechika, I., Keel, J., Nonomura, K., Maekawa, M., and Narumiya, S. (2000). Pharmacological properties of Y-27632, a specific inhibitor of rho-associated kinases. Mol. Pharmacol. *57*, 976–983.

Jacquemet, G., Green, D.M., Bridgewater, R.E., von Kriegsheim, A., Humphries, M.J., Norman, J.C., and Caswell, P.T. (2013). RCP-driven $\alpha5\beta1$ recycling suppresses Rac and promotes RhoA activity via the RacGAP1-IQGAP1 complex. J. Cell Biol. *202*, 917–935.

Keren, K. (2011). Cell motility: the integrating role of the plasma membrane. Eur. Biophys. J *40*, 1013–1027.

Keren, K., Pincus, Z., Allen, G.M., Barnhart, E.L., Marriott, G., Mogilner, A., and Theriot, J.A. (2008). Mechanism of shape determination in motile cells. Nature *453*, 475–480.

Kotýnková, K., Su, K.C., West, S.C., and Petronczki, M. (2016). Plasma membrane association but not midzone recruitment of RhoGEF ECT2 is essential for cytokinesis. Cell Rep *17*, 2672–2686.

Lachmann, S., Jevons, A., de Rycker, M., Casamassima, A., Radtke, S., Collazos, A., and Parker, P.J. (2011). Regulatory domain selectivity in the cell-type specific PKN-dependence of cell migration. PLoS One *6*, e21732.

Lamaze, C., and Torrino, S. (2015). Caveolae and cancer: a new mechanical perspective. Biomed. J. *38*, 367–379.

Latorre, E., Kale, S., Casares, L., Gómez-González, M., Uroz, M., Valon, L., Nair, R.V., Garreta, E., Montserrat, N., del Campo, A., et al. (2018). Active superelasticity in three-dimensional epithelia of controlled shape. Nature *563*, 203–208.

Le Novère, N., Bornstein, B., Broicher, A., Courtot, M., Donizelli, M., Dharuri, H., Li, L., Sauro, H., Schilstra, M., Shapiro, B., et al. (2006). BioModels Database: a free, centralized database of curated, published, quantitative kinetic models of biochemical and cellular systems. Nucleic Acids Res. *34*, D689–D691.

Lentini, D., Guzzi, F., Pimpinelli, F., Zaninetti, R., Cassetti, A., Coco, S., Maggi, R., and Parenti, M. (2008). Polarization of caveolins and caveolae during migration of immortalized neurons. J. Neurochem. *104*, 514–523.

Lieber, A.D., Schweitzer, Y., Kozlov, M.M., and Keren, K. (2015). Front-to-rear membrane tension gradient in rapidly moving cells. Biophys. J. *108*, 1599–1603.

Lim, Y.W., Lo, H.P., Ferguson, C., Martel, N., Giacomotto, J., Gomez, G.A., Yap, A.S., Hall, T.E., and Parton, R.G. (2017). Caveolae protect notochord cells against catastrophic mechanical failure during development. Curr. Biol. *27*, 1968–1981.e7.

Ludwig, A., Howard, G., Mendoza-Topaz, C., Deerinck, T., Mackey, M., Sandin, S., Ellisman, M.H., and Nichols, B.J. (2013). Molecular composition and ultrastructure of the caveolar coat complex. PLoS Biol. *11*, e1001640.

Matthews, H.K., Delabre, U., Rohn, J.L., Guck, J., Kunda, P., and Baum, B. (2012). Changes in Ect2 localization couple actomyosin-dependent cell shape changes to mitotic progression. Dev. Cell *23*, 371–383.

Morgan, M., Perry, S.V., and Ottaway, J. (1976). Myosin light-chain phosphatase. Biochem. J. *157*, 687–697.

Mueller, J., Szep, G., Nemethova, M., de Vries, I., Lieber, A.D., Winkler, C., Kruse, K., Small, J.V., Schmeiser, C., Keren, K., et al. (2017). Load adaptation of lamellipodial actin networks. Cell *171*, 188–200.e16.

Neill, P.R.O., Castillo-Badillo, J.A., Meshik, X., Kalyanaraman, V., Melgarejo, K., Gautam, N., Neill, P.R.O., Castillo-Badillo, J.A., Meshik, X., Kalyanaraman, V., et al. (2018). Membrane flow drives an adhesion-independent amoeboid cell migration mode article membrane flow drives an adhesion-independent amoeboid cell migration mode. Dev. Cell *46*, 1–3.

Ohashi, K., Nagata, K., Maekawa, M., Ishizaki, T., Narumiya, S., and Mizuno, K. (2000). Rho-associated kinase ROCK activates LIM-kinase 1 by phosphorylation at threonine 508 within the activation loop. J. Biol. Chem. *275*, 3577–3582.

Paluch, E.K., Aspalter, I.M., and Sixt, M. (2016). Focal adhesion – independent cell migration. Annu. Rev. Cell Dev. Biol. *32*, 469–490.

Parat, M.O., Anand-Apte, B., and Fox, P.L. (2003). Differential Caveolin-1 polarization in endothelial cells during migration in two and three dimensions. Mol. Biol. Cell *14*, 3156–3168.

Parton, R.G., and del Pozo, M.A. (2013). Caveolae as plasma membrane sensors, protectors and organizers. Nat. Rev. Mol. Cell Biol. *14*, 98–112.

Paul, N.R., Allen, J.L., Chapman, A., Morlan-Mairal, M., Zindy, E., Jacquemet, G., Fernandez del Ama, L., Ferizovic, N., Green, D.M., Howe, J.D., et al. (2015). alpha-5 beta-1 integrin recycling promotes Arp2/3-independent cancer cell invasion via the formin FHOD3. J. Cell Biol. *210*, 1013–1031.

Petrie, R.J., Gavara, N., Chadwick, R.S., and Yamada, K.M. (2012). Nonpolarized signaling reveals two distinct modes of 3D cell migration. J. Cell Biol. *197*, 439–455.

Piekny, A.J., and Glotzer, M. (2008). Anillin is a scaffold protein that links RhoA, actin, and myosin during cytokinesis. Curr. Biol. *18*, 30–36.

Pontes, B., Monzo, P., Gole, L., Le Roux, A.L., Kosmalska, A.J., Tam, Z.Y., Luo, W., Kan, S., Viasnoff, V., Roca-Cusachs, P., et al. (2017). Membrane tension controls adhesion positioning at the leading edge of cells. J. Cell Biol. *216*, 2959–2977.

Priya, R., Gomez, G.A., Budnar, S., Verma, S., Cox, H.L., Hamilton, N.A., and Yap, A.S. (2015). Feedback regulation through myosin II confers robustness on RhoA signaling at E-cadherin junctions. Nat. Cell Biol. *17*, 1282–1293.

Quétier, I., Marshall, J.J.T., Spencer-Dene, B., Lachmann, S., Casamassima, A., Franco, C., Escuin, S., Worrall, J.T., Baskaran, P., Rajeeve, V., et al. (2016). Knockout of the PKN family of rho effector kinases reveals a non-redundant role for PKN2 in developmental mesoderm expansion. Cell Rep *14*, 440–448.

Raab, M., Gentili, M., De Belly, H., Thiam, H.R., Vargas, P., Jimenez, A.J., Lautenschlaeger, F., Voituriez, R., Lennon-Duménil, A.M., Manel, N., et al. (2016). ESCRT III repairs nuclear envelope ruptures during cell migration to limit DNA damage and cell death. Science *352*, 359–362.

Raab, M., Swift, J., Dingal, P.C.D.P., Shah, P., Shin, J.W., and Discher, D.E. (2012). Crawling from soft to stiff matrix polarizes the cytoskeleton and phosphoregulates myosin-II heavy chain. J. Cell Biol. *199*, 669–683.

Ratheesh, A., Gomez, G.A., Priya, R., Verma, S., Kovacs, E.M., Jiang, K., Brown, N.H., Akhmanova, A., Stehbens, S.J., and Yap, A.S. (2012). Centralspindlin and α-catenin regulate Rho signaling at the epithelial zonula adherens. Nat. Cell Biol. *14*, 818–828.

Ridley, A.J. (2011). Life at the leading edge. Cell *145*, 1012–1022.

Ridley, A.J., Schwartz, M.A., Burridge, K., Firtel, R.A., Ginsberg, M.H., Borisy, G., Parsons, J.T., and Horwitz, A.R. (2003). Cell migration: integrating signals from front to back. Science *302*, 1704–1709.

Roberts, D.L., O'Dwyer, S.T., Stern, P.L., and Renehan, A.G. (2015). Global gene expression in pseudomyxoma peritonei, with parallel development of two immortalized cell lines. Oncotarget *6*, 10786–10800.

Rouven Brückner, B., Pietuch, A., Nehls, S., Rother, J., and Janshoff, A. (2015). Ezrin is a major regulator of membrane tension in epithelial cells. Sci. Rep *5*, 14700.

Sastry, S.K., Rajfur, Z., Liu, B.P., Cote, J.F., Tremblay, M.L., and Burridge, K. (2006). PTP-PEST couples membrane protrusion and tail retraction via VAV2 and p190RhoGAP. J. Biol. Chem. *281*, 11627–11636.

Shi, Z., Graber, Z.T., Baumgart, T., Stone, H.A., and Cohen, A.E. (2018). Cell membranes resist flow. Cell *175*, 1769–1779.e13.

Sinha, B., Köster, D., Ruez, R., Gonnord, P., Bastiani, M., Abankwa, D., Stan, R.V., Butler-Browne, G., Vedie, B., Johannes, L., et al. (2011). Cells respond to mechanical stress by rapid disassembly of caveolae. Cell *144*, 402–413.

Starborg, T., Kalson, N.S., Lu, Y., Mironov, A., Cootes, T.F., Holmes, D.F., and Kadler, K.E. (2013). Using transmission electron microscopy and 3View to determine collagen fibril size and three-dimensional organization. Nat. Protoc. *8*, 1433–1448.

Su, K.-C.C., Takaki, T., and Petronczki, M. (2011). Targeting of the RhoGEF Ect2 to the equatorial membrane controls cleavage furrow formation during cytokinesis. Dev. Cell *21*, 1104–1115.

Tse, J.R., and Engler, A.J. (2010). Preparation of hydrogel substrates with tunable mechanical properties. Curr. Protoc. Cell Biol., Chapter 10, Unit 10.16.

Urra, H., Torres, V.A., Ortiz, R.J., Lobos, L., Díaz, M.I., Díaz, N., Härtel, S., Leyton, L., and Quest, A.F.G. (2012). Caveolin-1-enhanced motility and focal adhesion turnover require tyrosine-14 but not accumulation to the rear in metastatic cancer cells. PLoS One *7*.

Van Helvert, S., and Friedl, P. (2016). Strain stiffening of fibrillar collagen during individual and collective cell migration identified by AFM nanoindentation. ACS Appl. Mater. Interfaces *8*, 21946–21955.

Vicente-Manzanares, M., Ma, X., Adelstein, R.S., and Horwitz, A.R. (2009). Non-muscle myosin II takes center stage in cell adhesion and migration. Nat. Rev. Mol. Cell Biol. *10*, 778–790.

Williams, M.E., Wilke, S.A., Daggett, A., Davis, E., Otto, S., Ravi, D., Ripley, B., Bushong, E.A., Ellisman, M.H., Klein, G., et al. (2011). Cadherin-9 regulates synapse-specific differentiation in the developing hippocampus. Neuron *71*, 640–655.

Wolf, K., te Lindert, M., Krause, M., Alexander, S., te Riet, J., Willis, A.L., Hoffman, R.M., Figdor, C.G., Weiss, S.J., and Friedl, P. (2013). Physical limits of cell migration: control by ECM space and nuclear deformation and tuning by proteolysis and traction force. J. Cell Biol. *201*, 1069–1084.

Yang, N., Higuchi, O., Ohashi, K., Nagata, K., Wada, A., Kangawa, K., Nishida, E., and Mizuno, K. (1998). Cofilin phosphorylation by LIM-kinase 1 and its role in Rac-mediated actin reorganization. Nature *393*, 809–812.

Yoshizaki, H., Ohba, Y., Kurokawa, K., Itoh, R.E., Nakamura, T., Mochizuki, N., Nagashima, K., and Matsuda, M. (2003). Activity of Rho-family GTPases during cell division as visualized with FRET-based probes. J. Cell Biol. *162*, 223–232.

Zimnicka, A.M., Husain, Y.S., Shajahan, A.N., Sverdlov, M., Chaga, O., Chen, Z., Toth, P.T., Klomp, J., Karginov, A.V., Tiruppathi, C., et al. (2016). Src-dependent phosphorylation of caveolin-1 Tyr-14 promotes swelling and release of caveolae. Mol. Biol. Cell *27*, 2090–2106.

STAR★METHODS

KEY RESOURCES TABLE

REAGENT or RESOURCE	SOURCE	IDENTIFIER
Antibodies		
Mouse anti-Fibronectin	Abcam	Cat#ab6328; RRID: AB_305428
Rabbit anti-Caveolin-1	BD Biosciences	Cat# 610060
Mouse anti-EHD2	Santa Cruz Biotechnology	Cat# sc-100724; RRID:AB_2246399
Goat anti-EHD2	Abcam	Cat# ab23935; RRID:AB_2097328
Rabbit anti-PTRF	Abcam	Cat# ab48824; RRID:AB_882224
Rabbit anti-RhoA	Santa Cruz Biotechnology	Cat# sc-179, RRID:AB_632346
Rabbit anti-ROCK1	Cell Signalling	Cat# 4035; RRID:AB_2238679
Rabbit anti-ROCK2	Cell Signalling	Cat# 9029; RRID:AB_11127802
Mouse anti-Ect2	Santa Cruz Biotechnology (for IF)	Cat# sc-514750
Rabbit anti-Ect2	Gift from Dr M. Petronczki (for WB)	Su et al., 2011
Rabbit anti-Vav2	Abcam	Cat# ab52640; RRID:AB_2241456
Rabbit anti-ARHGEF2	GeneTex	Cat# GTX125893; RRID:AB_11177391
Mouse anti-PLEKHG5	Novus Biologicals	Cat# H00057449-M01; RRID:AB_2166792
Rabbit anti-DOCK1	ProteinTech	Cat# 20244-1-AP; RRID:AB_10665358
Goat anti-ELMO2	Abcam	Cat# ab2240; RRID:AB_2099432
Mouse anti-Tubulin	Abcam	Cat# ab7291; RRID:AB_2241126
Mouse anti-Akt-2	Santa Cruz Biotechnology	Cat# sc-5270; RRID:AB_626659
Rabbit anti-Erk	Santa Cruz Biotechnology	Cat# sc-154; RRID: AB_2141292
Chemicals, Peptides, and Recombinant Proteins		
Y-27632 2HCl	AdooQ Bioscience	Cat #: A11001
H-1152	Tocris	Cat #: 2412
Cytochalasin-D	Sigma Aldrich	Cat#: C8273
Caged Cytochalasin-D	Latorre et al., 2018	N/A
Deposited Data		
ODE model ‘Hetmanski 2019 cell rear’	BioModels (La Novere et al., 2006)	ebi.ac.uk/biomodels/MODEL1908290001
Experimental Models: Cell Lines		
Human: A2780 ovarian cancer cell line	Gift from Prof. Gordon Mills	Caswell et al., 2007
Human: H1299 lung cancer cell line	ATCC	CRL-5803
Human: N15A Pseudomyxoma peritonei (PMP)	Gift from Prof. A Renehan	Roberts et al., 2015
Mouse: Embryonic fibroblasts (MEFs)	Gift from Dr M. Bass	N/A
Rat: Telomerase immortalized fibroblasts (TIFs)	Gift from Prof. Tom Curran	Caswell et al., 2007
Oligonucleotides		
Cav-1 - individual Hs_CAV1_9 FlexiTube 5′ AAGCATCAACTTGCAGAAAGA	Qiagen	Cat# SI00299635
Cav-1 - individual Hs_CAV1_10 FlexiTube 5′ AAGCAAGTGTACGACGCGCAC	Qiagen	Cat# SI00299642
EHD2 - pooled FlexiTube GeneSolution	Qiagen	GS30846
RhoA - 5′ ATGGAAAGCAGGTAGAGTT	Eurofins	Custom (Jacquemet et al., 2013)
ROCK1 - Silencer Select Pre-Designed 5′ -GCTTGTAGGTGATACACCTTT	Life Technologies	ID# s12099
ROCK2 - Silencer Select Pre-Designed 5′-GAGATTACCTTACGGAAAATT	Life Technologies	ID# s18162

(Continued on next page)

Continued		
REAGENT or RESOURCE	SOURCE	IDENTIFIER
PKN1 - SMARTpool siRNA	Dharmacon	Cat# L-004175-00-0005
PKN2- SMARTpool siRNA	Dharmacon	Cat# L-004612-00-0005
PKN3 - SMARTpool siRNA	Dharmacon	Cat# L-004647-00-0005
PKN-2 - individual siRNA 5′ GGAGC GCTCTGATGGACA	Eurofins	Custom
Ect2 - individual Hs_Ect2_5 FlexiTube 5′ TTGCCTAGAGATAGCAAGAAA	Qiagen	Cat# SI02643067
Ect2 - individual Hs_Ect2_7 FlexiTube 5′ GTCGCCCGTTGTATTGTACAA	Qiagen	Cat# SI03106390
Ect2 - pooled FlexiTube GeneSolution	Qiagen	GS1894
Vav2 - pooled FlexiTube GeneSolution	Qiagen	GS7410
PLEKHG5 - pooled FlexiTube GeneSolution	Qiagen	GS57449
DOCK1 - pooled FlexiTube GeneSolution	Qiagen	GS1793
ELMO2 - pooled FlexiTube GeneSolution	Qiagen	GS63916
ARHGEF2 - SMARTpool: ON-TARGET plus ARHGEF2 siRNA	Dharmacon	Cat# J-00983-07-0005
Recombinant DNA		
Plasmid: FRET biosensor Raichu-1237X RhoA	Gift from Prof M. Matsuda; Yoshizaki et al., 2003	N/A
Plasmid: mCherry-Caveolin-1	Gift from Dr M. Bass	N/A
Plasmid: GFP-AHPH Anillin	Gift from Prof A. Yap; Priya et al., 2015	N/A
Plasmid: Emerald-Lifeact	Gift from Dr C. Ballestrem	N/A
Plasmid: farnesylGFP (GFP-membrane)	Gift from Prof J. Norman	N/A
Plasmid: GFP-Paxillin	Gift from Prof M. Humphries	N/A
Plasmid: GFP-Ezrin wild type (WT)	Gautreau et al., 2000	N/A
Plasmid: GFP-Ezrin constitutively active (CA)	Gautreau et al., 2000	N/A
Software and Algorithms		
FIJI v1.51	ImageJ	fiji.sc/
Copasi 4.15	Hoops et al., 2006	copasi.org/
Matlab R2017a	Mathworks	mathworks.com/products/matlab
AtomicJ	Hermanowicz et al., 2014	sourceforge.net/projects/jrobust/
Prism 7.03	GraphPad	graphpad.com/scientific-software/prism/
Other		
Mag-Trypsin beads	TaKaRa	Cat# 635646
Flipper-TR	Spirochrome	Cat# SC020; Colom et al., 2016
SIR-Actin	Spirochrome	Cat# SC001
Alexa Fluor 488 Phalloidin	Invitrogen	Cat# A12379

LEAD CONTACT AND MATERIALS AVAILABILITY

Further information and requests for resources and reagents should be directed to and will be fulfilled by the Lead Contact, Patrick Caswell (patrick.caswell@manchester.ac.uk).

EXPERIMENTAL MODEL AND SUBJECT DETAILS

Cell Culture and Transient Transfection

A2780 human ovarian cancer cells (female) were maintained in RPMI-1640 medium (Sigma-Aldrich) supplemented with 10% (v/v) fetal calf serum, 1% (v/v) L-Glutamine and (v/v) 1% Antibiotic-antimycotic (both Sigma-Aldrich); telomerase-immortalized fibroblasts (TIF) cells, mouse-embryonic fibroblasts (MEF) cells and H1299 human lung cancer cells(male) were maintained in Dulbecco's modified Eagle's medium (DMEM, Sigma-Aldrich) containing L-Glutamine and supplemented with 10% (v/v) fetal calf serum, and (v/v) 1%

Antibiotic-antimycotic (Sigma Aldrich); and N15A(Roberts et al., 2015) Pseudomyxoma peritonei (PMP) cells were maintained in Dulbecco's modified Eagle's medium (DMEM) (Sigma Aldrich) supplemented with 10% FCS, 25 mM HEPES, 5 μg/ml Insulin, 10 mM L-Glutamine and (v/v) 1% Antibiotic-antimycotic (Sigma Aldrich). All cell lines were incubated at 37 °C in a humidified 5% (v/v) CO2 atmosphere. All siRNAs and fluorescent constructs were transiently transfected by electroporation using a nucleofector (Amaxa, Lonza) using solution T, program A-23, 3ug DNA / 5μl 20 mM siRNA as per the manufacturer's instructions. Experiments were performed ~24 h after nucleofection unless otherwise stated.

METHODS DETAILS

Reagents Usage Conditions

ROCK/PKN inhibitor Y-27632 (Ishizaki et al., 2000) was used at 10 μM; ROCK inhibitor H-1152 was used at 2 μM; actin polymerization inhibitor Cytochalasin-D (Sigma) was used at 2 μM; caged Cytochalasin-D (Latorre et al., 2018) was used at 50 μM as outlined below. siRNAs were as follows: Cav-1 – individual Hs_CAV1_9 FlexiTube 5′ AAGCATCAACTTGCAGAAAGA (Qiagen) and Hs_CAV1_10 FlexiTube 5′ AAGCAAGTGTACGACGCGCAC (Qiagen) (referred to as Cav-1 siRNA-A and Cav-1 siRNA-B respectively here, all Cav-1 knockdown experiments were performed with each individual siRNA at separate times in comparison with separate control siRNAs); EHD2 – pooled FlexiTube GeneSolution (Qiagen); RhoA – 5′ ATGGAAAGCAGGTAGAGTT (Eurofins); ROCK1 – Silencer Select Pre-Designed (Life Technologies, 5′ -GCTTGTAGGTGATACACCTTT); ROCK2 – Silencer Select Pre-Designed (Life Technologies, 5′-GAGATTACCTTACGGAAAATT); PKN1-3- SMARTpool siRNA (Dharmacon); PKN-2 – individual 5′ GGAGCGCTCTGAT GGACA (Eurofins); Ect2 – individual Hs_Ect2_5 FlexiTube 5′ TTGCCTAGAGATAGCAAGAAA and Hs_Ect2_7 FlexiTube 5′ GTCGCCCGTTGTATTGTACAA; Ect2, Vav2, PLEKHG5, DOCK1, ELMO2 – all pooled Flexitube GeneSolution (Qiagen); ARHGEF2 - SMARTpool: ON-TARGET plus ARHGEF2 siRNA. Sufficient knockdown was achieved with a single transfection after 24h for Cav-1-A; after 72h for ROCK1 and ROCK2; and double transfection after 2x24h for Cav-1-B, RhoA, EHD2, Ect2-A, Ect2-B, Ect2 (pool), Vav2, PLEKHG5, DOCK1, ELMO2 and ARHGEF2. FRET biosensor Raichu-1237X (Yoshizaki et al., 2003) RhoA was kindly provided by Prof. M. Matsuda; mCherry-Caveolin-1 construct (Hayer et al., 2010) was kindly provided by Dr M. Bass; the GFP-AHPH Anillin construct (Priya et al., 2015) kindly provided by Prof. A. Yap; Emerald-Lifeact construct was kindly provided by Dr C. Ballestrem; farnesylGFP (GFP-membrane) construct was kindly provided by Prof. J Norman; GFP-Paxillin construct was kindly provided by Prof. M. Humphries; GFP-Ezrin wild type (WT) and GFP-Ezrin constitutively active (CA) were from (Gautreau et al., 2000). The following antibodies were used: Mouse anti-Fibronectin (Sigma-Aldrich); Rabbit anti-Caveolin-1 (BD Biosciences); Mouse anti-EHD2 (Santa Cruz Biotechnology, for western blot); Goat anti-EHD2 (Abcam, for IF); Rabbit anti-PTRF (Abcam); Rabbit anti-RhoA (Santa Cruz Biotechnology); Rabbit anti-ROCK1 and Rabbit anti-ROCK2 (both Cell Signalling); Mouse anti-Ect2 (Santa Cruz Biotechnology, for IF); Rabbit anti-Ect2 (kind gift from Dr M. Petronczki, for western blot); Rabbit anti-Vav2 (Abcam); Rabbit anti-ARHGEF2 (GeneTex); Mouse anti-PLEKHG5 (Novus Biologicals); Rabbit anti-DOCK1 (ProteinTech); Goat anti-ELMO2 (Abcam); Mouse anti-Tubulin (DM1A), Mouse anti Akt-2 and Rabbit anti-Erk (Santa Cruz Biotechnology).

SDS-PAGE and Quantitative Western Blotting

Cells were lysed in non-denaturing lysis buffer (200 mM NaCl, 75 mM Tris-HCl, pH 7.4, 15 mM NaF, 1.5 mM Na3VO4, 7.5 mM EDTA, 7.5 mM EGTA, 1.5% (v/v) Triton X-100, 0.75% (v/v) NP-40, 50 μg/ml leupeptin, 50 μg/ml aprotinin, and 1 mM 4-(2-aminoethyl)-benzenesulfonyl fluoride). Lysates were clarified by centrifugation at 10,000 g for 10 min at 4°C. Cell lysates were resolved under denaturing conditions by SDS-PAGE (4–12% Bis-Tris gels; Invitrogen) and transferred to nitrocellulose membrane. Membranes were blocked with 1x Blocking Buffer (Sigma) and incubated overnight at 4°C with the appropriate primary antibody in 5% BSA and then at room temperature for 1 h with the appropriate fluorophore-conjugated secondary antibody in 1x Blocking Buffer. Membranes were scanned using an infrared imaging system (Odyssey; LI-COR Biosciences). siRNA efficiency was quantified in ImageJ following appropriate normalization to a relevant loading control.

CDM Production

Cell derived matrices were generated according to the method developed by the Yamada lab (Caswell et al., 2007; Cukierman et al., 2001). Briefly, 6/12-well plastic (Corning) or 35 mm glass bottom (Mattek) plates were coated with 0.2% gelatin (v/v, Sigma Aldrich), crosslinked with 1% glutaraldehyde (v/v, Sigma Aldrich) and quenched with 1M glycine (Thermo Fisher) before TIFs were confluently seeded. DMEM medium supplemented with 0.25% ascorbic acid (v/v, Sigma Aldrich) was changed every 48h for 8 days. Cells were denuded with extraction buffer (20 mM ammonium hydroxide (NH4OH); 0.5% (v/v) Triton X-100) to leave only matrix; finally DNAse was used for cleavage of phosphodiester linkages in the DNA backbone.

Gradient CDMs with a stiffness differential were created by softening a region of the CDM using trypsin-coated magnetic beads, as trypsin has been shown to alter the stiffness of CDM (Petrie et al., 2012). Specifically, 10 μl magnetic trypsin beads (TakaRa) were pipetted onto a CDM and localized with a magnet, such that a small, crescent shaped region of the CDM grown on the circular glass bottom area of the dish (Mattek) was subjected to a high concentration of trypsin beads. CDMs were incubated with magnetic trypsin beads for 10 min at 37 °C in a humidified 5% (v/v) CO2 atmosphere, before being washed 3x with PBS(+). Cells were plated onto non-uniform CDMs and imaged ~24 h after magnetic trypsin treatment, where the region of the interface of the softened, trypsin treated CDM (i.e. the area directly above the magnet during treatment) and the untreated CDM was carefully, manually identified and cells within this region subsequently analyzed.

Generation of Polyacrylamide Stiffness Gradients

Polyacrylamide gels were made according to the Tse and Engler (Tse and Engler, 2010) protocol with modification for gradient generation. Briefly, large area glass bottom plates (Nunc/Ibidi) or glass microscope slides (DeltaLab) were functionalized by coating with 0.1M NaOH then with 3-Aminopropyltriethoxysilane (APES, Sigma Aldrich), before being crosslinked with 0.5% glutaraldehyde (Sigma Aldrich) and dried; meanwhile manually cut 18 x 6 mm coverslips were rendered hydrophobic by coating with Dichlorodimethylsilane (DCDMS) for 4 min before also being dried. Statically compliant hydrogels were prepared by mixing appropriate concentrations of 40% (w/v) acrylamide, 2% (w/v) bis-acrylamide stock solutions (both Sigma-Aldrich) and distilled water and rapidly polymerized by the enzymatic activity of 1/100 10% (w/v) ammonium persulfate (APS) and 1/1000 Tetramethylethylenediamine (TEMED, both Sigma Aldrich). Acrylamide/bis-acrylamide concentrations for our two gels were: soft – 7.5% acrylamide, 1.5% bis-acrylamide, 91% H_2O; and stiff - 25% acrylamide, 11.25% bis-acrylamide, 63.75% H_2O. Gradients were formed by pipetting 2.7 or 5.4 μl of two different stiffnesses of gel (depending on working distance requirements for subsequent microscopy) approx. 15 mm apart on the aforementioned functionalized glass before engaging the 6 x 18 mm hydrophobic coverslips on the top surface of both gels. Natural diffusion and fluid flow of the two different gels into each other formed a confined 1D gradient covering the 6 x 18 mm area at an approx. thickness of 50 or 100 μm. Hydrophobic coverslips were removed in distilled water. Gels were then uniformly covered with the heterobifunctional protein cross-linker 0.2mg/ml sufosuccinimidyl-6-(4-azido-2-nitrophenylamino)-hexanoate (sulfo-SANPAH; Thermo-fisher) which required activation by exposure with a 365-nm UV light source for 10 min. Finally, cells were coated with 10 μg/ml fibronectin in 50 nM Hepes buffer for 16 h before use.

Atomic Force Microscopy of 3D-CDM

For front/rear stiffness measurements, the cells were kept at 37°C within a PetriDishHeater™ (JPK Instruments AG) for the duration of the experiment. Cantilever calibration was conducted using the contact-free thermal noise method (at 37°C within medium) prior to each experiment. Measurements of matrix stiffness were conducted with a threshold of 0.5nN and approach speed of 3μm/s. To obtain front and rear measurements the cantilever was manually positioned close to the pseudopod or cell rear, as determined by observation of direction of cell migration, and five repeat force/distance curves were obtained. To obtain stiffness gradient measurements a series of positions were probed; with an initial measurement at the border of a trypsinized area, followed by additional positions at ~600μm steps away from the area. At each position, an 8x8 grid of force/distance curves was collected within a 100μm^2 area, with 25-100 readings per area.

All force/distance curves were analyzed using the AtomicJ software (Hermanowicz et al., 2014). Contact points were automatically calculated via the robust golden methodology, and curves fitted using the Sneddon model for spherical indenters assuming a Poisson ratio of 0.5. Mis-identified contact points were manually corrected if the contact point was visually obvious. Curves that could not be accurately fitted by the model were excluded from further analysis. For front/rear measurements, the Young's Modulus for each curve was calculated and then each set of 5 were averaged to obtain a mean stiffness value. For gradient measurements, each set of 64 readings were averaged to obtain a mean stiffness value for the area at each position.

Atomic Force Microscopy of 2D Gradients

Reduced Young's Moduli of entire long-axis profiles of polyacrylamide gels were calculated using atomic force microscopy on a Bruker Catalyst mounted on a Nikon Eclipse Ti inverted light microscope, with a Bruker cantilever (OTESPA; 4-10nm tip radius). While the absolute long-axis of the gel containing the stiffness gradient was ~18 mm (see above), the total measurable distance by AFM was the middle 12-13 mm of the gel. Therefore, the far left reachable point was taken as distance point 0, the far right point was taken as normalized distance 1 and other points (center, three quarters, etc.) were measured and sampled relative to these extrema for all gels. End and center points were first measured, and then further points were iteratively sampled based on neighbors to locate the position of the steep gradient in all gels. N = 25 to 100 readings were taken around a 2.5-5 μm sided square for each reading. Three gradients were analyzed in this manner and showed clear consistency in absolute stiffness values and gradient location. AFM was performed on 100 μm thick gels.

Membrane Tension Measurements by Optical Trap

Membrane tension measurements were attained using the optical trap method. Tether pulling experiments were realized using a custom made optical trap (4W 1064nm Laser Quantum Ventus) with a 100x oil immersion objective (CFI Plan Fluor DLL, Nikon) on an inverted microscope (Nikon Eclipse TE2000-U) equipped with a motorized stage (PRIOR Proscan). The optical trap calibration was performed as described (Lieber et al., 2015). Measurements were performed by attaching concanavalin-A coated (50 μg/ml) latex beads (1.9μm diameter) to cells. Bead position was recorded in bright field after tether formation. Trap force was then calculated using a custom made ImageJ script. Trap force was measured twice for each cell (front and rear). All polyacrylamide gradients used for membrane tension measurements were 50 μm thick gels to fit within the working distance of the 100x objective.

Live Fluorescence Imaging

All live fluorescent images were acquired using a CSU-X1 spinning disc confocal (Yokagowa) on a Zeiss Axio-Observer Z1 microscope with a 63x/1.40 Plan-Apochromat objective for CDMs or a 63x/1.15 LD C-Apochromat Korr water objective or 100x/1.30 Plan-Neofluar objective for polyacrylamide gradients (as stated) to allow for greater working distance. An Evolve EMCCD camera (Photometrics) and motorized XYZ stage (ASI) was used. The 445, 488, 515 and 561nm lasers were controlled using an AOTF through

the laserstack (Intelligent Imaging Innovations (3I)) allowing both rapid 'shuttering' of the laser and attenuation of the laser power. Images were captured using SlideBook 6.0 software (3i). Randomly chosen representative polarized Lifeact-EGFP, Lifeact-Emerald, GFP-AHPH, GFPmembrane, Lifeact-RFP, mCherry-Caveolin-1, GFP-Cavin-1, GFP-Ezrin-WT, GFP-Ezrin-CA and GFP-paxillin expressing cells were captured with the appropriate excitation/emission spectrum and exposure time following ~ 4h spreading time on CDM and ~ 20h spreading time on gradient gels in Ham F12 medium or 1x Opti-Klear medium (Marker Gene Technologies Inc) supplemented with 10% (v/v) FCS (488 nm, 50 ms exposure for GFP-Lifeact or Emerald Lifeact; 488 nm, 10 ms exposure for GFP-AHPH; 488 nm, 100 ms exposure for GFP membrane, GFP-Ezrin-WT and GFP-Ezrin-CA; 561 nm, 50 ms exposure for RFP-Lifeact; 561 nm, 20 ms exposure for mCherry-Caveolin-1) every 15 s–1 min for 5–10 min as indicated. Where appropriate, rear translocation was measured for all imaged cells in ImageJ across this 5 min period by comparing the position of a part of the rear of the cell at the start of the timelapse with the exact same part of the rear at the end of the timelapse. For Colocalisation analysis of Cav-1 and Cavin-1, the Coloc2 tool in ImageJ was used to generate Pearson's correlation coefficients in manually derived rear and perinuclear regions. Images are pseudocolored using the red hot LUT (ImageJ) where appropriate to reveal differences in intensity.

Forster Resonance Energy Transfer (FRET) imaging of RhoA activity was performed and quantified as described in detail previously (Hetmanski et al., 2016). Concisely, randomly chosen polarized Raichu-RhoA expressing cells were captured every 15s for 5 min with 3 different excitation/emission spectra: CFP (445 nm) donor – CFP acceptor at 200 ms exposure; YFP (515 nm) donor – YFP acceptor at 50 ms exposure; and CFP donor – YFP acceptor at 200 ms exposure. Ratiometric FRET activity was then calculated by dividing the CFP-YFP channel by the CFP-CFP channel following automated image alignment. The YFP-YFP channel was used to create a binary mask of the cell as it had the best signal to noise. Using this mask, a ring of the outermost 40 pixels of the cell was created and the front and rear quarters of the cell were isolated based on long-axis identification. The average of every ratiometric pixel value in a 40 pixel wide, rearmost 25% area of the cell was then obtained and averaged over the 5 min timelapse period to obtain the final single 'average rear RhoA FRET ratio' value per cell.

Membrane Tension Measurements by FLIM

For membrane tension measurements for cell in 3D CDM, the fluorescent probe Flipper-TR was used. Cells were spread on CDM for 4 h in normal growth medium which was replaced with imaging medium (1x OptiKlear + 10% FCS) containing 1μM Flipper TR, and incubated at 37̊C for 15 min to achieve appropriate labelling prior to imaging. Cells were imaged on a SP8 gSTED microscope using PicoQuant hardware/software for fluorescent lifetime imaging (FLIM) with 488 nm excitation and 575-625 emission and a pixel dwell time of > 19.58 μs. Following imaging, a double exponential was fit to ensure a close fit (chi squared < 1.5) and the counts per pixel and lifetime per pixel images exported as 256x256 pixel single time-point, single Z-plane images. For quantification, membrane regions at the cell rear and cell front were manually identified using the count per pixel images, and the mean average lifetimes measured by assigning these front/rear ROIs to the lifetime per pixel images. For the lifetime representative images, the lifetime per pixel image was smoothed by application of a Gaussian blur with 1.0 pixel radius, multiplied by a mask created using the counts per pixel image to remove background/matrix lipid staining, and recolored with a 16 colour LUT.

Long Term Cell Migration Assay

Cells were seeded at sparse (<25%, ~50,000 cells/well) confluency in typical growth medium on 6/12-well CDMs and allowed to spread for ~ 6h. Images were acquired on an Eclipse Ti inverted microscope (Nikon) using a 20x/ 0.45 SPlan Fluar objective, the Nikon filter sets for Brightfield and a pE-300 LED (CoolLED) fluorescent light source with imaging software NIS Elements AR.46.00.0. Point visiting was used to allow multiple positions to be imaged within the same time-course and cells were maintained at 37°C and 5% CO2. The images were collected using a Retiga R6 (Q-Imaging) camera. 6 randomly chosen positions per cell were captured every 10 min over 16 h. 5 randomly chosen cells per position (meaning 30 cells tracked per condition per experiment) were individually manually tracked using the ImageJ plugin MTrackJ every 3 frames (i.e. using 30 min timepoint intervals). The Chemotaxis and Migration Tool was used to calculate the average speed and representative images of individual cells are shown where appropriate.

Fixed Cell High Resolution Imaging

Cells were fixed in 4% paraformaldehyde (PFA) at room temperature following ~ 4h spreading on CDM or ~ 20h spreading on polyacrylamide gradients after non-confluent (~50,000 cells/plate) seeding. Membranes were permeabilized with 0.2% (v/v) Triton-X and blocked in 5% (w/v) heat-denatured bovine serum albumin (BSA) before being stained with appropriate antibodies as in reagents section. Cells were incubated with secondary antibodies Rabbit Alexa Fluor 647-conjugated, Rabbit Alexa Fluor 594-conjugated, Rabbit Alexa Fluor 405-conjugated, Mouse Alexa Fluor 594 conjugated, Goat Alexa Fluor 594-conjugated (all Invitrogen), and/or Mouse Cy3-conjugated (Jackson ImmunoResearch) as appropriate. For detailed visualization of actin structures, cells were stained with Phalloidin Alexa Fluor 488-conjugated (Invitrogen) or SIR-Actin (Spirochrome) as indicated, while Hoechst 33258 (ThermoFisher) was used for nucleus staining. Plates were finally mounted in Prolong Gold or Diamond antifade reagent (Life technologies). All cell lines (A2780s, H1299s, MEFs and TIFs) were fixed and stained using the same protocol.

Cells were imaged using a Leica TCS SP8 STED 3X microscope with an HC PL APO 100 x/1.40 oil objective using a HyD1 detector. A pinhole with 0.7 - 1 Airy units was used to further improve resolution while a notch filter was used for background reduction where possible. Images were captured using a white light laser (WLL) with excitation wavelengths 488, 550, 594 and 653 nm and appropriate emission spectra for green, cy3/red and far red respectively, while a Diode 405 laser was used with appropriate emission

spectra for blue. For whole cells, Z-stacks were captured covering the entire Z-profile of the cell with captured at intervals of 0.3 μm at a zoom of 1x to 1.23x. Zoomed rear images were all captured separately at higher resolution (i.e. smaller pixel size) as Z-stacks covering the entire Z-profile with 0.2 μm z-step and a zoom of 4x. Images were deconvolved post capture using Huygens professional software with default settings. All subsequent analysis and quantification was performed on maximum intensity projections (MIPs) in ImageJ; all representative images shown throughout are MIPs (pseudocolored using the red hot LUT where appropriate to reveal differences in intensity).

3D Spheroid Invasion Assay

Spheroids of N15A/A2780 cells were grown using the hanging drop method. Cells were detached by chelation using PBS containing 3 mM EDTA and 0.6 mM DTT (N15As) or trypsin (A2780s), pelleted by centrifugation and resuspended at ~800,000 cells/ml in 1ml of 20% (v/v) Methocel (Sigma) and 80% (v/v) complete appropriate medium (see Cell culture section). 20 μl drops of cell suspension (containing ~16,000 cells each) were pipetted onto the underside of a standard 10cm tissue culture plate (Corning), inverted to form hanging drops and incubated for 24h at 37 °C in a humidified 5% (v/v) CO2 atmosphere to allow formation of spheroids. 3-5 spheroids were extracted from the hanging drops by re-inversion and washing with complete medium using a Pasteur pipette, and resuspended in 100 μl collagen gel containing the following (all % denote v/v): 29.3% high molecular weight rat tail collagen (Corning); 0.7% 1M NaOH; 17.8% 10x PBS (Sigma); 3.6% 1mg/ml rat fibronectin (Sigma); 7.1% 1mg/ml laminin (Sigma); and 41.5% neat medium (Sigma). This collagen gel/spheroid mixture was reverse pipetted into a well of an 8-well μ-slide (Ibidi) and allowed to polymerize for 10 min at room temperature and a further 30 min at 37 °C in a humidified 5% (v/v) CO_2 atmosphere. Following polymerization, complete medium supplemented with 30 ng/ml EGF and 25 ng/ml HGF was added to the well of the μ-slide and incubated for 24h at 37 °C in a humidified 5% (v/v) CO2 atmosphere to allow time for cells to invade, before fixation for 15 min at room temperature in 4% PFA. Spheroids/cells were stained similarly as in the ‘Fixed cell high resolution imaging’ section above (doubling incubation times to allow for diffusion through the collagen gels).

Fixed samples were imaged by Spinning disc microscopy with a 20x/0.8 Plan-Apochromat air objective for zoomed out, edge of spheroid images with a Z-stack covering 200 μm with slices every 10 μm, while higher resolution Z-stacks of individually invading cells were imaged on the same microscope with a 63x/1.15 LD C-Apochromat Korr water objective with slices every 1 μm. All analysis was performed on the higher resolution images, MIPs shown.

Correlative Light and Electron Microscopy

For performing CLEM of polarized caveolae, we used 35 mm glass bottom dish (MatTek P35G-1.0-20-C) for confocal imaging, allowing the selection of cells with polarized caveolin-1 (mCherry-Cav1). Low-magnification images were acquired for mapping the position of the cell in relation to the micropatterns visible using transmitted light. Once imaged, cells were first fixed in Glutaraldehyde 2.5% + paraformaldehyde 2% in PHEM buffer (0.1M) for 1h on ice. A secondary fixation of 0.05% Malachite Green + 2.5% Glutaraldehyde in cacodylate buffer (0.1M) was performed (for 25 min on ice) before a post-fixation with 1%OsO4 + 0.8% KFe(CN)6 in cacodylate buffer (0.1M) (for 30 min on ice). After 3x10 min water rinses, cells were stained with 1% uranyl acetate. The cells were dehydrated in sequential gradient alcohol baths and infiltrated with Epon (resin). Pipette tips were mounted over the cell of interest following overnight polymerization at 60°C. The following day, the tips were filled with Epon and polymerized again overnight at 60°C. For relocating the region of interest, the surface of the resin block was imaged using a stereomicroscope allowing accurate positioning of the cell of interest according to the micro-pattern. Precise trimming was performed around the cell of interest. Finally, the resin block was serially sectioned (thickness: 100 nm) and all the sections were collected on electron microscope slot formvar grids (65 sections, distributed over 13 grids). Sample sections were imaged on a transmitted electron microscope (Hitachi). Transmitted electron microscopy images were stitched, aligned, and stacked with TrakEM2 (Cardona et al., 2012). 3D rendering of the electron microscopy acquisitions was performed with Amira aviso software.

AFM experiments were conducted on a Nanowizard® 3 atomic force microscope (JPK Instruments AG), which was built onto an LSM 880 confocal microscope (Carl Zeiss AG). Colloidal probes for all experiments were prepared as follows. 10μm diameter polystyrene beads (Polysciences) were resuspended in ethanol before a drop was applied to a clean microscope slide. Epoxy resin was mixed, and a 20μl pipette tip used to apply a small amount of resin to the slide. A tip-less cantilever (Arrow™ TL2, NanoWorld) was then mounted onto the AFM, and the slide mounted onto the stage. Using the AFM, the cantilever was first lowered onto the epoxy resin, with any excess resin being removed by tapping the cantilever onto the glass surface. The cantilever was then lowered into contact with a single bead and raised, with a visual inspection via brightfield to confirm bead attachment and desired placement. Once attached, the resin was left to dry in air for a minimum of 20 min. Colloidal probes were used within 1 day of preparation.

Correlative Light and SBFSEM

Cells imaged on CDMs in gridded glass bottom plates were processed for SBFSEM using a high-density staining method involving reduced osmium, thiocarbohydrazide, osmium, uranyl acetate and lead aspartate(Williams et al., 2011). The samples were dehydrated in staged ethanol (30, 50, 75, 90, 100, 100%) and then infiltrated with a mixture of Epon 812 (TAAB Laboratory Supplies Limited, UK) and ethanol (25% resin overnight, 50% resin for 8 h, 75% resin overnight). The next day the 75% resin was removed carefully with blue roll and rinsed twice with warm fresh 100% resin in order to remove the ethanol. The cells were left to infiltrate in 100% resin for 5 h. Cells were embedded in fresh resin by part curing a thin layer of resin for 4 h, then applying a BEEM capsule with the tip removed (Agar Scientific, UK). The BEEM capsule was filled with fresh resin to form an extension to allow easier

manipulation. The resin was cured at 60°C overnight. The glass coverslip was separated from the resin by freezing for a few hours then snapping off the embedded cells leaving the imprint of the etched glass on the surface of the resin. Using the imprint of the finder grid it was possible to localize the stained cells of interest. These were crudely excised using a hacksaw and attached to 3view pins (EM Resolutions, UK). Once attached to the pin they were trimmed more carefully for examination with a Gatan 3view (Starborg et al., 2013). Note that the cell layer was never perpendicular to the cutting face, but this meant that it was possible to setup the microscope focus and imaging conditions after trimming the peak of the block. The rest of the sample was left under the surface until the correct depth had been cut. The block was coated in gold palladium in order to reduce charging in the microscope, thus in order to localize the cells of interest the pre-coated trimmed blocks were imaged using a dissecting microscope. The dimensions of the block face and relative position of the cells could then be mapped and this information transferred onto the EM block face image. Serial block face runs were collected using a Gatan 3view (Gatan Inc) within an FEI Quanta 250 FEG (Thermo Fischer Scientific) scanning electron microscope at 3.8kV using 12nm pixels and 50nm cut thickness chamber pressure 0.4 Torr.

Generation of Rose Plots for Directional Analysis

Angles of Cav-1 peak, leading edge protrusion and rear location in live experiments were manually calculated using the ImageJ angle tool by creating a segment from drawing a line parallel with the idealized direction of stiffness gradient (i.e. exactly right to left in all shown soft – left to stiff – right images) to the point of interest – Cav-1/Actin protrusion peak – and then a line from this point of interest to the center of the nucleus: i.e. a point of interest exactly left of, right of, above or below the center point would give angles 0, 180, 90 and -90° respectively. Rose plots were created in MatLab, angles were binned into twenty 18° segments; relative sizes of triangular segments are directly proportional to the number of cells with angles contained within each bin.

Cell Region Based Quantification

For quantification of localized Cavin-1 intensity, whole cell images were analyzed with the interactive 3-D surface plot v2.31 in ImageJ using a 128 grid size and smoothing factor of 3.0. Under these conditions, the peak intensities of the rear and non-rear or rear and nuclear cell regions were manually identified and the ratio rear peak / non-rear peak or rear peak / nuclear peak calculated, therefore providing inherent normalization avoiding staining/expression discrepancies. For quantification of actin structures, a similar 3-D surface plot approach was used to manually identify the rear peak and leading edge peak of phalloidin 488 staining intensity to produce the normalized rear / leading edge ratio. For the quantification of localized GFP-membrane, GFP-Ezrin-WT and GFP-Ezrin-CA in live fluorescent cells, the interactive 3-D surface plot approach was similarly used on the first time-frame to measure the rear and front peaks of cells. These values were then normalized by dividing by the average of the rear and front peak intensities, such that when linked, pairwise front and rear values are plotted, all lines pass through the normalized peak intensity value 1.0. For Ect2, the normalized value for nuclear staining was additionally measured and normalized linked data of rear, nucleus, and front shown. For line profiles of Cavin-1 intensity, the line profile tool was used in ImageJ: representative lines of length 14.8021 μm were used from the rearmost point of the cell through towards the cell middle in zoomed images. Absolute Cavin-1 intensity was then quantified every 0.0507 μm (pixel width), smoothed by averaging with the neighboring 5 pixel values and the average of all cells plotted with SEM. Cavin-1 peak intensity position was determined by identifying the distance from the rearmost cell point of the single, non-smoothed pixel with the highest Cavin-1 value.

Quantification of Localized Adhesion Numbers

A2780 cells transiently transfected with EGFP-Paxillin and mCherry-Caveolin-1 were imaged for 5 min (1 min per frame) with 9 Z-slices with a step size of 1 μm. Adhesions were manually identified by observing high intensity paxillin regions which persist > 2 frames within individual Z-slices, and all such adhesions were counted across all Z-slices (where it was assumed that the step-size was large enough to avoid duplicate countings). Adhesions behind the rearmost boundary of the nucleus were assigned as ‘behind’, while all other adhesions in advance of this boundary were counted as ‘in front’ relative to nucleus position.

Reversible Live Hypo-osmotic Shock

For live imaging experiments using the reversible hypo-osmotic shock, cells expressing Emerald lifeact + mCherry-Caveolin-1 were imaged in isotonic control medium (1x Opti-Klear with 10% (v/v) FCS) as outlined in the section above for 10 min using point visiting, capturing at 1 min intervals. The medium was then rapidly (~4 min) replaced using an aspirator and 1ml pipette with 50% 1x Opti-Klear + 50% distilled water and all cells were subsequently imaged for 10 min in osmotic shock conditions. The osmotic medium was then rapidly (~4 min) replaced with isotonic 100% 1x Opti-Klear using an aspirator and pipette and imaged for a further 20 min. As a result, the same cells were imaged and analyzed in isotonic conditions pre-shock, osmotic shock conditions, and isotonic conditions post-shock.

For quantification of Cav-1 intensity during the reversible hypo-osmotic shock assay, the intensity of mCherry-Caveolin-1 expression was measured in manually drawn regions at the rear and in the center of imaged cells using ImageJ software. The same drawn ROIs were moved according to cell translocation so that the intensities were determined for the 11 timepoints pre-shock, the 11 timepoints under shock conditions and the 11 timepoints post-shock. All measured intensities were background subtracted and normalized to the intensity at the first timepoint t = 0, such that normalized timecourse profiles for the rear and central regions of all cells for

the full > 30 min of the assay were generated. Each timepoint was then averaged across all cells analyzed and the average timecourse profiles for all rear and all central regions was plotted with SEM bars shown.

For live analysis of Cav-1 accumulation at the rear of cells, osmotic shock was used to induce cells into a state with initially low rear Cav-1 (by incubation in osmotic medium for ~10 min) before the medium was replaced with isotonic medium via rapid aspirator pump and immediate pipetting. Throughout this process, laser-based (point visiting) Definite Focus was used via Slidebook software such that cells were captured 30-60s post media change without need for manual refocusing. Cells were imaged for a further >10 min using Definite Focus. For quantification, the segmentation tool used for FRET analysis (above) was adapted to create a binary mask using the mCherry-Cav-1 staining. The mean average intensity of the mCherry-Cav-1 intensity within the rear 10% and remaining 90% within the binary mask corresponding to the whole cell was then automatically measured for the pre and all post media change time-points, and normalized upon division by the maximum value (such that all other values < 1). The average time-course lines for the normalized average Cav-1 intensity were plotted for the rear and rest of cell regions, with quadratic curves fitted in GraphPad Prism to indicate a smooth, quadratic accumulation of Cav-1 at the cell rear.

Hypo-osmotic Shock of Fixed Cells and FRET

Cells expressing Raichu-RhoA were imaged in control medium as outlined in the section above for 5 min. The medium was then rapidly replaced with 50% Ham F12 + 50% distilled water and cells were subsequently imaged between 10–30 min post osmotic shock. Quantification of cell rear movement and FRET ratiometric activity were performed as before. For fixed cell imaging experiments using hypo-osmotic shock, cells were seeded on CDMs and left to spread for ~4h. The medium was then replaced with either normal growth medium for the control case or 50% medium + 50% distilled water for the hypo-osmotic shock and incubated for 10 min before being fixed, stained, and imaged as above.

Live Y27632 and H1152 Treatment

For experiments using live Y27632 treatment, cells expressing mCherry-Caveolin-1 were captured in the control state across a period of 5 min, treated with Y27632 via direct addition to the medium, rapidly refocused within ~ 5 min and the same cells imaged for a further 1h, capturing every 30s. Cells were also left for 1h without treatment to affirm that rear Cav-1 loss was not as an artefact of longer term imaging. For quantification, the rear / non-rear ratio of Cav-1 intensity was calculated using the 3-D surface plot in ImageJ (see cell region based quantification section above) for the first time point before Y27632 treatment and for a randomly chosen time point between 30 and 60 min post treatment, averaged across all captured cells and compared.

For live H1152 treatment, cells expressing Emerald-Lifeact and either control or Cav-1 siRNA-A were imaged for 5 min, treated with H1152 via direct addition to the medium, then imaged for a further 5 min. Throughout, Definite Focus was used to avoid any manual refocusing time, such that frames were taken every 30s before, (during) and after drug treatment. The forward translocation of the rear was manually measured for both transfection conditions without (before) and with (after) H1152 treatment, where negative values correspond to backwards movement.

Global and Caged Cytochalasin-D Treatment

For global Cytochalasin-D treatment, cells expressing GFP-lifeact + mCherry-Caveolin-1 were treated with cytochalasin-D at 2 μM and imaged 10 min following treatment with typical capture conditions. Photo-activatable 'caged' Cytochalasin-D was produced in the del Campo lab (manuscript in submission) and used as follows: Cells expressing GFP-lifeact + mCherry-Caveolin-1 were first treated with 50 μM immediately prior to capture. A 5 x 5 μm ROI was drawn using Slidebook 6.0, positioned such that the ROI was completely outside the cell area (as determined by lifeact expression) but within ~ 10 μm the rear of the cell (and such that the rear was the closest part of the cell to the ROI). After 2 fluorescent frame captures using the 488 nm and 561 nm lasers (to ascertain the GFP-lifeact and mCherry-Caveolin-1 expression pre photo-manipulation) the 405 nm emission photo-manipulation laser was used to illuminate each pixel in the previously drawn ROI for 10ms each (meaning the whole region was photo-activated for a total of ~6.5s since there are 5.12 pixels per micron). Cells were then imaged for a further 10 min with normal fluorescent conditions (as it was deemed that with captures longer than 10 min the activated Cytochalasin-D may diffuse to other areas of the cell). For the control comparison, cells were subjected to exactly the same photo-manipulation protocol in the absence of any drug treatment.

For quantification, the average mCherry-Caveolin-1 intensity within a manually drawn region at the rear of the cell pre-photo-manipulation (called rear pre), a region at the rear of the cell 10 min post-photo-manipulation (rear post), a region at the center of the cell pre-photo-manipulation (center pre), and a region at the center of the cell 10 min post-photo-manipulation (center post) were all measured using ImageJ and background subtracted. The ratio ((rear pre)/(rear post))/(center pre)/(center post)) was then calculated and used as a single metric to describe rear Cav-1 loss upon caged Cytochalasin-D treatment, where the treated cells were compared to untreated (but photo-manipulated) cells.

Model Construction

Our model of rear retraction incorporates 19 variables of which 12 are individual proteins (shown in green, yellow or cyan), 6 are empirical entities pertaining to stiffness, tension, polarization, actin alignment and retraction itself (in orange, pink or red) and the caveolae complex simplified to a single node. While all variables colored orange, yellow, pink or red - and reactions there-between - are included based on findings/hypotheses confirmed or originated in this paper, variables colored green or cyan are included to augment our study based on literature evidence. Green nodes link caveolae to RhoA activity while cyan nodes are intermediaries

between ROCK1/PKN-2 activation and actin alignment/MLC phosphorylation. We included Src, the GTPase activating protein (GAP) p190RhoGAP and Ect2 as the RhoA guanidine nucleotide exchange factor (GEF) as ‘green nodes’ with activation/inhibition reactions as shown since: (i) Cav1 can interact with RhoA (Gingras et al., 1998) but shows no structural evidence of being a GEF (Lamaze and Torrino, 2015) so naturally must act via a GEF for GTP hydrolysis of the small GTPase; (ii) Cav1 can interact with the RhoA activator/GEF Ect2 and control the localization and activity of a RhoA inactivator/GAP p190RhoGAP to regulate RhoA activity (Echarri and Del Pozo, 2015); and (iii) Src can phosphorylate Cav1 on Y14 to influence Cav1 expression and promote caveolae formation (Echarri and Del Pozo, 2015), or oligomerization and release (Zimnicka et al., 2016). Src also controls p190RhoGAP activity which directly inhibits RhoA (Grande-García and del Pozo, 2008), and pY14-Cav1 binds to and activates Csk, a ubiquitously expressed inhibitor of Src (Grande-García and del Pozo, 2008). DRF (Diaphanous-related formin), Limk, Cofilin, CPI-17 and MLCP (Myosin-light-chain phosphatase) and activation/inhibition reactions there-between were included as ‘cyan nodes’ based on the strong evidence that: (i) Formins are always required to some extent for efficient F-actin polymerization and can be activated directly by RhoA or ROCK1 (Breitsprecher and Goode, 2013); (ii) ROCK activates LIM-kinase 1 by phosphorylation at threonine 508 within the activation loop (Ohashi et al., 2000); (iii) Limk phosphorylates cofilin at Ser 3 both *in vitro* and *in vivo* which abolishes the ability of cofilin to bind and depolymerize actin (Yang et al., 1998); (iv) Cofilin universally disassembles actin filaments so acts as an inhibitor to rear actin alignment (Carlier et al., 1997); (v) Phosphorylation of CPI-17 at Thr(38) by PKN2 or ROCK1 enhances the inhibitory potency of CPI-17 towards MLCP (Hamaguchi et al., 2000); and (vi) the primary function of MLCP is to oppose myosin phosphorylation whereby kinase and phosphatase activities are mutually antagonistic and in competition (Morgan et al., 1976).

ODEs Formulation

The ordinary differential equations (ODE) model was built with the following specifications:

(i) All reactions use mass-action kinetics; this is the simplest kinetic scheme to use and in the absence of either more detailed numerical data or suggestions of other more relevant dynamics at play in vitro, is the most sensible starting point for a kinetic model.

(ii) All variables, both proteins and biophysical entities, can be in an active, inactive or bound state with a conserved moiety; proteins can certainly be active, inactive or bound in cells. The biophysical entities are assigned an ‘active’ or ‘high’ state, and an ‘inactive’ or ‘low’ state to be consistent with the protein variables within the same model as convention, although the ‘inactive’ or ‘low’ states can be regarded as dummy variables, while the ‘high’ states are the actual outputs of interest. For example, the ‘high rear retraction’ corresponds to the instantaneous velocity of the rear, while the ‘high polarized substrate stiffness’ input corresponds to the steepness of theoretical gradient of the substrate. This also ensures all variables remain finite, see below.

(iii) The value of all variables is normalized and confined between 0 and 100 units for all time; This ensures that the value of all variables in the model remain finite (i.e. that a cell cannot express an infinite amount of protein or that rear retraction cannot be infinitely fast which would be biologically implausible). This normalization is such that protein and biophysical entity levels can be comparable and conceptualized on the same scale, while also conveniently overcomes the lack or real experimental expression level data.

(iv) Reactions involving two proteins are two step processes whereby the proteins first bind together then the activation state of one/both proteins change/s as desired; evidence suggests for many protein-protein interactions that activation or inhibition requires binding of the proteins before a state change, for example The GEF Ect2 binds to GDP bound RhoA before promoting the switch to the GTP active form of RhoA.

(v) Reactions involving at least one biophysical entity are one step processes whereby the activation state of at least one variable involved in the reaction changes directly as desired; it makes no sense to conceptualize that polarized contractility binds to high membrane tension for example, therefore one step state changes are sufficient for the required kinetics.

(vi) All reactions which only involve proteins or only involve biophysical variables are reversible where the backwards direction is at most 10% the rate of the forward direction, decreased tension activating caveolae and aligned Actin and p-MLC activating Acto-myosin contractility are non-reversible; Reversibility of protein-protein interactions are well reported, keeping most reactions as reversible to a small extent prevents an implausible high resting state, while a biophysical entity relapsing to a protein or vice versa is conceptually impossible.

(vii) All proteins and biophysical entities are subject to a turnover term, whereby there is a constant flux from the active state to the inactive state for all variables when not explicitly activated upstream. Without turnover and/or reversibility, variables such as rear retraction stay in an active, high level steady state despite lack of active upstream activators which is physiologically unrealistic: rear retraction requires Acto-myosin contractility and aligned Actin constantly and this formulation ensures this.

Note since the potent inhibitor of p-MLC, MLCP is already included explicitly in the model, and these are almost like two forms of the same protein, the turnover of p-MLC is not included. To balance this and since p-MLC is not a protein but a specific state thereof, the activation of p-MLC is one-step rather than two step binding then activation. Implementing p-MLC with binding and turnover does not alter general findings of the model provided rates are balanced accordingly.

Initial Conditions

For unperturbed simulations, the initial conditions (ICs) used are the input ‘polarized substrate stiffness’ set to the maximum 100, active Src set to 50, active Cofilin and MLCP set to 100, while the active form of all other is set to 0. The polarized substrate stiffness IC was taken as it is the nominated external input of the model, required to initiate the proceeding dynamics downstream. Cofilin and MLCP were set to 100 since these are inhibitory variables with no explicit activators, such that the only way these can be active is when they start active, while Src is set at 50% active initially since it has no upstream activator but has both an activator and inhibitory role on downstream effectors.

Parameter Values

All parameter values were based on order of magnitude predictions, i.e. were subject to the constraint that all values were $1 \times 10^{-n}$for an integer n greater or equal to 1. The order of magnitude approach was adopted since the appropriate kinetic data were unavailable, and more specific rates would not be justifiable and may promote an over-determined model. All rates were checked with half and double values, and the general topology and behavior was unchanged. Parameters were initially set based on literature findings and certain assumptions and then iteratively, improved based on biological plausibility of simulated outputs. In particular, it was assumed that protein-protein interactions occur with higher rates than those involving biophysical entities because: (i) protein-protein interactions are two step (binding then state change) so are already going to be slower overall, and (ii) it is known that protein-protein interactions such as binding can be incredibly quick *in vitro*, while the precise kinetics of polarized contractility leading to decreased tension for example are likely to be slower as these events are more complex at the molecular level. All parameter rates used can be seen in the full list of ODEs used in the BioModels database (see below).

Model Simulation and Perturbation

The model was built and simulated using Copasi software version 4.15 (Hoops et al., 2006). Deterministic simulations were performed using the LSODA method for the first 10000 seconds with an interval size of 25s. The time-course outputs for all variables in the model were checked for plausibility; however, the outputs for a chosen subset of variables are shown for simplicity. For *a priori* knockdown simulations, all variables were kept the same as the unperturbed simulation apart from the initial amount of the relevant variable (RhoA, and Cav-1), which were both reduced to 12% of total concentration. For substrate transition simulations, ‘polarized substrate stiffness’ was assigned to change from 100 to 0 or 0 to 100 at t = 4000s. For simulation of the osmotic shock assay, the rate of transition from decreased membrane tension to increase membrane tension was increased 1000-fold (from 0.01 to 10) at time t = 4000s, and then returned to the original value (0.01) at t = 7000s to mimic return to isotonic conditions. For mimicry of Y-27632 treatment, an event at t = 5000s was added to the simulation where the turnover rate for ROCK1 and PKN2 was increased 1000-fold (from 0.01 to 10); for ‘global’ cytochalasin-D treatment, the turnover rate of F-actin (i.e. the rate at which F-actin transitions to G-actin) was increased 100-fold (from 0.01 to 1) while for ‘caged’ cytocholasin-D treatment the turnover rate of F-actin was increase 20-fold (from 0.01 to 0.2), both events occurring at t = 5000s.

Parameter Sensitivity Analysis

Parameter sensitivity analysis was performed using the built-in Copasi tool with Retraction/Recoil as the target and all rates in the model as the analyzed objects. Scaled parameter analysis was run, such that the concurrent rates used in the model were factored in to the apparent importance of each parameter, while the total of the scaled parameter sensitivity sums to zero.

QUANTIFICATION AND STATISTICAL ANALYSIS

Box and whisker plots show mean as ‘+’, whiskers generated using Tukey method. All statistics shown on box and whisker graphs with two conditions describe unpaired student t tests while for all box and whisker graphs with > two conditions a one-way ANOVA test for multiple comparisons has been used. For all linked line graphs concerning trap force data, pairwise Mann-Whitney tests were used to compare the front and rear membrane of the same cells directly, while data from all other linked line graphs have been subjected to paired T tests. All statistical analysis has been performed with GraphPad Prism software, where **** denotes $p < 0.0001$, *** denotes $p < 0.001$, ** denotes $p < 0.01$ and * denotes $p < 0.05$. All N numbers and p values are described in relevant figure legends.

DATA AND CODE AVAILABILITY

The SBML of the model described in this paper has been deposited in the BioModels database (Le Novére et al., 2006) (https://www.ebi.ac.uk/biomodels/MODEL1908290001) named ‘Hetmanski 2019 cell rear’ and can be loaded into Copasi 4.15 for reader editing/simulation purposes.

Developmental Cell, Volume *51*

Supplemental Information

Membrane Tension Orchestrates Rear Retraction

in Matrix-Directed Cell Migration

Joseph H.R. Hetmanski, Henry de Belly, Ignacio Busnelli, Thomas Waring, Roshna V. Nair, Vanesa Sokleva, Oana Dobre, Angus Cameron, Nils Gauthier, Christophe Lamaze, Joe Swift, Aránzazu del Campo, Tobias Starborg, Tobias Zech, Jacky G. Goetz, Ewa K. Paluch, Jean-Marc Schwartz, and Patrick T. Caswell

Supplementary information

Figure S1-S7

Video S1-7

Supplementary Figure Legends

Figure S1: 3D migration in fibrillar matrix; related to Figure 1

(A) A2780 cells seeded within 3D CDM. Representative single plane images above, through and below the cell (top) and an xz projection s (bottom), where each dashed line denotes the relative positions of the single plane images. Cells/matrix were fixed and stained for fibronectin and F-actin (SiR-actin) prior to imaging with a Z step size of 0.27 µm. (B) A2780 cells moving in an unperturbed CDM (left) or a locally softened CDM (right), representative maximum intensity projections (MIPs) shown. Cells were fixed and imaged as in A. (C) H1299 cells were seeded onto FN-coated 2D durotactic gradients for 16 hours and imaged, orientation of migration with respect to the gradient is shown by rose plot. (D) Left: A2780 cells expressing farnesyl-EGFP (GFPmembrane) were seeded onto 3D CDM and imaged as in Fig. 1A. Right: Pairwise quantification of normalised GFP-membrane peak intensity at the front and rear of A2780 cells (N=33 cells across 3 repeats). (E) A2780 expressing Lifeact-GFP stained with lipophilic dye (Dil) moving in 3D CDM. Timelapse of the cell rear captured using Zeiss Airyscan. (F) Trap force at the front and rear of unpaired cells on durotactic gradients, untreated glass and uniform stiff gels and single membrane measurements of unpolarised cells on uniform soft gels; N>31 cells per condition analysed across 3 repeats for gradient; N>18 per condition across 3 repeats for glass, N>12 cells per condition across 3 repeats for uniform stiff, N=6 cells across 2 repeats for uniform soft. (G) A2780 cells expressing EGFP-paxillin and mCherry-caveolin-1 were seeded in CDM for 4 hours then imaged for 5 minutes, 9 Z-planes imaged every 1µm by spinning-disc confocal microscopy, representative MIPs of merged (left) and paxillin (right) channels shown; Right: Pairwise quantification of number of adhesions in front/nucleus region compared to behind nucleus region, N=31 cells across 3 repeats. ****, p<0.0001.

Figure S2: Caveolae localization in cells in 3D matrix and durotactic gradients; related to Figure 2

(A) H1299 cells expressing Lifeact-mEmerald and mCherry-caveolin-1 were seeded in 3D CDM and imaged as in Fig. 2(B). (B) H1299 non-small cell lung cancer cells were seeded in 3D CDM and fixed and stained for F-actin and cavin-1. MIPs of confocal stacks are shown. (C) A2780 cells seeded on a CDM locally softened via trypsin beads, fixed as in B and stained for F-actin (SiR-actin) and cavin-1. Representative MIP shown. (D) A2780 cells in 3D CDM were fixed and stained for endogenous caveolin-1. Representative MIP of confocal stacks are shown. (E) H1299 cells expressing Lifeact-mEmerald and mCherry-caveolin-1 were seeded on 2D durotactic gradients and imaged as in Fig. 2(C). (F) A2780 cells on 2D soft, stiff, or durotactic gradients of stiffness (all coated with fibronectin) stained for F-actin (phalloidin) and cavin-1. Representative MIP shown. (G) A2780 cells on 2D soft, stiff, or durotactic gradients of stiffness (all coated with fibronectin) stained for F-actin (SiR-actin) and caveolin-1. Representative MIP shown. (H) Orientation of caveolin-1 in cells as in (F) with respect to the stiffness gradient (N=30 cells across 3 repeats). (I) Average speed of A2780, H1299, Telomerase-immortalized fibroblasts (TIFs) or mouse embryonic fibroblasts (MEFs) cells in 3D CDM during 16 hours timelapse. (J) Average intensity of mCherry-caveolin-1 at the rear of A2780, H1299, MEF and TIF cells moving in 3D CDM. (K) TIFs or MEFs were seeded in 3D CDM and fixed stained and imaged as in (B), representative images of poorly polarized highly spread morphology of TIFs and MEFs in 3D CDM. (L) Trap force measurements at the front and rear of elongated TIF cells, N=10 cells across 3 repeats. (M) TIFs and MEFs as in (K), displaying the polarised, shorter phenotype. (N) Morphology of TIFs and MEFs when undergoing rapid migration in 3D CDM. (O) Proportion of TIFs and MEFs that show elongated (typical) morphology versus shortened (rapid) morphology, and proportion of cells that exhibit high versus low/unpolarized cavin-1 at the cell rear. (P) A2780 cells expressing mEmerald-Lifeact and mCherry-caveolin-1 were imaged migrating in 3D CDM, and fixed and processed for Serial Block Face SEM (SBFSEM). The relative ease of data collection using SBFSEM meant that it was possible to generate correlative EM data from 9 different motile cells with sufficient resolution to allow identification of caveolae. A volume render of the EM data is compared with the light microscope image to show that the cell morphology has not been affected by fixation. The enlargements show evidence of

caveolae/multilobed caveolae (yellow arrowheads) at the rear of the cell, but no evidence of caveolae was seen in the peri-nuclear region. ****, p<0.0001.

Figure S3: Increasing membrane tension supresses rear caveolae assembly in durotactic gradients and CDM; related to Figure 3

(A) A2780 cells expressing Lifeact-EGFP and mCherry-caveolin-1 were seeded onto 2D durotactic gradients and imaged before and during osmotic shock treatment (0.5Xmedia). (B) A2780 cells were seeded onto CDM and treated with osmotic shock (0.5Xmedia) or isotonic media, before fixation, staining and imaging of endogenous caveolin-1. MIPs are shown. Yellow line indicates outline of the cell. Insets MIPs of the cell rear captured using high resolution imaging. (C) Distance of rear retraction of A2780 cells in isotonic versus hypo-osmotic shock in a 5 minute timelapse. N>61 cells per condition across 3 repeats. ****, p<0.0001.

Figure S4 Caveolae control rear retraction through RhoA-ROCK1/PKN2 actin organisation; related to Figure 4

(A) Caveolin-1 knockdown A2780 cells were plated in 3D CDM and speed of migration measured by timelapse microscopy over 16 hours. N=90 cells per condition across 3 repeats. (B) Representative images of control and caveolin-1 knockdown A2780 cells moving through 3D CDM. (C) Quantification of control and caveolin-1 knockdown cell length whilst migrating in 3D CDM. N=90 cells analysed per condition across 3 repeats. (D) Efficiency of knockdown of caveolin-1 in A2780 cells. (E) Efficiency of knockdown of caveolin-1 in H1299 cells. (F) Left: Lifeact-mRFP expressing, caveolin-1 knockdown H1299 cells were plated in 3D CDM, imaged by spinning disk confocal microscopy and retraction of the cell rear over 5 minutes measured. Right: Forward movement of H1299s cells over a 5 minute timelapse. N>74 cells per condition across 3 repeats. (G) Left: Control and EHD2 knockdown A2780 cells expressing Lifeact-EGFP and mCherry-caveolin-1 migrating in 3D CDM imaged by spinning disk confocal microscopy. Centre: Average migration speed of control and EHD2 knockdown cells migrating in 3D CDM for 16 hours. N=90 cells analysed per condition across 3 repeats. Top right: Distance of rear retraction of control and EHD2 knockdown A2780 cells in 3D CDM during a 5 minute timelapse N>80 cells per condition across 3 repeats. Bottom right: EHD2 knockdown efficiency in A2780 cells. (H) Left: Representative images of control and EHD2 knockdown A2780 cells with coloured bars indicating cell length. Right: Quantification of control and EHD2 knockdown cell length whilst migrating in 3D CDM. N=90 cells analysed per condition across 3 repeats. (I) Active RhoA location biosensor GFP-AHPH expressing A2780 cells were seeded in 3D CDM and fixed after 6 hours. F-actin was visualized with SiR-actin, and cells imaged at high resolution. MIPs are shown. (J) Active RhoA location biosensor GFP-AHPH expressing A2780 cells were seeded on 2D durotactic gradients for 16 hours and imaged by spinning disk microscopy using a long working distance 63x objective. (K) Active RhoA location biosensor GFP-AHPH and mCherry-caveolin-1 expressing A2780 cells were seeded in 3D CDM and imaged by spinning disk confocal microscopy after 6 hours. (L) Raichu-RhoA in osmotic shock. (M) Left: Control or RhoA knockdown cells were seeded into 3D CDM and imaged by spinning disk confocal microscopy after 4 hours. Single z-slice stills from movies are shown 300s apart. Centre: Forward movement of control and RhoA knockdown cells over a 5 minute timelapse. N>54 cells per condition across 3 repeats. Right: RhoA knockdown efficiency in A2780 cells. (N) Control, ROCK1 or ROCK2 knockdown cells were seeded into 3D CDM and fixed after 4 hours. F-actin was visualized using SiR-actin and confocal imaging. MIPs are shown. (O) A2780 cells were seeded into 3D CDM and treated with Y27632 (10 µM) for 2 hours before fixation. F-actin (phalloidin) was visualized by confocal microscopy, MIPs shown. (P) Left: Speed of migration of A2780 cells in 3D CDM over 16 hour timelapse was analysed in control, ROCK1, ROCK2 and ROCK1+2 knockdown cells. N=90 cells analysed per condition across 3 repeats. Right: Efficiency of ROCK1 and ROCK2 knockdown in A2780 cells. (Q) Top left: Speed of migration of A2780 cells in 3D CDM over 16 hour timelapse was analysed in control, PKN1, PKN2 and PKN3 knockdown cells N=90 cells analysed per condition across 3 repeats. Top right: Effect of PKN2 knockdown on architecture of F-actin at the rear of A2780 cells migrating in 3D CDM (representative high resolution MIP shown). Bottom left: Efficiency of PKN1, PKN2 and PKN3 knockdown in A2780 cells, assessed using a pan-pPKN antibody. Quantification of blots from 3 independent experiments shown in graph. Bottom right: Efficiency of PKN2 knockdown in A2780 cells, assessed using a PKN2 specific antibody. ****, p<0.0001; *, p<0.05.

Figure S5: Systematic identification of GEFs activating RhoA at the cell rear, related to Figure 5

(A) Quantification of average speed of control, Ect2, Vav2, ARHGEF2, DOCK1, ELMO2 and PLEKHG5 A2780 cells in long-term (16h) migration in 3D CDM, N>60 cells per condition across 3 repeats, dashed line indicates control mean. (B) Quantification of cell length ~20h after spreading for cells is in (A), N>60 cells per condition across 3 repeats. (C) Quantification of average speed of control and 2 individual Ect2 oligo knockdown A2780 cells, N=30 cells analysed per condition across 2 repeats. (D) Representative single control and Ect2 knockdown cells migrating in 3D CDM over 150 minutes. (E) Quantification of rear / front peak F-actin staining intensity of control, and Ect2 knockdown cells (individual oligos). (F) Efficiency of knockdown of Ect2, Vav2, ARHGEF2, DOCK1, ELMO2 and PLEKHG5 in A2780 cells. (G) Knockdown of Ect2 with individual siRNA oligos; #5 and #7 were used in subsequent experiments. ****, $p<0.0001$; *, $p<0.05$.

Figure S6: Model outputs are robust to wide parameter value ranges; related to Figure 6

(A) Summary parameter sensitivity analysis showing normalised effect of all rates in the model on the rear retraction output. Parameters which have > 0.02 positive scaled effect on rear retraction are shown in green, parameters which have < 0.02 negative scaled effect shown in red, while all other parameters with less drastic effects are represented by the blue bars. (B) Range of rear retraction temporal dynamics given halving and doubling of the 9 most influential rates in the model (6 positive regulators in green, 3 negative regulators in red) showing general model outputs are robust to even large parameter alterations.

Figure S7: Effect of RhoA knockdown and CytoD on caveolae localization; related to Figure 7

(A) Control and RhoA knockdown A2780 cells expressing mCherry-caveolin-1 were seeded into 3D CDM and imaged by spinning disk confocal microscopy after 4 hours. Single z-slice stills from movies are shown 300s apart. (B) Intensity of mCherry-caveolin-1 at the rear of cells as in (A) N=76 cells per condition across 3 repeats. (C) Control and RhoA knockdown cells were seeded into 3D CDM and fixed after 4 hours. Endogenous F-actin, caveolin-1 and cavin-1 were visualized, representative MIPs shown. (D) High resolution images of endogenous cavin-1 at the rear of control and RhoA knockdown A2780 cells migrating in 3D CDM as in (C). (E) Quantification of the average rear/front cavin-1 intensity ratio of control and individual oligo Ect2 knockdown A2780 cells. (F) A2780 cells expressing Lifeact-EGFP and mCherry-caveolin-1 were seeded in 3D CDM for 4 hours before and imaging in the presence of 2μM CytoD. (G) Caged CytoD is a photoactivatable derivative of the potent actin disruptor Cytochalasin D (CytoD), which possesses nitroveratryloxycarbonyl (Nvoc) photoremovable group located at the bioactive hydroxyl group of CytoD. The Nvoc group inhibits the binding activity of CytoD, which can be restored upon photolysis reaction with light exposure under microscopy setups. Upon light exposure, caged CytoD enables dosed delivery of CytoD in living cells at time scale of seconds and with subcellular resolution. (H) A2780 cells expressing Lifeact-GFP and mCherry-caveolin-1 were seeded onto 3D CDM and imaged after 4 hours in the absence of caged-CytoD before and after photomanipulation of a specific region behind the cell rear (indicated by yellow box, as in Figure 6G). ****, $p<0.0001$.

Supplementary Video Legends

Video S1: (Part I) A2780 cell expressing mEmerald-Lifeact migrating in 3D CDM over 10 minutes, with a Red-hot LUT applied. (Part II) A2780 cell expressing mEmerald-Lifeact migrating for 5 minutes in a non-uniform CDM with a (trypsinised) softened region to the left and the unperturbed stiffer region to the right. (Part III) A2780 cell expressing mEmerald-Lifeact migrating from soft (left) to stiff (right) on a gradient poly-acrylamide gel for 10 minutes. Related to Figure 1A, B and C respectively

Video S2: A2780 cell expressing farnesyl-EGFP (GFP membrane) migrating in CDM over 5 minutes. Related to Figure S1D.

Video S3: Single representative A2780, H1299, typical MEF, rapid MEF, typical TIF and rapid TIF migrating over 2 hours in CDM. Related to Figure S2I

Video S4: A2780 cell expressing mCherry-caveolin-1 (magenta) and mEmerald-Lifeact (green) imaged prior to fixation and processing for CLEM. Related to Figure S2P.

Video S5: A2780 cell expressing Cherry-Cav-1 migrating in CDM in isotonic medium for 10 minutes, then subjected to osmotic shock for 10 minutes before medium reversion to isotonic conditions for a further 10 minutes. Related to Figure 3C.

Video S6: A2780 cell expressing Cherry-Cav-1, migrating in CDM untreated for 5 minutes, then treated with Y-27632 and imaged for a further 60 minutes. Related to Figure 7A.

Video S7: A2780 cell expressing Cherry-Cav-1 (magenta) and eGFP-Lifeact (green) migrating in CDM treated with 'caged' Cytochalasin-D which is photo-activated at t = 30s within the yellow box region. Related to Figure 7H.

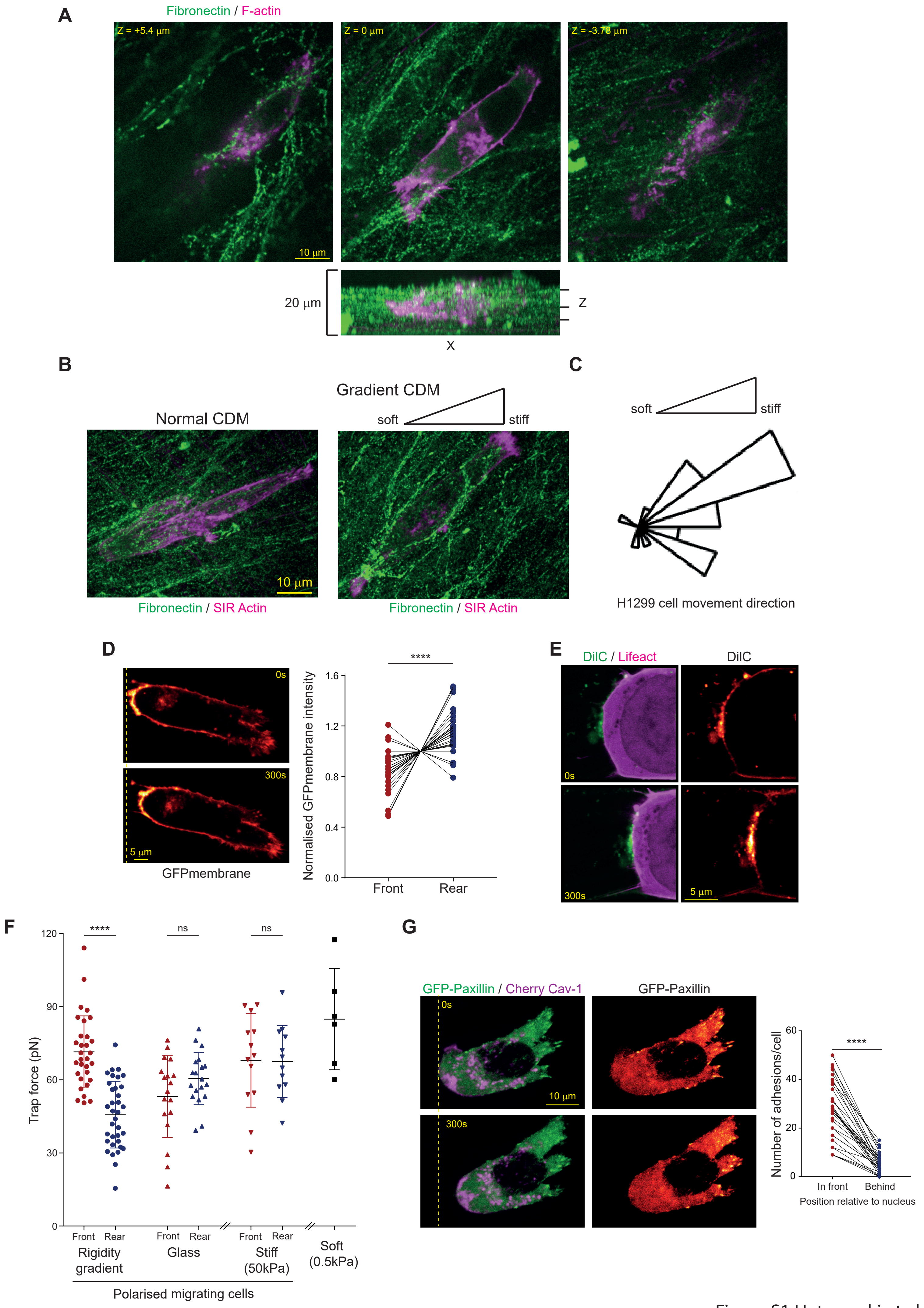

Figure S1 Hetmanski et al

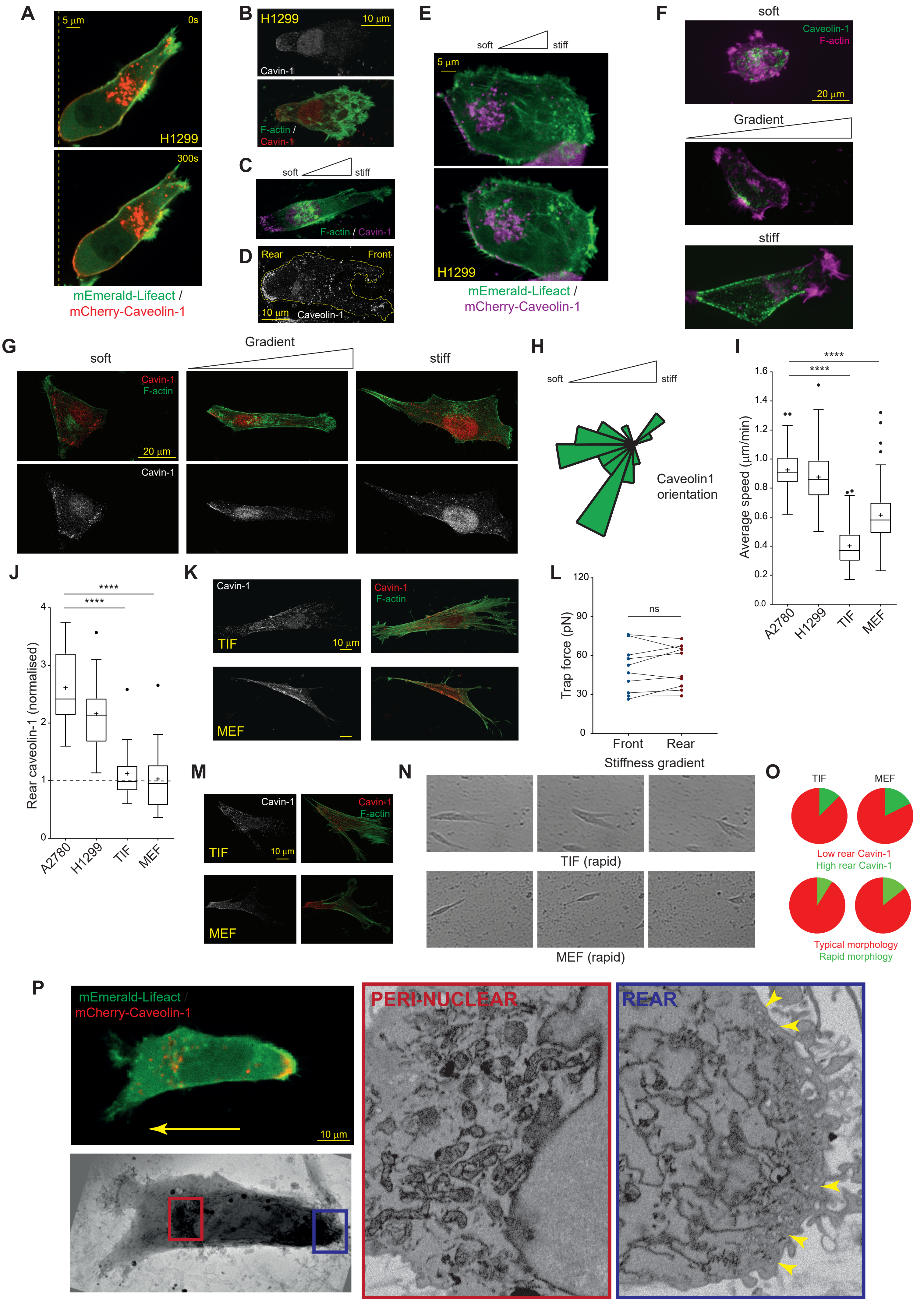

Figure S2 Hetmanski et al

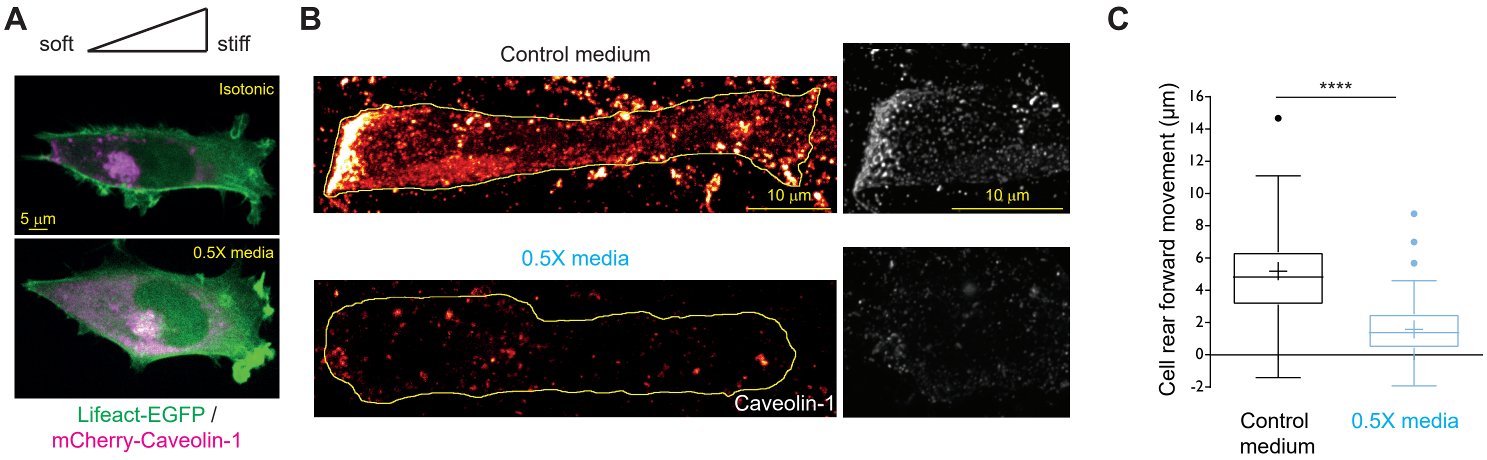

Figure S3 Hetmanski et al

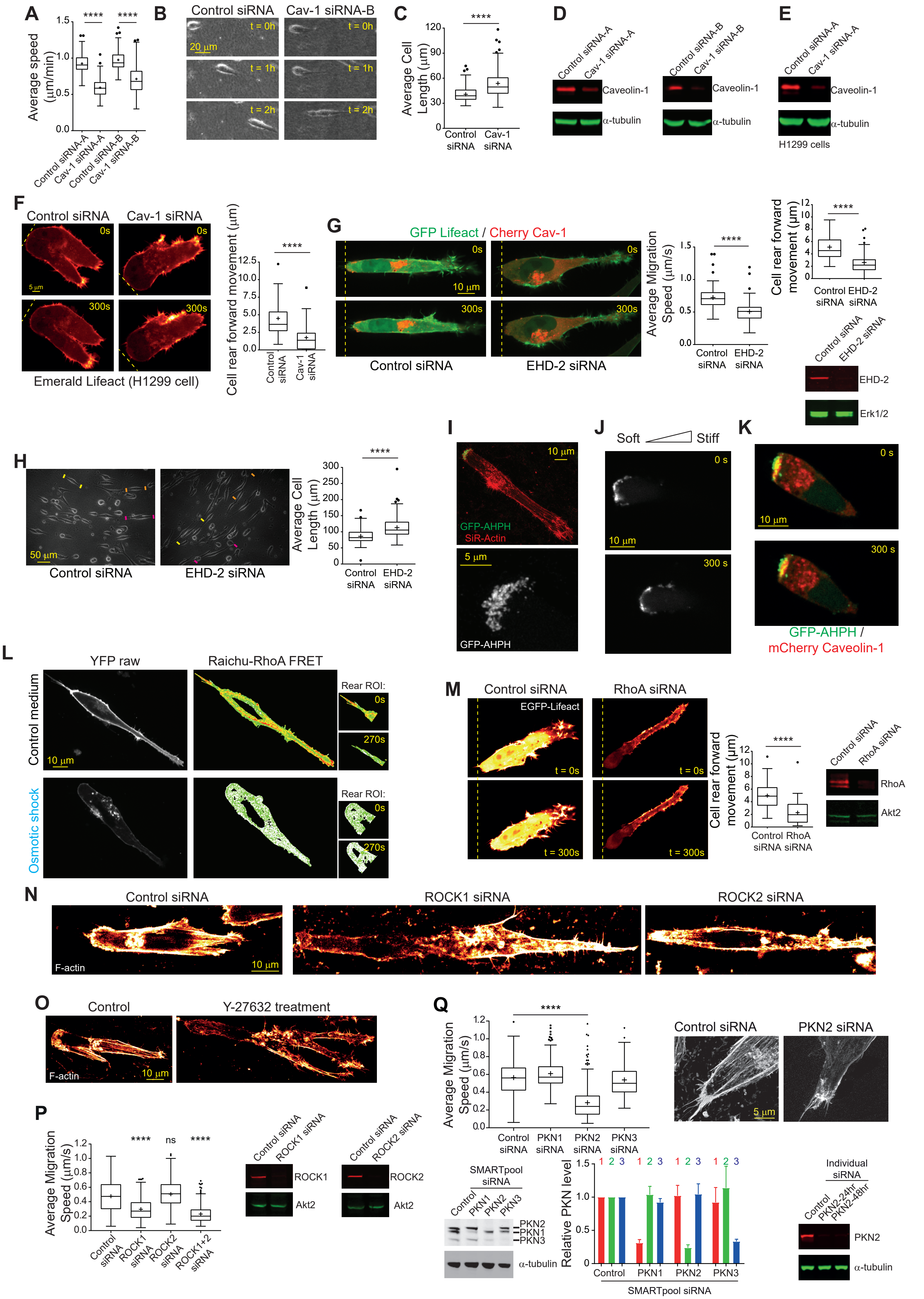

Figure S4 Hetmanski et al

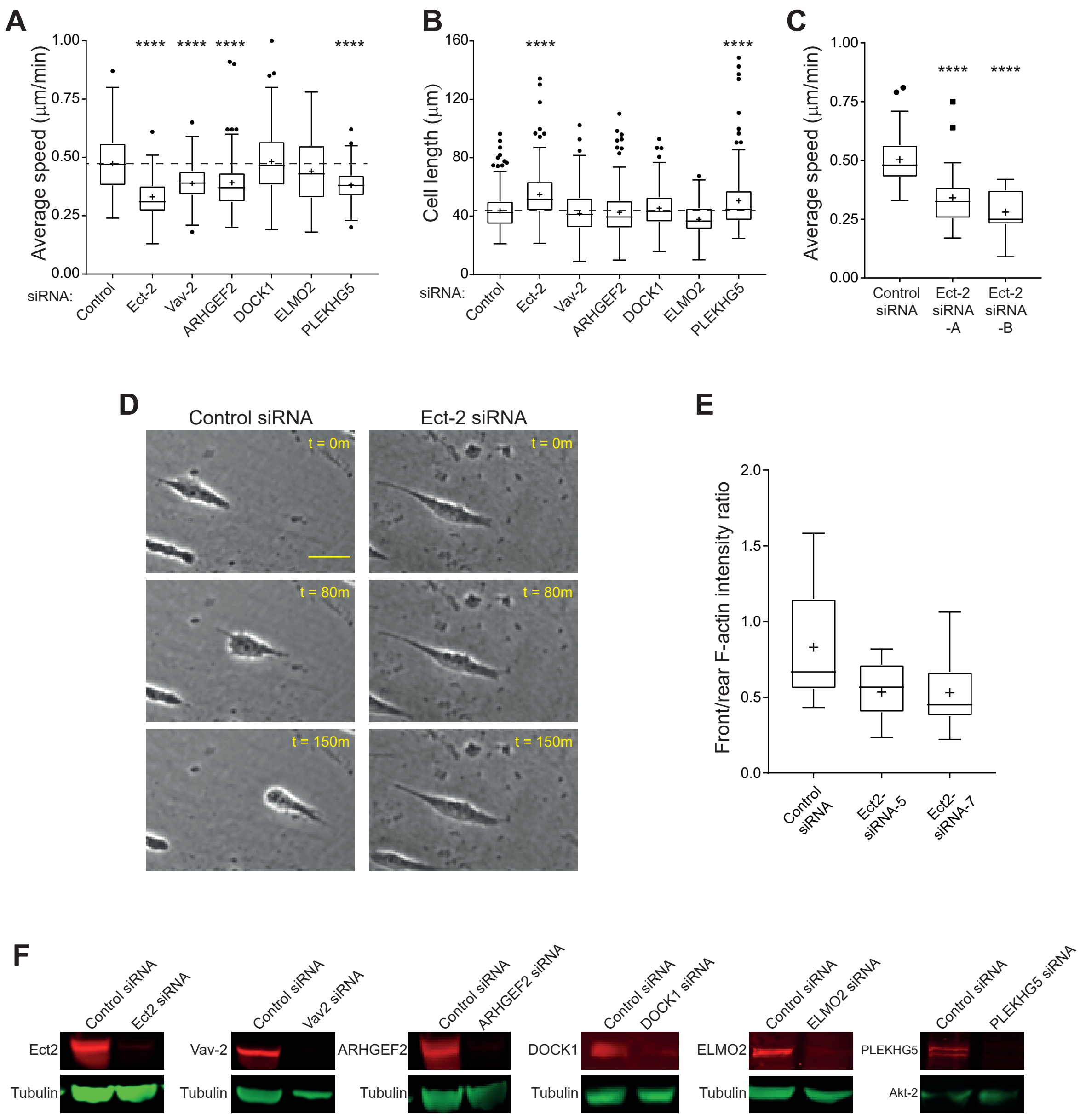

Figure S5 Hetmanski et al

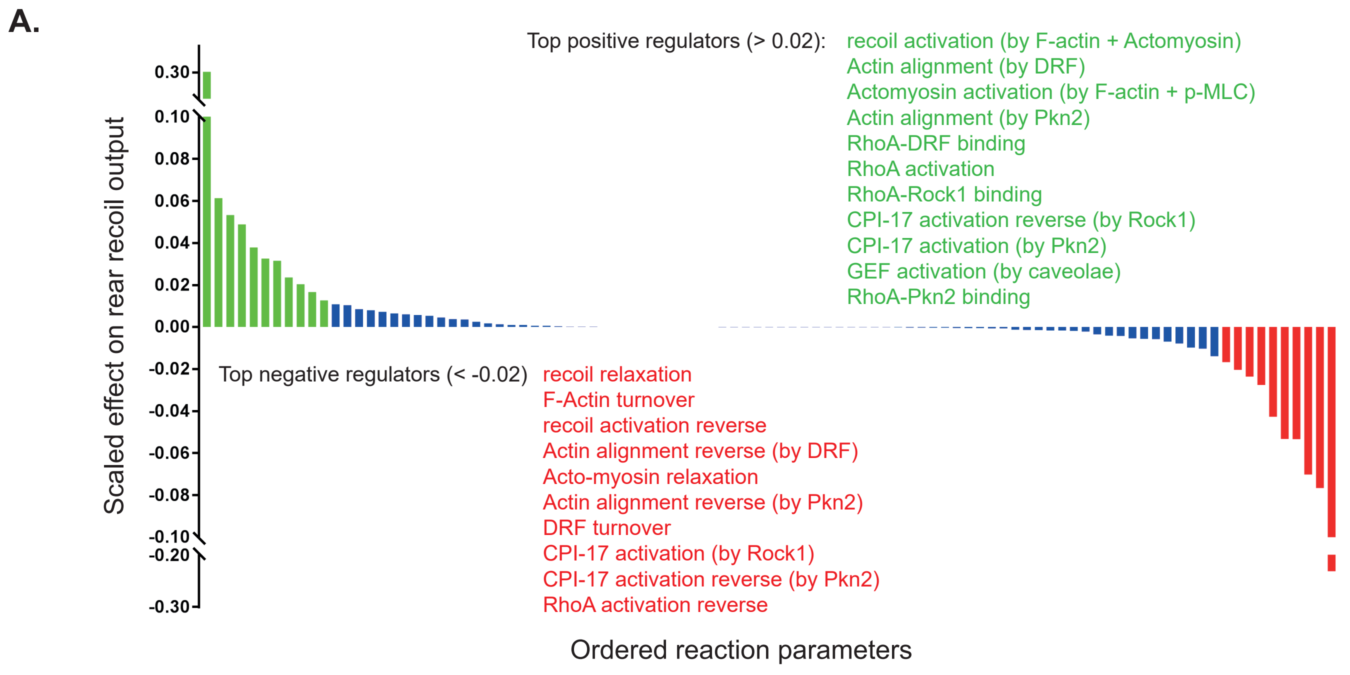

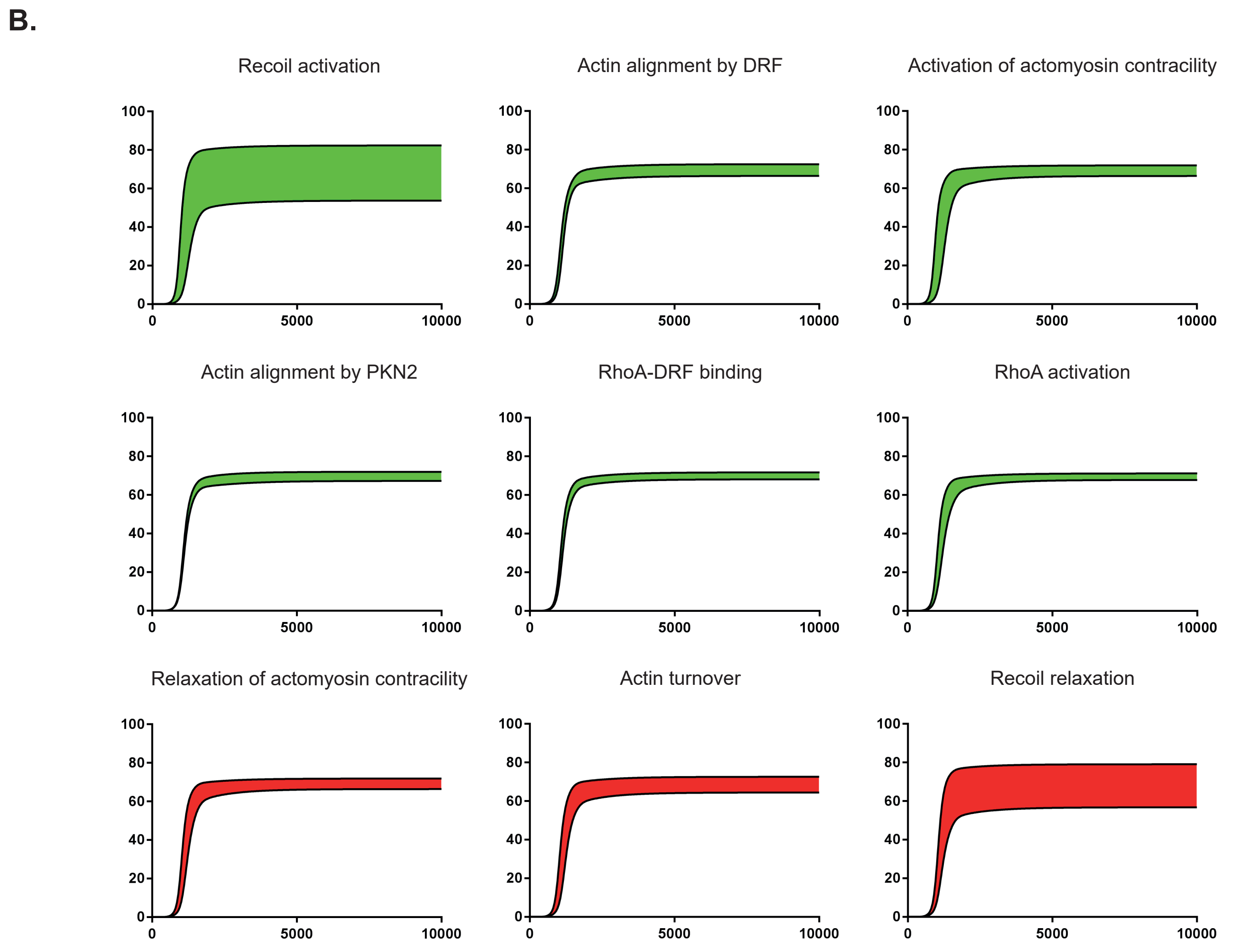

Figure S6 Hetmanski et al

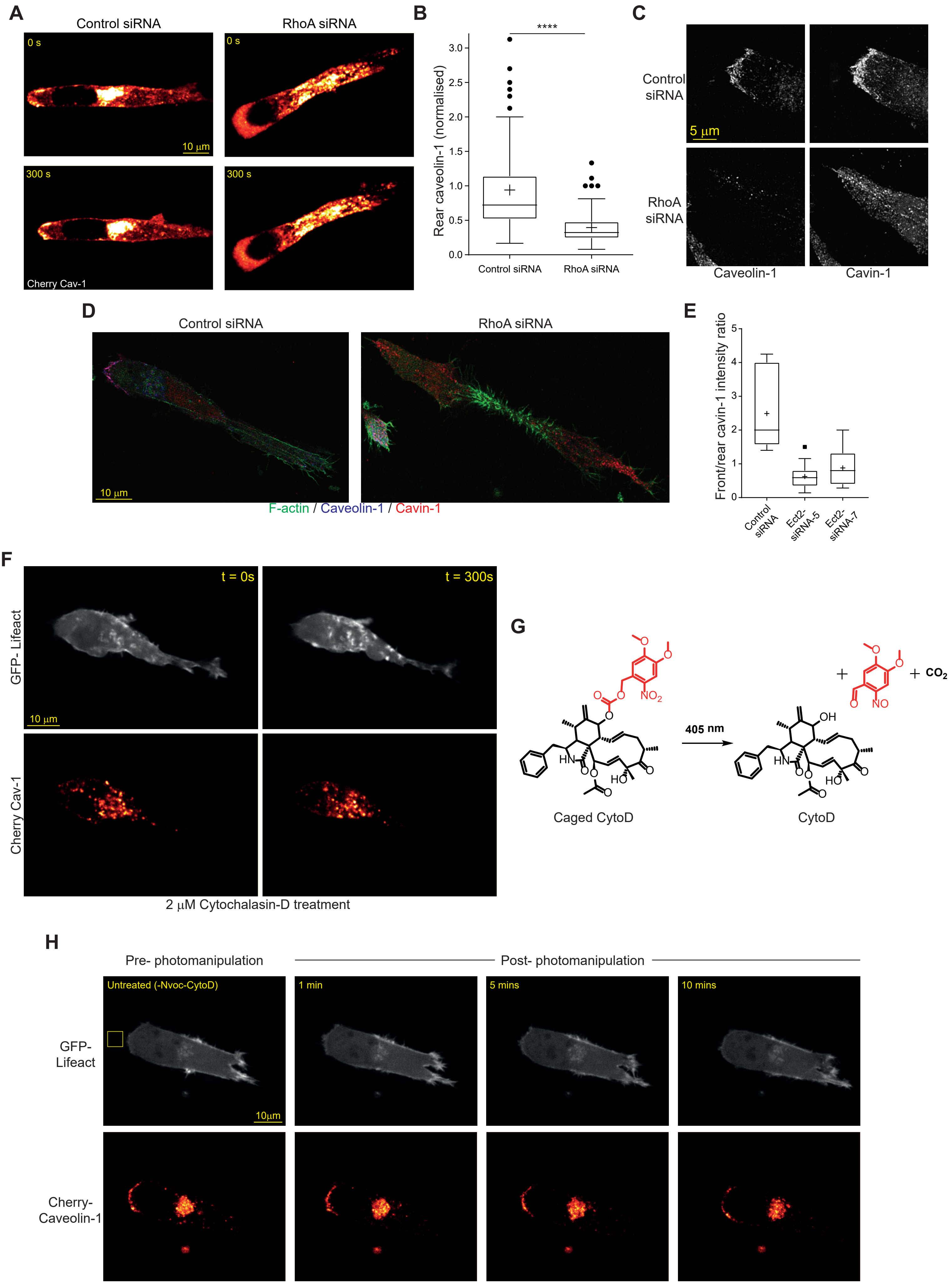

Figure S7 Hetmanski et al
